# What microglia depletion approaches tell us about the role of microglia on synaptic function and behavior

**DOI:** 10.3389/fncel.2022.1022431

**Published:** 2022-11-04

**Authors:** Bernadette Basilico, Laura Ferrucci, Azka Khan, Silvia Di Angelantonio, Davide Ragozzino, Ingrid Reverte

**Affiliations:** ^1^Institute of Science and Technology Austria (ISTA), Klosterneuburg, Austria; ^2^Department of Physiology and Pharmacology, Sapienza University of Rome, Rome, Italy; ^3^Center for Life Nano- and Neuro-Science, Istituto Italiano di Tecnologia, Rome, Italy; ^4^Laboratory Affiliated to Institute Pasteur Italia – Fondazione Cenci Bolognetti, Department of Physiology and Pharmacology, Sapienza University of Rome, Rome, Italy; ^5^Santa Lucia Foundation (IRCCS Fondazione Santa Lucia), Rome, Italy

**Keywords:** microglia, microglia depletion, synaptic plasticity, PLX, dendritic spines, glutamatergic transmission, learning and memory, behavior

## Abstract

Microglia are dynamic cells, constantly surveying their surroundings and interacting with neurons and synapses. Indeed, a wealth of knowledge has revealed a critical role of microglia in modulating synaptic transmission and plasticity in the developing brain. In the past decade, novel pharmacological and genetic strategies have allowed the acute removal of microglia, opening the possibility to explore and understand the role of microglia also in the adult brain. In this review, we summarized and discussed the contribution of microglia depletion strategies to the current understanding of the role of microglia on synaptic function, learning and memory, and behavior both in physiological and pathological conditions. We first described the available microglia depletion methods highlighting their main strengths and weaknesses. We then reviewed the impact of microglia depletion on structural and functional synaptic plasticity. Next, we focused our analysis on the effects of microglia depletion on behavior, including general locomotor activity, sensory perception, motor function, sociability, learning and memory both in healthy animals and animal models of disease. Finally, we integrated the findings from the reviewed studies and discussed the emerging roles of microglia on the maintenance of synaptic function, learning, memory strength and forgetfulness, and the implications of microglia depletion in models of brain disease.

## Introduction

Microglia are the resident immune cells of the brain (Kreutzberg, 1996; Tremblay et al., 2011). They are derived from primitive myeloid progenitors which colonize the neuroepithelium during the early embryonic development (Ginhoux and Prinz, 2015). Since their discovery by Río-Hortega in 1919, the progress in bioengineering and pharmacology have been pivotal to investigate microglia functions. The 21st century has been the golden age of microglia research thanks to the development of selective ligands and antibodies along with transgenic mouse lines that allowed the visualization and manipulation of microglia (Sierra et al., 2019). These approaches revealed a critical role of microglia in response to infection and injury (Herz et al., 2017) and an active role in brain development and adult physiology (Tremblay et al., 2011). Many research reports are indeed focused on the role of microglia in shaping synaptic plasticity, sculpting brain circuits and in doing so modulating learning and memory processes and behavior (Tremblay and Majewska, 2011; Miyamoto et al., 2013; Morris et al., 2013; Tay et al., 2017; Taylor et al., 2017; Andoh and Koyama, 2021; Cornell et al., 2022).

Overall, the goal of this review is to gain new insights into the causal role of microglia in modulating neural plasticity, learning and memory and behavior *via* loss-of-function strategies.

We first described the methods that have been used to deplete microglia and their limitations (section 2), which are critical to interpret the available data regarding microglia function. Second, we described the effect of depleting microglia on structural and functional synaptic plasticity in the adult brain (section 3). Third, we summarized the effects of microglia depletion on learning and memory and behavior (section 4).

Our review does not cover the signaling pathways involved in the interaction between microglia and neurons, nor the molecular mediators of microglia responses and the various effectors released by microglia to modulate synaptic function or the role of microglia in regulating plasticity during brain development. We refer readers to excellent reviews on these topics (Miyamoto et al., 2013; Schafer et al., 2013; Frost and Schafer, 2016; Neniskyte and Gross, 2017; Werneburg et al., 2017; Marinelli et al., 2019; Cserép et al., 2021).

## Models of microglia depletion

**FIGURE 1:**
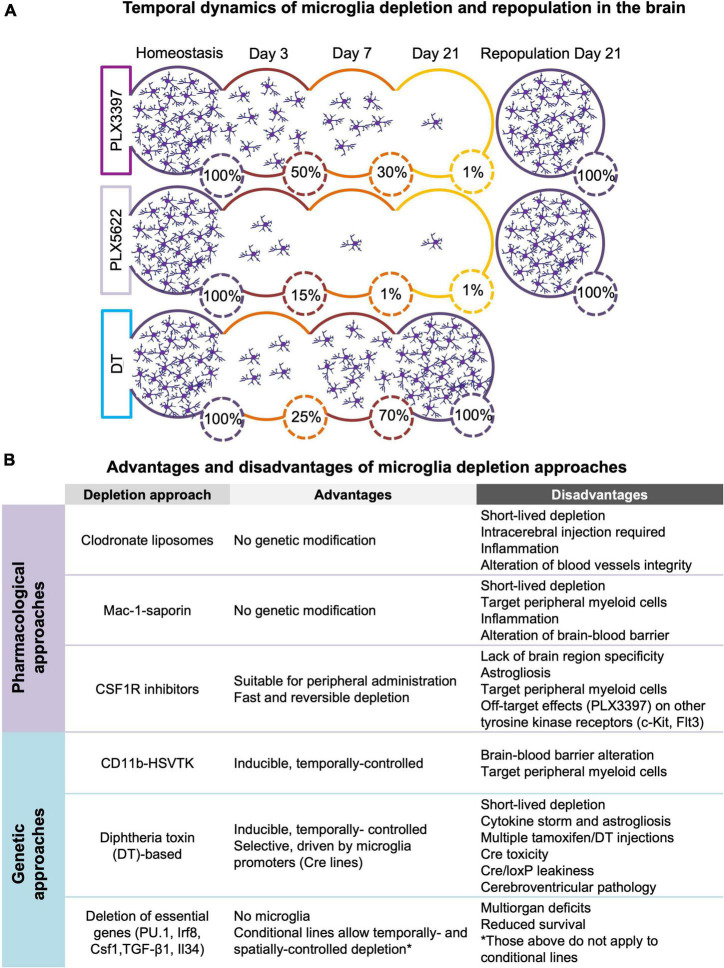
**(A)** Temporal dynamics of microglia depletion and repopulation. Time-course of microglia depletion during administration of CSF1R inhibitors (PLX3397 and PLX5622) and repopulation upon treatment withdrawal, and time-course of genetic microglia depletion and repopulation induced by diphtheria toxin (DT) administration. Represented percentages of microglia in the brain have been extrapolated from published data (Elmore et al., 2014, 2018; Bruttger et al., 2015; Spangenberg et al., 2019; Basilico et al., 2021). **(B)** Advantages and disadvantages of microglia depletion methods. Summary of the main advantages and disadvantages of the different approaches used to deplete microglia (see section 2 for more detail).

The possibility of acute depletion of microglia in an adult animal has contributed to highlight the role of these cells as important mediators of normal brain functioning. Here, we summarized the genetic (section 2.1) and pharmacological (section 2.2) approaches used for microglia depletion *in vivo* along with their respective advantages and technical limitations. Furthermore, we briefly described the impact of different depletion approaches on other central nervous system (CNS)-associated macrophages (section 2.3). Finally, we discussed the process of microglia repopulation after their depletion (section 2.4) and briefly introduced new chemo-genetic approaches to manipulate microglia function (section 2.5) Refer to Figure 1 for a comprehensive overview of the section.

### Genetic approaches for microglia depletion

The genetic approaches for microglia depletion are based on two strategies: the expression of ‘suicidal genes’ under the control of specific microglial promoters (section 2.1.1) and the genetic manipulation of the factors essential for microglia survival (section 2.1.2).

#### Expression of ‘suicidal genes’ (herpes simplex virus thymidine kinase or diphtheria toxin receptor)

The first approach used for depleting microglia genetically was the generation of CD11b-HSVTK transgenic mice (Heppner et al., 2005). These mice express the suicide herpes simplex virus thymidine kinase (HSV-1 TK) gene under the control of the myeloid promoter Cd11b. The HSV-1 TK exerts its toxic activity after the treatment with antiviral drugs like ganciclovir, which are further processed into toxic triphosphates by cell-intrinsic kinases resulting in apoptosis (Heppner et al., 2005). A main limitation of this approach is that the systemic and chronic administration of these antivirals can disturb the peripheral myeloid population. To avoid this limitation, these drugs must be delivered *via* intracerebroventricular injections (Bennett and Brody, 2014).

An alternative approach consists in the use of diphtheria toxin (DT)-based transgenic mice (Parkhurst et al., 2013; Bruttger et al., 2015). Mice carrying the DT or its receptor diphtheria toxin receptor (DTR), downstream of loxP-flanked STOP sequences, are crossed with myeloid promoter-driven Cre recombinase lines, exploiting the exclusive microglia expression of CX3CL1 receptor (CX3CR1) in the CNS (i.e., *Cx3cr1*^Cre^*, Cx3cr1*^CreER^**) (Sauer and Henderson, 1988; Nagy, 2000). Microglia are then depleted upon DT administration, which inhibits intracellular protein synthesis leading to acute cell death (Bruttger et al., 2015). The use of inducible lines such as the *Cx3cr1^CreER^* has the advantage of temporally controlling both the Cre-mediated recombination and the cell ablation with DT, allowing to specifically target microglia over the other myeloid populations (Parkhurst et al., 2013). The limitations of the DT/DTR models are the astrocyte activation and the increase in cytokine production, likely released by reactive astrocytes (Bruttger et al., 2015). Other limitations include their dependence on Cre recombinase which might be toxic (Schmidt et al., 2000; Schmidt-Supprian and Rajewsky, 2007) and the use of tamoxifen (for inducible Cre lines) that binds to macrophages and other estrogen receptor-expressing cells (Donocoff et al., 2020; Green et al., 2020). Recently, some studies raised additional concerns regarding the inducible genetic models targeting microglia. The first is the potential ‘leakiness’ of the Cre/LoxP system into other brain cells or the occurrence of recombination in microglia in absence of tamoxifen induction (Chappell-Maor et al., 2020). Because leakiness varies among the mouse lines created to target microglia, the proper characterization of these Cre lines is necessary (Zhao et al., 2019; Stifter and Greter, 2020). Another concern is the cerebroventricular pathology observed in some of the transgenic lines that seems to be inherent to the ROSA-26iDTR (iDTR) allele after DT treatment, independently of Cre expression (Bedolla et al., 2022). The interpretation of the data obtained using these transgenic lines should be taken with caution. For instance, neuronal cell death (Rubino et al., 2018) and synapse degeneration (Wang et al., 2016) have been reported in these mouse lines, but not with pharmacological depletion approaches. Further validation of these genetic models is needed to improve the confidence of the findings deriving from their application.

#### Deletion of essential factors for microglia maturation and survival

This genetic strategy relies on transgenic mice carrying null mutations in genes critical for microglia development such as the myeloid-specific transcription factor PU.1, interferon-regulatory factor 8 (*Irf8*), colony stimulating factor 1 (*Csf1*) and transforming growth factor beta 1 (*TGF-*β*1*). However, these constitutive models are not suitable for long-term studies as the lack of these essential genes in myeloid cells leads to reduced survival and multiorgan deficits (Shull et al., 1992; Ginhoux et al., 2010; Butovsky et al., 2013; Kierdorf et al., 2013).

To overcome this limitation, Badimon et al. (2020) recently developed a model of inducible ablation of the interleukin-34 (*Il34*) specifically in neuronal progenitor cells (Nestin*^Cre^*) that resulted in the loss of neuron-associated microglia in striatal gray matter and a model of inducible ablation of *Csf1* that led to the loss of microglia in the striatal white matter. This method can also be used to target microglia subpopulations surrounding specific neuronal subtypes by ablating *Il34* under different neuronal promoters (i.e., D1 and D2 receptor-expressing neurons).

### Pharmacological strategies for microglia depletion

Pharmacological strategies for microglia depletion are also available and have potential for the clinical practice. The main pharmacological strategies are treatments with toxic-clodronate (section 2.2.1), antibodies against Mac-1-saporin (section 2.2.2) and CSFR1 inhibitors (section 2.2.3).

#### Toxic-clodronate-containing liposomes

The clodronate-containing liposomes are a toxin-based method to deplete macrophages and microglia that takes advantage of cellular phagocytosis (Van Rooijen and Sanders, 1996). Liposomes, small artificial vesicles, are normally phagocyted by microglia (and macrophages) leading to clodronate accumulation and ultimately resulting in apoptosis (Van Rooijen et al., 1997). However, since liposomes do not cross the bran-blood-barrier (BBB) they must be infused directly into the brain region of interest (Andreou et al., 2017; Han et al., 2019). The main limitation of this approach is that it may induce an inflammatory state and alteration of the blood vessels integrity (Han et al., 2019).

#### Mac-1-saporin

The immunotoxin Mac-1-saporin is an antibody against the macrophage antigen complex-1 (Mac-1), a β2-integrin glycoprotein expressed on the cell surface of most myeloid cells including microglia, conjugated to the ribosome-inactivating protein saporin (Dommergues et al., 2003; Montero et al., 2009). Intracerebroventricular or intrathecal Mac-1-saporin injection induces a transient microglia depletion followed by a rapid repopulation in rodents (Heldmann et al., 2011; Yao et al., 2016). The main limitations of this approach rely on several side effects like inflammatory reactions and BBB breakdown (Yao et al., 2016).

#### Inhibition of colony stimulating factor 1 receptor signaling pathway

A popular pharmacological depletion strategy consists in the inhibition of the colony stimulating factor 1 receptor (CSFR1), essential for macrophages and microglia proliferation, differentiation and survival (Stanley et al., 1997). Few inhibitors have been developed so far: PLX3397, PLX647 (Elmore et al., 2014), the more selective compounds PLX5622 (Spangenberg et al., 2019) and Ki20227 (Xie et al., 2020; Jiang et al., 2022). The most used compounds, PLX3397 and PLX5622 can cross the BBB and are usually formulated in the rodent chow for chronic administration. Many studies demonstrated a relatively fast (over 3 days) and efficient reversible depletion of microglia in the CNS (Elmore et al., 2014; Spangenberg et al., 2019; Green et al., 2020).

The main limitation of using CSF1R inhibitors (CSF1R-i) through diet is the lack of a brain region specificity. A second limitation of CSF1R-i are their putative peripheral effects (Green et al., 2020; Han et al., 2020; Lei et al., 2020, 2021; Green and Hume, 2021) since CSF1Rs are expressed on other myeloid cells (Stanley et al., 1997). Moreover, PLX3397 also binds other tyrosine kinase receptors (i.e., c-Kit and Flt3) and, at high doses, it decreases circulating monocytes in both humans and rodents (Tap et al., 2015; Butowski et al., 2016; Shi Y. et al., 2019). Notably, recent studies showed that PLX5622 reduces a population of circulating Ly6C*^low^* monocytes responsible for the surveillance of the blood vessels (Otxoa-de-Amezaga et al., 2019) and affects peripheral myeloid and lymphoid cell populations, causing irreversible long-term changes in the bone marrow, spleen and blood (Lei et al., 2020). Furthermore, in the context of viral encephalitis, the reduction of neuroinflammation after PLX5622 treatment is not only dependent on microglia depletion but also on the reduced proliferation of mature monocytes (Ly6C*^high^*) in the bone marrow (Spiteri et al., 2022). On the other hand, some studies did not observe any effects in circulating monocytes and tissue macrophages when PLX3397 or PLX5622 was used at the doses typically formulated in the rodent chow (Elmore et al., 2014; Spangenberg et al., 2019; Green et al., 2020).

It must be noticed that CSF1R-i treatment has been reported to affect other brain cells. Indeed, treatment with CSF1R-i decreases oligodendrocyte precursor cells (OPC) gene expression and number, without producing major effects in the number of mature oligodendrocytes and myelination (Hagemeyer et al., 2017; Green et al., 2018; Janova et al., 2018; Garcia-Agudo et al., 2019; Liu et al., 2019). Furthermore, CSF1R-i increase mRNA and protein levels of astrocytic markers (i.e., GFAP) (Elmore et al., 2015; Basilico et al., 2021), with no changes in cell number or morphology (Elmore et al., 2014; Ma et al., 2020; Liu et al., 2021). Notably, GFAP alterations are maintained upon microglia repopulation when treatment is discontinued (Basilico et al., 2021). Recently, it has been reported that CSF1R-i treatment weakens astrocyte gap junctional coupling (Du et al., 2022). Importantly, alterations in astrocytes have also been reported with genetic microglia depletion models (Bruttger et al., 2015; De Luca et al., 2020).

While CSF1R inhibition does not induce gross changes in brain volume, neuronal gene expression (Elmore et al., 2014), neuronal density (Elmore et al., 2018), and intrinsic neuronal membrane properties (Liu et al., 2021), it may cause the accumulation of apoptotic cells in brain regions with high neuronal turnover such as the cerebellum, the olfactory bulb and the dentate gyrus (Ayata et al., 2018).

Despite all the aforementioned caveats, CSF1R-i have been widely employed for microglia depletion due to their ease-of-use in preclinical studies.

### Effect of microglia depletion strategies on other central nervous system-associated macrophages

Central nervous system immune surveillance is not only exerted by microglia but also by other CNS-associated macrophages located in the meninges, the perivascular space, and the choroid plexus that modulate the immune response at the CNS borders. As mentioned above (sections 2.2.1 and 2.2.2), the clodronate-containing liposomes and Mac-1-saporin strategies were developed to target both microglia and macrophages, rendering these tools not specific solely for the microglia population. CNS-associated macrophages, like microglia, are dependent on the CSF1/CSF1R axis for their survival and proliferation and might be affected by CSF1R-i. Specifically, a sustained treatment (21 days) with PLX3397 fully depletes meningeal and choroidal CNS-associated macrophages in healthy mice (Van Hove et al., 2019). In the context of pathology, in a murine model of viral encephalitis, treatment with PLX5622 also depletes a subpopulation of CNS-associated macrophages (Spiteri et al., 2022). Moreover, microglia and CNS-associated macrophages also share other myeloid markers such as CD11b, Iba-1, and CX3CR1, whose promoters have been used for genetic microglia targeting, and therefore commonly used genetic microglia depletion strategies fail to distinguish between microglia and CNS-associated macrophages (Goldmann et al., 2016; Van Hove et al., 2019; Prinz et al., 2021).

### Microglia repopulation

Microglia cells display an extraordinary capacity of regeneration and repopulation in the brain tissue even after depletion protocols able to deplete microglia up to 90% (Elmore et al., 2014; Huang et al., 2018; Zhan et al., 2019). Although the origin of repopulating microglia is still matter of debate (section 2.4.1), acute microglia depletion and subsequent repopulation have been suggested as a therapeutic strategy to re-establish homeostasis in the diseased brain (section 2.4.2).

#### The origin of repopulating microglia

In the context of pharmacological depletion strategies, Elmore et al. (2014) first proposed that repopulation (3 weeks PLX3397 + 3 weeks withdrawal) occurred from brain Nestin+ neuroectodermal progenitors, *de facto* excluding the proliferation from surviving or infiltrating cells as primary source for the new-born microglia. However, this hypothesis has been questioned on two grounds: (1) the origin of microglia from the yolk sac during development and (2) lack of evidence supporting the presence of microglia progenitors in the adult brain (Ginhoux and Prinz, 2015). Huang et al. (2018) were the first to refute the Nestin+ progenitor hypothesis demonstrating, through fate-mapping approach that the repopulation depends on the proliferation of residual microglia. The authors did not observe any fluorescently labeled microglia in mice with permanently labeled Nestin+ cells and their progeny (Nestin-CreER^*T*2^:Ai14 mice) after treatment (2 weeks PLX5622 + 2 weeks of withdrawal). These findings speak against a possible Nestin+ progenitor. On the contrary, they observed that repopulation occurred by proliferation of the surviving microglia cells in mice expressing fluorescently labeled microglia (Cx3cr1-CreER:Ai14 mice). Importantly, Weber et al. (2019) obtained similar results after treatment (2 weeks PLX5622 + 3 weeks of withdrawal) in Cx3cr1^*CreER–YFP*/+^R26*^tdTOM^* mice.

Even in the context of genetic microglia depletion, the origin of microglia repopulation is still under debate. Furthermore, recent evidence suggests that it is also largely dependent on the strategy being used. By using Cx3cr1^*CreER*/+^R26^*iDTR*/+^ mice, Bruttger et al. (2015) observed a robust repopulation sustained by the self-maintenance of tissue-resident macrophage populations without contribution of external progenitors. Conversely, Lund et al. (2018) observed a competitive repopulation process leading to a mosaic of cells derived from both proliferating microglia and brain infiltrating Ly6C*^hi^* monocytes in Cx3cr1^*CreER*–YFP/+^R26*^DTA/+^* mice. The monocyte-derived cells retained transcriptional and functional differences from resident microglia, being more proinflammatory even after long-term integration into the CNS. More recently, Zhou et al. (2022) identified a subpopulation of microglia exhibiting high CX3CR1 expression and devoid of the genetic modification in Cx3cr1^*CreER*–YFP/+^R26*^DTA/+^* mice. This subpopulation, less than 1% of all the microglia under homeostatic conditions, eluded the depletion and proliferated extensively, representing a big portion of the repopulated microglia pool.

To our knowledge, little is known about the molecular signaling that modulates and drives microglia repopulation in the brain. Bruttger et al. (2015) hypothesized the involvement of the interleukin-1 receptor signaling, showing that the administration of an IL1R antagonist impaired repopulation by affecting the proliferation of microglia clusters. Another potential candidate is the CX3CL1/CX3CR1 pathway, whose signaling exerts a positive effect on microglial repopulation (Zhan et al., 2019; Zhou et al., 2022).

#### The therapeutic potential of repopulating microglia

Microglia repopulation after an acute depletion has been proposed as a promising therapeutic strategy to ‘reset’ microglia to a homeostatic phenotype in brain disease conditions (Cartier et al., 2014; Butowski et al., 2016).

In the healthy adult brain repopulated microglia after PLX3397 withdrawal display similar inflammatory transcriptomic profiles and responsiveness to pro-inflammatory stimuli to resident microglia (Elmore et al., 2015). On the other hand, microglia repopulation in the aged brain ‘reverses’ age-related microglia morphological changes, alterations in neuronal gene expression and deficits in hippocampal long-term potentiation, resulting in an improvement of cognitive functions (Elmore et al., 2018). Similarly, after brain injury microglia repopulation promotes brain recovery (Rice et al., 2017) and improves cognition (Henry et al., 2020; Willis et al., 2020). It has also been suggested that homeostatic genetic signatures displayed by the repopulated microglia might prevent its switching to a disease-associated microglia phenotype, essential to limit Aβ plaque–mediated tau pathogenesis in Alzheimer’s disease (AD) (Casali et al., 2020; Gratuze et al., 2021).

However, in some models of brain pathology microglia repopulation has been found to be ineffective (stress; Lehmann et al., 2019; Weber et al., 2019) or even detrimental [autoimmune encephalitis (EAE)]; (Rubino et al., 2018). The effects of microglia repopulation on behavioral and cognitive functions in brain disease models are described in section 4.3.

### Microglia manipulation through a chemogenetic approach

Recently, a new strategy based on the Gq- and Gi-coupled Designer Receptor Exclusively activated by a Designer Drug-(DREADD) technology has been developed which enables the selective stimulation or inhibition of microglia (Armbruster et al., 2007; Roth, 2016).

The DREADD technology is based on the control of engineered G protein-coupled receptors-mediated signaling. Upon activation, these receptors undergo structural rearrangements facilitating the interaction with a family of canonical heterotrimeric G-proteins (e.g., Gq, Gs, Gi, and G12/13) leading to activation of intracellular signaling pathways. The engineered receptors can be activated by the pharmacologically inert compound clozapine-N-oxide (CNO) which leads to different modulations of microglia function based on the type of G-protein associated to them (Armbruster et al., 2007; Zhu et al., 2016).

Recently, DREADDs developed under the CD68 (Grace et al., 2016, 2018) and Cx3cr1 (Saika et al., 2020; Klawonn et al., 2021; Yi et al., 2021) microglia/macrophage promoters have allowed for the manipulation of microglia. Most of these studies applied the DREADD technology to manipulate microglia function in the context of pain research. The authors found that microglia activation *via* Gq DREADDs induce allodynia (see Box 1) (Grace et al., 2018; Saika et al., 2020), whereas microglia inhibition *via* Gi DREADDs attenuate neuropathic pain and associated neuroinflammation (Yi et al., 2021). Recently, Bolton et al. (2022) also used a Gq-DREADDS under the Cx3cr1 promoter to activate microglia in a model of early life adversity and found that this manipulation normalized microglia process dynamics, synapse density and adult hormonal and behavioral stress responses. Notably, the mechanisms and effects of Gi- or Gq-DREADD on microglia intracellular signaling are still poorly understood (Grace et al., 2018; Saika et al., 2020).

**BOX 1 Behavior definitions and behavioral procedure descriptions.**

*Spontaneous locomotor activity* is typically assessed to exclude gross sensorimotor effects of pharmacological compounds or genetic alterations. It is typically evaluated in unconditioned response tests based on the exposure of rodents to novel environments. These tests also evaluate *“anxiety-like” behavioral responses* of rodents to unfamiliar aversive places. The *open field test (OF), elevated-plus maze (EPM)*, and *light/dark* tests are based on ethological behavior that harnesses the conflict between the innate exploratory behavior of rodents in response to mild stressors (i.e., novel environments) and their aversion to open, elevated and brightly illuminated spaces (Belzung and Griebel, 2001; Bourin and Hascoët, 2003; Walf and Frye, 2007; Seibenhener and Wooten, 2015).

*Repetitive behaviors*: innate behaviors displayed by rodents, such as *marble burying* and *self-grooming*, might become repetitive/perseverative, like observed in rodent models of psychiatric illness or neurodevelopmental disorders. Repetitive behavior might be sensitive to anxiolytic and antidepressant drugs (Thomas et al., 2009; Ahmari, 2016).

*Sensory perception*, which includes vision, audition, olfaction, gustation and somatosensation, informs the brain about the environment and influences how the animal responds to it (Burn, 2008).

*Nociceptive tests* measure the latency or the threshold of the paw or tail withdrawal reflex after the application of electrical, thermal, mechanical, or chemical noxious stimuli. *Mechanical allodynia* is the response to a mechanical non-nociceptive stimulus, which is typically incremented in rodent models of chronic pain, while *thermal hyperalgesia* refers to an increased pain sensitivity due to an altered temperature perception (Barrot, 2012; Inoue and Tsuda, 2018). Nociception differs from pain perception, the latter including sensory, cognitive, and emotional aspects associated with damage (Barrot, 2012).

*Sensory-motor tests*, such as Gait Test, Grip Strength, Beam Walk, Bar Test, Hurdle Test and Pole Test, are typically used to assess gross motor deficits in mice after stroke or injury (Balkaya et al., 2013). The *Rotarod Test* is widely used to evaluate balance and motor coordination in mice. Standard protocols use a maximum acceleration of 40 or 60 r.p.m., with schedules ranging from 3 to 9 trials over 1 to 3 days. Performance in the rotarod improves with training allowing the evaluation of motor skill learning. Parkhurst et al. (2013) used a protocol consisting of an acceleration from 0 to 100 r.p.m. and a schedule of 60 trials/day over 3 consecutive days that allowed them to evaluate motor learning (Shiotsuki et al., 2010; Parkhurst et al., 2013).

*Spontaneous alternation* in the *Y- or T-shaped maze* can be interpreted as a measure of spatial *working memory*. When allowed to freely explore the apparatus, mice with an intact cognitive capacity typically remember the previously visited arms and show a tendency to enter a less visited arm (Kraeuter et al., 2019).

*Sociability*, the innate tendency of an animal to engage in social investigation is typically assessed in the *Three-Chamber Test* (also known as *Crawley’s test)* in rodents. It measures the preference to investigate a conspecific (located under a wire cup in one of the compartments) vs. an inanimate object. A lack of preference indicates a deficit in pro-social behavior or social interaction. In a subsequent phase of the test, the propensity of rodents to investigate a novel conspecific more thoroughly than the previous encountered one (familiar) is measured. A lack of preference for the novel mouse indicates either social novelty avoidance or poor *social recognition memory*. C57BL/6 or 6J mice (typical experimental strains) also display sociability toward mice of other strains (e.g., CD-1 mice) (Crawley, 2007; van der Kooij and Sandi, 2012; Ayash et al., 2020).

*Recognition learning and memory*, the ability to discriminate a previously encountered item as familiar, is typically evaluated with the *Novel Object Recognition (NOR)*. The test exploits the fact that mice display an innate tendency to spend more time investigating a never encountered (novel) object than a familiar one. A variant of this task is the *Novel Object Location (NOL)*, in which only the location of the object in the arena is changed between the familiarization and test phases. A third variant is the novel *Context-Object-Discrimination (COD)* in which is measured the capacity to discriminate the congruency between the objects and the context where they were previously encountered. The performance of these tasks depends on the integrity of hippocampus, perirhinal, and medial prefrontal cortices circuitry (Broadbent et al., 2009b; Barker and Warburton, 2015).

*Fear conditioning* is a type of *associative learning* achieved by a Pavlovian/Classical conditioning procedure in which a neutral conditioned stimulus (CS), typically an auditory cue, is associated to an aversive unconditioned stimulus (US), often a mild electric foot-shock. In presence of the CS rodents express freezing behavior, a species-specific fear response, indicative of the memory of the auditory cue with the aversive stimulus (*Auditory or Cued Fear Conditioning, AFC*). Animals also exhibit fear responses when returned to the chamber in which the tone and shock were paired, or a chamber in which shocks occur alone (*Contextual Fear Conditioning, CxFC*). The hippocampus is required for the formation and retrieval of context–fear associations, whereas the amygdala is required for conditioning and recall of associations to both contextual and discrete cues (LeDoux, 2003).

*Spatial learning* refers to the process by which an animal makes a mental representation of the surrounding environment that permits its orientation and location of relevant places. Among the most used paradigms are the *Morris Water Maze (MWM)* and the *Barnes Maze (BM)*. Both tests use the innate motivation of the animal to find a safe location, a submerged platform in the case of the MWM, and a hidden hole in the case of the dry BM. The animal must rely in distal cues to learn to navigate the maze and find the target. These tests evaluate hippocampally dependent spatial learning and reference memory (Vorhees and Williams, 2006; Gawel et al., 2019).

*‘Depressive-like’ behaviors* in rodents typically refer to symptoms of helplessness, anhedonia, and behavioral despair since the full spectrum of depressive symptoms cannot be recapitulated in animal models. The expression of despair can be evaluated by measuring immobility in the *Forced Swim Test (FST)* and *Tail Suspension Test (TST)*, which place the animal in inescapable distressful situations. The *learned helplessness model* measures the rate of non-escape responses displayed by rodents following an uncontrollable and inescapable stress when re-exposed to the same situation. A decrease in *sucrose preference* is a measure of anhedonia in rodents. All these measures are sensitive to the action of antidepressants (Krishnan and Nestler, 2011).

## Effects of microglia depletion on synaptic function and plasticity

**FIGURE 2:**
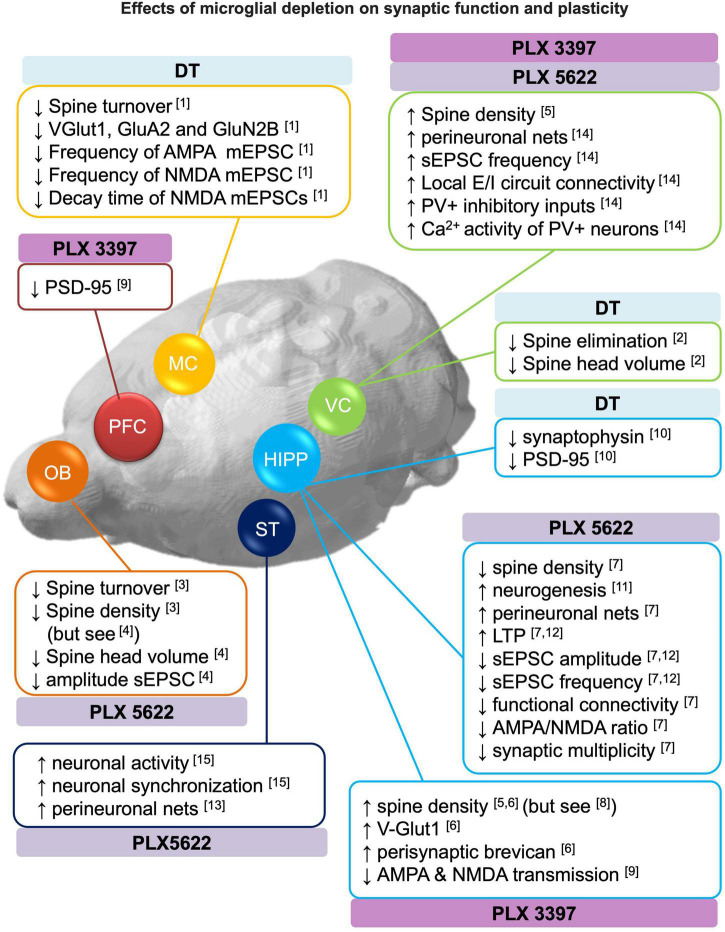
Effects of microglial depletion on synaptic function and plasticity. Schematic representation of the reported effects of microglia depletion with CSF1R inhibitors (PLX3397 and PLX5622) and genetic DT-induced on dendritic spines, neurogenesis, perineuronal nets, excitatory and inhibitory (E/I) neurotransmission (see section 3 for more detail). DT: diphtheria toxin, HIPP: hippocampus, MC: motor cortex, OB: olfactory bulbs, PFC: prefrontal cortex, ST: striatum, VC: visual cortex. [1] Parkhurst et al., 2013; [2] Ma et al., 2020; [3] Reshef et al., 2014; [4] Wallace et al., 2020; [5] Rice et al., 2015; [6] Strackeljan et al., 2021; [7] Basilico et al., 2021; [8] Pinto et al., 2020; [9] Yegla et al., 2021; [10] De Luca et al., 2020; [11] Elmore et al., 2018; [12] Corsi et al., 2022; [13] Crapser et al., 2020; [14] Liu et al., 2021; [15] Badimon et al., 2020.

Over the last decades, increasing evidence revealed that microglia are involved in the regulation of synaptic plasticity under physiological conditions. Microglia contact axonal boutons and dendritic shafts in an activity-dependent manner resulting in the elimination and/or formation of spines and pre-synaptic elements (Wake et al., 2009; Tremblay et al., 2010; Paolicelli et al., 2011; Schafer et al., 2012; Hayashi et al., 2013; Lim et al., 2013; Miyamoto et al., 2016; Sipe et al., 2016; Weinhard et al., 2018; Basilico et al., 2019; Wang and Huang, 2020). In this section, we reported the effects of microglia depletion on structural plasticity (section 3.1), neurogenesis (section 3.2) and functional changes in synaptic transmission (section 3.3) in the adult brain. Refer to Figure 2 for a comprehensive overview of the section.

### Structural plasticity

Synaptic contacts are structurally plastic, and connectivity is remodeled through mechanisms of synapse formation, stabilization and elimination. Although structural plasticity is maximal during brain development, in the adult brain it is important for learning and memory and to restore function after brain damage (Xu et al., 2009; Caroni et al., 2012). In this section, we reported the effects of microglia depletion on the formation, elimination and/or stabilization of dendritic spines, and the effects of microglia depletion in perineuronal nets.

Perineuronal nets (PNN) are components of the extracellular matrix that modulate local inhibitory transmission and plasticity in the adult brain. PNNs have been related to the stabilization of synaptic connections, playing a role in synaptic maturation and the formation and consolidation of memories (Gogolla et al., 2009). Alterations in PNNs are also present in diseases where memory deficits are core features (Carulli and Verhaagen, 2021). Pharmacological microglia depletion has been reported to increase density and intensity of perineuronal nets in several brain regions (Crapser et al., 2020; Basilico et al., 2021; Liu et al., 2021) and to increase the expression of peri-synaptic extracellular matrix proteoglycan brevican (Strackeljan et al., 2021). These results are in line with recent evidence reporting that microglia engulf components of the extracellular matrix in response to neuronal cytokine signaling (Nguyen et al., 2020). The functional implications of the role of microglia on degrading PNN might vary depending on age, brain-region and or inflammatory conditions. For instance, Tansley et al. (2022) showed that after nerve injury, degradation of PNNs by microglia enhances the activity of projection neurons contributing to pain-related behaviors.

Concerning the dynamics of dendritic spines, three studies on microglia depletion (both genetic and pharmacological) consistently reported a reduced spine turnover in the motor cortex (Parkhurst et al., 2013), visual cortex (Ma et al., 2020) and granule cells (abGC) of the olfactory bulb (Reshef et al., 2014) as revealed by *in vivo* two-photon imaging.

On the other hand, the analysis of spine density after microglia depletion provided heterogeneous results. In particular, spine densities were increased in: visual cortex [DT genetically induced microglia depletion (Ma et al., 2020); PLX3397 (4 weeks) (Rice et al., 2015)], olfactory bulb-abGCs [PLX5622 (4 weeks) (Wallace et al., 2020)] and hippocampal CA1 [PLX3397 (4 weeks) (Rice et al., 2015; Strackeljan et al., 2021)]. Spine densities were decreased in: olfactory bulb abGCs [PLX5622 (4 weeks) (Reshef et al., 2014)] and hippocampal CA1 [PLX5622 (1 week) (Basilico et al., 2021)]. No effect was observed in the hippocampus of juvenile mice (P22) [PLX3397 (1 week) (Pinto et al., 2020)]. These conflicting findings outlined above can be recapitulated in the context of spine turnover. Indeed, it has been suggested that in homeostatic conditions spine turnover is very dynamic and rarely impacts long-term network structure (Berry and Nedivi, 2017). It is therefore plausible that the net spine density is an insufficient measure to capture the effects of microglia depletion on spines. Furthermore, the method or duration of microglia depletion may also contribute to the variability observed on spine density measures.

Spine morphology may be indicative of the stabilization and maturation of spines, correlates with synaptic strength and has been implicated in memory consolidation processes (Berry and Nedivi, 2017). Some studies suggest that microglia depletion diminish the rate of mature spines, revealed by an overall reduction in spine head size (Ma et al., 2020; Wallace et al., 2020), an increase of immature thin or filopodia-like spines (Rice et al., 2015; Wallace et al., 2020) or a reduction in mature spines (mushroom, stubby, and bifurcated) (Rice et al., 2015; De Luca et al., 2020; Ma et al., 2020). On the other hand, three studies reported no effects of microglia depletion on spine morphology (Reshef et al., 2014; Basilico et al., 2021; Strackeljan et al., 2021). As discussed above the discrepant results are likely due to methodological differences between studies.

The impact of microglia on dendritic spine turnover and density is not easy to define, since the dynamics of persistent and transient spines have different impacts on the local circuit (Berry and Nedivi, 2017), and, as discussed above for PNNs, it might depend on age, brain-region and or inflammatory conditions. Despite this, two studies highlighted that the role of microglia on spine regulation might be relevant for learning and memory and behavioral performance. In this regard, Parkhurst et al. (2013) observed that reduced spine turnover in the motor cortex was associated with a reduced motor learning rate in mice with depleted microglia. Furthermore, Wang C. et al. (2020) observed that microglia depletion increased spine density in hippocampal engram cells whose reactivation correlated with the recall of a contextual fear memory (see section 4.1.7).

Altogether, these data suggest that microglia actively participate in circuit remodeling by modulating spine turnover and PNN degradation.

### Neurogenesis

Neurogenesis, a process of generation of new neurons, represents another form of brain structural plasticity which occurs in two regions of the adult brain, i.e., olfactory bulb and the dentate gyrus of the hippocampus, by integrating newly born neurons into already existing circuits (Van Praag et al., 2002; Lledo et al., 2006; Rodríguez-Iglesias et al., 2019).

It has been reported that microglia depletion with PLX5622 increased the number of dividing neurons [bromodeoxyuridine (BrDU+)] in the dentate gyrus (Elmore et al., 2018). Notably, Wang C. et al. (2020) reported that microglia depletion (PLX3397 5 weeks) prevented memory forgetting induced by the pro-neurogenic drug memantine, suggesting a role of microglia in hippocampal neurogenesis-related plasticity (see section 4.1.7). However, no effects have been reported on the number of new-born cells [doublecortin (DCX+)] in the olfactory bulb (Reshef et al., 2017). These apparent conflicting findings are in line with Reshef et al. (2014) who reported that microglia-mediated fractalkine signaling was involved in hippocampal- but not olfactory bulb-related neurogenesis, suggesting that the modulation of neurogenesis by microglia is regional specific. Diaz-Aparicio et al. (2020) also showed that microglia balance proliferation and survival in the hippocampal neurogenic niche by secreting neurogenic modulatory factors following the phagocytosis of apoptotic cells.

In a model of traumatic brain injury (TBI), where survival of immature neurons (DCX+) was compromised, microglia depletion (genetic DT-induced and PLX5622) further decreased immature neuron survival. However, upon repopulation microglia promoted immature neuron survival (Willis et al., 2020).

Altogether, the evidence suggest that microglia in the healthy brain positively regulate hippocampal neurogenesis and related plasticity and that its repopulation after injury promote functional recovery by supporting immature neuron survival.

### Functional plasticity

Few *in vivo* imaging studies demonstrated that microglia monitor and respond to neuronal activity (Wake et al., 2009; Tremblay et al., 2010; Pfeiffer et al., 2016). Recent evidence also suggest that microglia can suppress neuronal activity (Badimon et al., 2020) or regulate neuronal synchronization (Akiyoshi et al., 2018). These findings suggest a neuron-microglia bidirectional communication that promotes functional changes in synapses and neuronal circuits. In this section, we summarized the main findings observed on the regulation of excitatory (section 3.3.1) and inhibitory (section 3.3.2) neurotransmission after microglia depletion.

#### Excitatory neurotransmission

Studies employing microglia depletion strategies indicate that microglia regulate the level of synaptic proteins and the functioning of glutamatergic excitatory synapses, likely in a brain region-dependent manner.

In the motor cortex, Parkhurst et al. (2013) observed a decrease in the abundance of the synaptic proteins (i.e., GluN2B, VGlut1, and GluA2) after genetic DT-induced microglia depletion in juvenile mice. These changes were associated with reduced frequencies of miniature excitatory postsynaptic currents (mEPSCs) in layer V pyramidal neurons, suggesting a decrease in spontaneous glutamate release. Moreover, the decay time of NMDA mEPSCs was reduced, consistent with the observed reduction of GluN2B subunits. These synaptic alterations were associated with deficits in learning-induced dendritic spine remodeling and decreased performance in learning tasks dependent on microglia BDNF signaling (refer to section 4).

In the hippocampus, three studies showed that the absence of microglia leads to decreased excitatory synaptic transmission (Basilico et al., 2021; Yegla et al., 2021; Corsi et al., 2022). In particular, Basilico et al. (2021) demonstrated that microglia depletion with PLX5622 (1 week) led to increased long-term potentiation (LTP), reduced glutamatergic transmission and reduced functional connectivity at CA3-CA1 hippocampal synapses. These changes were associated with immature synaptic features such as reduced AMPA/NMDA ratio and impaired multiplicity. Similarly, Corsi et al. (2022) observed increased plasticity and reduced excitatory neurotransmission following PLX5622 (1 week). Both studies highlighted the importance of the CX3CR1/CX3CL1 axis in microglial control of synaptic function (Basilico et al., 2021; Corsi et al., 2022). A third study showed that treatment with PLX3397 (3 weeks) was associated with decreased synaptic transmission in both young and aged rats. In particular, through extracellular field recordings in hippocampus, the authors showed an impairment in both AMPAR- and NMDAR-mediated transmission at CA3-CA1 synapses (Yegla et al., 2021). During microglia repopulation after genetic DT-induced depletion, a reduction of synaptophysin and PSD-95 (but not Vglut1, Glun2A,2B) was observed in the hilus of the hippocampus (De Luca et al., 2020). Decreased amplitude, but not frequency of sEPSC was also observed in abGC neurons of the olfactory bulb devoid of microglia (PLX5622 4 weeks), suggesting weaker excitatory inputs (Wallace et al., 2020).

On the other hand, in layer V pyramidal neurons of the visual cortex, microglia depletion with PLX3397 and PLX5622 (3–4 weeks) in adult mice enhanced spontaneous EPSC (sEPSC) frequency, but not amplitude, suggesting an increase in presynaptic release. The authors also observed an increase in both excitatory and inhibitory synaptic connections to excitatory cortical neurons assessed with a functional circuit mapping experiment (Liu et al., 2021). Similarly, Badimon et al. (2020) showed that microglia depletion with PLX5622 (3 weeks) resulted in increased neuronal activity and synchronization in the mouse striatum revealed by *in vivo* two-photon calcium imaging, suggesting that microglia suppress neuronal activation in this brain circuit. The authors also identified a negative feedback mechanism involving the purinergic signaling. This consisted in the ability of microglia to sense ATP released upon neuronal activation through the purinergic receptor P2RY12. Subsequently, microglia convert ATP in AMP and then adenosine, which negatively modulates activity in striatal D1-expressing neurons acting on adenosine A1AR receptors.

Altogether, these data point to a brain region-dependent role of microglia in regulating excitatory synaptic function. In support of this regional disparity, recent studies reported a phenotypic heterogeneity of microglia between brain regions (Grabert et al., 2016; De Biase et al., 2017; Ayata et al., 2018). Notably, the microglia depletion approaches used in the aforementioned studies (i.e., DT-induced and PLX) lack regional specificity and hence deplete microglia in the whole brain. Consequently, compensatory mechanisms occurring between brain areas cannot be ruled out.

#### Inhibitory neurotransmission

Little is known about the impact of microglia depletion on GABAergic inhibitory neurotransmission. The only study showing GABAergic alterations (Liu et al., 2021) reported that microglia depletion (PLX3397 3 weeks) upregulated parvalbumin (PV) expression and calcium activity of inhibitory PV+ interneurons in the visual cortex, suggesting increased PV+ inhibitory inputs onto L5 pyramidal neurons in the microglia-depleted brains. On the other hand, (Ma et al., 2020) did not observe changes in miniature inhibitory postsynaptic currents (mIPSC) in the L2/3 pyramidal neurons of the visual cortex in juvenile mice treated with PLX3397 (2 weeks). Likewise, microglia depletion with PLX5622 did not induce changes in spontaneous inhibitory postsynaptic currents (sIPSC) in the adult hippocampus (Basilico et al., 2021) or abGC neurons of the olfactory bulb (Wallace et al., 2020).

Like the evidence regarding glutamatergic neurotransmission (section 3.2.1), these data point to a microglia brain region-dependent regulation of inhibitory synaptic function. Likely, microglia exert a fine control of the excitation-inhibition balance depending on local micro-environmental cues.

## Effects of microglia depletion on behavior

For decades, the study of behavior, learning and memory has focused only on neuronal mechanisms. Activity-dependent synaptic plasticity has been proposed to be the substrate of learning and memory (Bliss and Lømo, 1973; Takeuchi et al., 2014; Sweatt, 2016; Humeau and Choquet, 2019). Recent evidence suggested that microglia respond to neuronal activity and modulate synaptic transmission, plasticity and behavior (Yirmiya and Goshen, 2011; Morris et al., 2013; Parkhurst et al., 2013; Tay et al., 2018; Rodríguez-Iglesias et al., 2019; Badimon et al., 2020; Wang C. et al., 2020; Cornell et al., 2022).

In this section, we summarized the recent literature on the impact of microglia depletion on behavior in the healthy adult animal (section 4.1), impact of microglia depletion during development on adult behavior (section 4.2) and microglia depletion and behavior in rodent models of disease (section 4.3).

### Effects of microglia depletion during adulthood on behavior in healthy rodents

**FIGURE 3:**
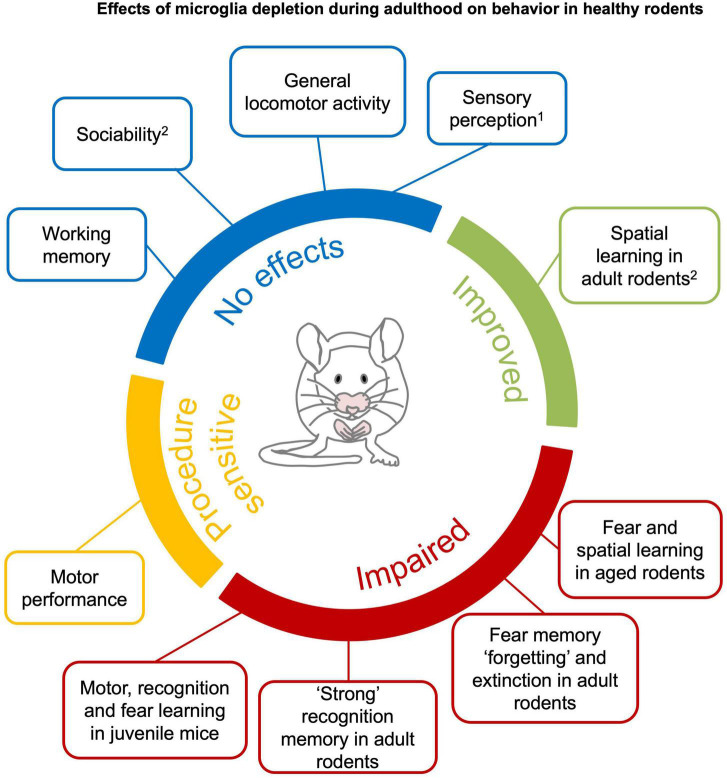
Effects of microglia depletion during adulthood on behavior in healthy rodents. Microglia depletion in the healthy adult brain does not have major effects on sensory perception^1^, general locomotor activity, anxiety, sociability and working memory (*blue*). Motor function is impaired after genetic DT-induced microglia depletion (Parkhurst et al., 2014; Rubino et al., 2018), but not pharmacological depletion (*yellow*). Complex recognition memory and specific learning processes, such as “forgetting” and extinction of fear memories, are compromised in young mice with microglia depletion. Learning deficits are observed when microglia are depleted in juvenile or aged mice (*red*). Spatial learning is improved in young mice (*green*).^1^An exception is reported by Rubino et al. (2018) that found altered gustatory perception in genetic DT-induced microglia depletion. ^2^Clodronate injection in the hippocampus have been reported to impair sociability and spatial learning in young mice (Torres et al., 2016). See section 4.1 and Supplementary Tables 1–8 for detailed information.

In this section, we reviewed the effect of microglia depletion on spontaneous locomotor activity (section 4.1.1), sensory perception (section 4.1.2), motor and performance learning (section 4.1.3), sociability and social recognition memory (section 4.1.4), recognition learning and memory (section 4.1.5), associative learning and memory (section 4.1.6), and spatial learning and memory (section 4.1.7). Refer to Figure 3 for a comprehensive overview of the section.

#### Spontaneous locomotor activity

Spontaneous locomotor activity in a novel environment is typically assessed in preclinical experimental settings to exclude gross sensorimotor effects of pharmacological compounds or genetic alterations (Seibenhener and Wooten, 2015). In general, the literature consistently showed that depletion of microglia does not affect locomotor activity, ‘anxious-like’ or repetitive behaviors (see Box 1), independent of the species (mice or rats), age, method and duration of microglia depletion (Supplementary Table 1) (Elmore et al., 2014, 2018; Dagher et al., 2015; Rice et al., 2015; Torres et al., 2016; Ayata et al., 2018; Adeluyi et al., 2019; De Luca et al., 2019; Lehmann et al., 2019; Spangenberg et al., 2019; Weber et al., 2019; Allen et al., 2020; Badimon et al., 2020; Wang C. et al., 2020; Wu et al., 2020; Xie et al., 2020; Basilico et al., 2021; Cao et al., 2021). Similarly, no effects on locomotor activity have been reported upon microglia repopulation (up to 2–3 weeks after depletion with PLX3397 or clodronate liposomes) (Elmore et al., 2014; Torres et al., 2016). There are two noticeable exceptions, Vichaya et al. (2020) who observed reduced voluntary wheel running in absence of microglia (PLX5622 4 weeks) and Elmore et al. (2018) who observed increased exploration of a novel environment after 4 weeks of microglia repopulation.

#### Sensory perception

Deficits in sensory perception (Box 1) may affect behavioral performance. For this reason, it is critical to exclude any sensory deficit caused by microglia depletion. At present, there are a few studies that addressed sensory competence in microglia depletion models. Particularly, they investigated visual, gustatory, nociceptive and olfactory perception (Supplementary Table 2).

Wang et al. (2016) evaluated whether sustained genetic tamoxifen-induced microglia depletion (4 weeks) influenced the structure and function of the retina in mice. The authors found that visual acuity—measured by elicited motor reflexes in response to light stimuli—was intact, despite they observed degeneration of photoreceptor synapses. Two studies reported that microglia depletion with PLX does not affect nociception (Box 1) in absence of previous lesion or damage (Liang et al., 2019; Sawicki et al., 2019). Badimon et al. (2020) reported that microglia depletion (PLX5622, 3 weeks) did not affect performance in a task that relies on olfaction in mice, suggesting that this sensory modality is intact in absence of microglia.

On the other hand, De Luca et al. (2019) observed that genetic DT-induced microglia depletion in rats reduced food and water intake. By employing an extensive battery of behavioral and neurobiological assays, the authors concluded that the absence of microglia altered the hypothalamic-thalamic gustatory circuitry, resulting in a strong distaste for palatable foods (i.e., saccharin, sucrose, high-fat, and high-sugar diets).

Given the paucity of studies explicitly addressing gustatory perception, to gain a hint on this matter, we reported the results from other studies that monitored food and fluid intake in the absence of microglia. Consistent with the aforementioned study, pointing to an alteration of the gustatory system, chronic microglia depletion in mice with PLX5622 (8 weeks) reduced the consumption of high-fat diet but not standard diet (Valdearcos et al., 2017). On the contrary, shorter PLX3397/PLX5622 treatment (1–5 weeks) did not affect mice consumption of standard diet, high-fat diet, sucrose or alcohol (Gao et al., 2019; Ali et al., 2020; Cai et al., 2020; Wang J. et al., 2020; Cansell et al., 2021; Warden et al., 2021). The available evidence do not allow to draw a clear conclusion and future studies should address these discrepancies, likely arising from diverse microglia depletion methods and durations. Future studies also should take into account that microglia depletion with PLX may alter microbiota composition (Yang et al., 2021) and metabolic parameters (Ali et al., 2020).

#### Motor performance and motor learning

Regarding motor performance (Box 1), genetic but not pharmacological microglia depletion has been associated with motor impairment (Supplementary Table 3).

Pharmacological microglia depletion with CSFR1 inhibitors in mice did not induce general motor deficits in a variety of sensory-motor tests (Qu et al., 2017; Yang et al., 2018; Garcia-Agudo et al., 2019; Crapser et al., 2020; Henry et al., 2020; Li et al., 2020; Xie et al., 2020) and it did not induce alterations on balance and motor coordination as revealed by the rotarod test (Elmore et al., 2014; Qu et al., 2017; Yang et al., 2018; Liang et al., 2019; Badimon et al., 2020; Crapser et al., 2020). Similarly, no effects were observed upon microglia repopulation (Elmore et al., 2015; Henry et al., 2020).

On the contrary, using genetic DT-induced microglia depletion, Parkhurst et al. (2013) observed that juvenile mice displayed reduced rate of motor learning improvement which was associated with a reduced motor learning-induced spine turnover. By using a similar conditional animal model, Rubino et al. (2018) reported that microglia depletion and subsequent repopulation triggered ataxia and motor incoordination. The authors also observed neuronal death in the somatosensory cortex and an inflammatory signature in repopulated microglia.

These conflicting results suggest that the effects of microglia depletion on motor function might be sensitive to the approach used for microglia depletion.

#### Sociability and social recognition memory

Sociability and social recognition memory (see Box 1) are not affected by pharmacological whole-brain microglia depletion (Supplementary Table 4). In particular, mice treated with either PLX3397 or PLX5622 did not display deficits in social preference and recognition memory of conspecifics (Torres et al., 2016; Badimon et al., 2020) nor deficits in the interaction with mice of other strains (Lehmann et al., 2019). Subsequent microglia repopulation did not affect those social behaviors either (Lehmann et al., 2019; Gu et al., 2021).

On the contrary, microglia depletion with clodronate injections within the hippocampus reduced social preference and recognition, a deficit that recovered upon microglia repopulation. Notably, in the same study, mice treated with PLX3397 (3 weeks) did not display social deficits (Torres et al., 2016). A possible explanation for this discrepancy are the inflammatory side effects produced by the intracerebral injection of clodronate (Han et al., 2019), since an inflammatory state might cause reduced social exploration and social withdrawal (Moieni and Eisenberger, 2018).

#### Object and location recognition learning and memory

Recognition memory is typically evaluated with the novel object recognition (NOR) task or its variants such as novel object location (NOL) or object-context discrimination (COD) (see Box 1). While most studies found no effects of microglia depletion in recognition memory, some studies revealed recognition memory deficits depending on age or under specific training conditions (Supplementary Table 5).

Two studies reported learning and memory discrimination deficits in the NOR task in juvenile mice with genetic DT-induced microglia depletion (Parkhurst et al., 2013) and PLX3397 (1 week) (Pinto et al., 2020).

In contrast, in adult animals a large set of studies consistently found no effect of microglia depletion, either pharmacological or genetic, in recognition memory in the NOR and NOL tasks (Acharya et al., 2016; Feng et al., 2016; Gibson et al., 2019; Kakae et al., 2019; Allen et al., 2020; Crapser et al., 2020; De Luca et al., 2020; Worthen et al., 2020; Witcher et al., 2021; Wyatt-Johnson et al., 2021). No effects have been observed upon microglia repopulation either (Elmore et al., 2015; De Luca et al., 2020; Henry et al., 2020). However, two studies did report discrimination learning deficits in adult mice treated with PLX5622 (1 week) (Basilico et al., 2021) and rats treated with PLX3397 (3 weeks) (Yegla et al., 2021) in the NOR and COD tasks, respectively.

The main difference between these two studies and the others are merely procedural. Basilico et al. (2021) and Yegla et al. (2021) relied on a protocol with more than one familiarization session unlike all the studies reported above in which a single familiarization session was administered. By using more familiarization sessions both studies observed an increased object exploration and lack of habituation during the acquisition phase (in microglia depleted animals). These deficits are reminiscent of an inefficient object encoding observed in rats with hippocampal lesions (Broadbent et al., 2009a). Consistently, (Basilico et al., 2021; Yegla et al., 2021) reported decreased glutamatergic activity in the hippocampus. Notably, both the synaptic and learning deficits recovered upon microglia repopulation (Basilico et al., 2021; Yegla et al., 2021). A plausible explanation for the impact of the number of familiarization sessions in revealing the effects of microglia depletion can be found in the observation put forward by Squire et al. (2007) and Cinalli et al. (2020). Indeed, depending on the amount of object exploration during familiarization sessions, weak and strong recognition memories are formed and encoded by distinct brain structures, perirhinal cortex and hippocampus respectively (Squire et al., 2007; Cinalli et al., 2020). It is therefore plausible that microglia particularly support the formation of strong recognition memories.

#### Associative learning and memory

A few studies investigated the effects of microglia depletion on associative memory *via* the fear conditioning procedure (see Box 1). These studies suggest that microglia might not play a major role in the encoding and retrieval of fear memory associations, but it might be critical for the long-term maintenance of aversive fear memories (Supplementary Table 6).

In support of this, four studies using pharmacological microglia depletion found no effects in the expression of fear memory in mice (Elmore et al., 2014; Acharya et al., 2016; Feng et al., 2017; Spangenberg et al., 2019). Instead, a recent study suggests that microglia mediate forgetting of fear memories *via* a complement-cascade dependent phagocytosis of synapses and the support of neurogenesis (Wang C. et al., 2020). The authors found an increased freezing, associated with a greater context-induced reactivation of fear memory-associated engram cells, when microglia were depleted exclusively after training (PLX3397 and genetic DT-induced). Furthermore, two recent studies found that microglia depletion with PLX delayed the extinction of auditory fear memory in mice (Allen et al., 2020) and rats (Yegla et al., 2021). This evidence suggest that the absence of microglia promotes the maintenance of already existing memories by either preventing extinction or forgetting. It must be noted though, that forgetting and extinction are distinct processes involving distinct circuit and molecular neuroadaptations. The first involves the mere passage of time, while the latter implies the acquisition of new learning upon new experience and inhibition of the expression of the original memory (Merlo et al., 2014).

Yegla et al. (2021) also reported that young rats treated with PLX3397 (3 weeks) displayed more freezing in presence of the conditioned auditory cue. On the contrary, aged rats displayed less freezing both to the conditioned context and in presence of the conditioned cue (Yegla et al., 2021). Similar to aged rats, juvenile mice with genetic DT-induced microglia depletion displayed less freezing in the presence of the conditioned cue (Parkhurst et al., 2013).

Altogether, these results suggest that the effects of microglia depletion on fear memory might depend on age, as previously discussed in the context of recognition memory (section 4.1.5). Further studies are needed to corroborate the role of microglia in the forgetting and extinction of associative memories in adult mice. Notably, this research should be also extended to appetitive associative memories.

#### Spatial learning and memory

Effects of microglia depletion in spatial learning and memory assessed in the Barnes Maze (BM) and the Morris Water Maze (MWM) (Box 1) are rather divergent, which might be explained by the duration of microglia depletion, sex, or age (Supplementary Table 7).

Microglia depletion, either by hippocampal clodronate infusion or PLX3397 (1 week), had a transitory negative effect on BM acquisition, not evident after 3 weeks of depletion (Torres et al., 2016). Accordingly, 2–3-weeks of PLX did not affect spatial learning and memory in young mice (Elmore et al., 2014; Dagher et al., 2015; Willis et al., 2020) and rats (Wyatt-Johnson et al., 2021). A noticeable exception was found in aged mice, where PLX5622 (3 weeks) impaired memory retention especially in females (Unger et al., 2018).

On the other hand, longer microglia depletion protocols (up to 24 weeks) improved performance in spatial navigation tests. Treatment with PLX3397 (4–8 weeks) improved MWM acquisition (Elmore et al., 2014; Rice et al., 2015) and PLX5622 (24 weeks) improved MWM retention (Spangenberg et al., 2019). Microglia repopulation also improved MWM retention in aged mice (Elmore et al., 2018) with no evident effects in young mice (Elmore et al., 2015, 2018; Torres et al., 2016; Henry et al., 2020).

Regarding spatial working memory, the available studies reported no deficits in spontaneous alternation in the Y-maze task (Box 1) in mice treated with PLX (Spangenberg et al., 2019; Henry et al., 2020; Li et al., 2020; Willis et al., 2020; Worthen et al., 2020) or rats with genetic DT-induced microglia depletion and subsequent microglia repopulation (De Luca et al., 2020) (Supplementary Table 8).

Altogether, this evidence points to a sex, age and duration-dependent effect of microglia depletion in spatial learning and memory. While microglia depletion was detrimental for spatial memory in females and aged mice, it did not influence or even improved (i.e., long-term depletion) spatial learning in males. Given that sex differences have been observed in microglia density, gene expression and in response to stress (Villa et al., 2018; Han et al., 2021), we advocate for more studies evaluating the interaction between sex and microglia depletion on behavior.

### Impact of microglia depletion during development on adult behavior

**FIGURE 4:**
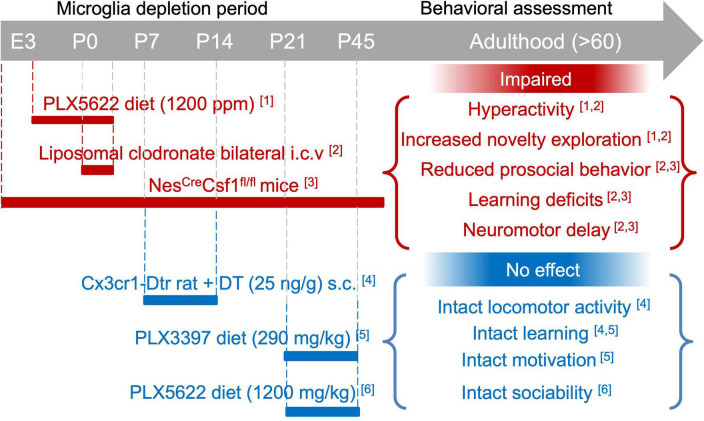
Impact of microglia depletion during development on adult behaviour. Graphic overview of studies that depleted microglia during specific developmental stages (from embryogenesis to adolescence) and assessed the behavioural performance later in time (during adolescence or adulthood). Briefly, microglia depletion during the embryonic and early postnatal periods causes longlasting behavioural and cognitive deficits (*red*), while transient postnatal microglia depletion had no subsequent impact on adult behaviour (*blue*). [1] Rosin et al., 2018; [2] Vanryzin et al., 2016; [3] Kana et al., 2019; [4] Soch et al., 2020; [5] Smith et al., 2020; [6] Ikezu et al., 2021. See section 4.2 for detailed information.

During brain development microglia play a critical role in regulating cell death and survival, neuronal wiring and synaptic plasticity (Marín-Teva et al., 2004; Sierra et al., 2010; Paolicelli et al., 2011; Tay et al., 2017) with long lasting-impact on synaptic function and behavior (Zhan et al., 2014; Basilico et al., 2019). The impact of microglia depletion on adult behavior has been investigated by depleting microglia during specific developmental stages (from embryogenesis to adolescence) and assessing the behavioral performance later in time (during adolescence or adulthood). These studies, reviewed below, suggest that transient microglia depletion during embryonic and early postnatal periods caused profound brain defects and long-lasting behavioral and cognitive deficits, while transient postnatal depletion had no subsequent effect on adult behavior. Refer to Figure 4 for a comprehensive overview of the section.

Specifically, depletion of microglia (PLX5622) from embryonic day 3.5 to birth had a negative effect on pup survival. The surviving pups showed brain alterations, gross craniofacial and dental abnormalities, reflecting macroscopic effects in tissue organization. At the behavioral level, adolescent and adult female mice displayed hyperactivity and ‘anxiolytic-like’ behavior respectively (Rosin et al., 2018).

Microglia depletion *via* intracerebroventricular infusion of clodronate liposomes (P0-4) delayed neuromotor development and induced hyperactivity, increased novelty exploration, social avoidance, and induced learning deficits in juvenile and adult mice (Vanryzin et al., 2016; Nelson and Lenz, 2017). Besides, microglial depletion during this developmental period also led to gross morphological alterations in the brain including ventricle enlargement and thinning of the cortex (Vanryzin et al., 2016; Nelson and Lenz, 2017). Furthermore, specific depletion of cerebellar microglia by genetic depletion of *Csf1* from neural progenitor cells produced deficits in motor learning and social interaction in juvenile mice, in parallel with alterations in cerebellar morphology (Kana et al., 2019).

On the contrary, acute microglia depletion in the Cx3cr1*^Dtr^* transgenic rat model at P7 or P14 did not affect adult locomotor activity, spatial memory nor recognition memory, despite altering neuron and astrocyte densities in the hippocampus (Soch et al., 2020). Likewise, microglia depletion during late postnatal development (P23–45) in mice did not impact appetitive operant learning, reversal learning, motivation and attention in adulthood (Smith et al., 2020) nor social interaction (Ikezu et al., 2021) in the adult rodent.

Altogether, this evidence highlights the critical importance of microglia for a correct brain development and organization of neural tissue.

### Effects of microglia depletion on behavior in animal models of brain pathologies

Microglia are the primary responders to alterations of brain homeostasis and damage. Their responses to insult may actively contribute to synaptic dysfunction and the appearance of neuromotor and cognitive symptoms that are cardinal features of neurologic, neuropsychiatric and neurodegenerative disorders. Microglia depletion strategies provide a tool to understand the contribution of microglia to pathology and a potential therapeutic target to ‘reset’ microglia to a homeostatic phenotype (Cartier et al., 2014; Chung et al., 2015).

Here, we summarized the behavioral effects of depleting microglia in models of acute inflammation (section 4.3.1), early life inflammation (section 4.3.2), acute and chronic stress (section 4.3.3), chronic pain (section 4.3.4), brain injury and stroke (section 4.3.5), irradiation and chemotherapy (section 4.3.6), and neurodegenerative and demyelinating diseases (section 4.3.7).

#### Acute inflammation

It is commonly accepted that microglia response involving the release of cytokines/chemokines contributes to the defense against pathogen infection (Rock et al., 2004). Acute systemic or brain local inflammation induce psychomotor, cognitive, and sickness-related symptoms (Cunningham et al., 2009). However, whether microglia responses mediate the behavioral effects of brain inflammation is still debated (Supplementary Table 9).

In this regard, Yamamoto et al. (2019) found that the psychomotor alterations, social deficits, ‘anxiety-like’ and ‘depressive-like’ behaviors (see Box 1) induced by local injection of the Gram-negative bacterial endotoxin lipopolysaccharide (LPS) or heat-killed Gram-negative bacteria in the cerebellum of rats were alleviated by the treatment with the CSFR1 inhibitor Ki20227. The authors also demonstrated that microglia-mediated increase in neuronal excitability was responsible for the psychomotor behavioral effects after local inflammation. Similarly, Dwyer et al. (2020) found, in two mouse models of Parkinson’s disease (PD), that the reduction in locomotor activity induced by the infusion of LPS in the substantia nigra was ameliorated in microglia depleted mice (PLX3397, 2 weeks + 2 week withdrawal). Another study found that local DREADD-mediated microglia inhibition blocked conditioned place aversion induced by systemic inflammation. These symptoms were mediated by microglia release of prostaglandin that reduced the excitability of striatal neurons (Klawonn et al., 2021).

A different scenario was observed with a systemic LPS-induced inflammation, where microglia depletion, either genetic DT-induced or with PLX5622 (4 weeks) delayed the recovery of locomotor activity levels (Vichaya et al., 2020), suggesting a protective effect of microglia against the effects of systemic inflammation. Indeed, peripheral inflammation may influence the brain through multiple pathways including afferent nerves, interaction with endothelial cells, active transport of immune-derived molecules *via* the BBB and, in some cases, the recruitment of peripheral immune cells into the brain (Vichaya et al., 2020).

The available studies suggest that microglia-induced synaptic plasticity in response to local inflammation drives behavioral alterations, but some of the behavioral effects of a systemic inflammation may develop independently of microglia.

#### Early life inflammation

Inflammation during the perinatal period produces permanent effects in the developing brain (Meyer et al., 2006; Mottahedin et al., 2017). Few studies showed that transient microglia depletion after an early immune challenge may have a beneficial outcome by preventing the insurgence of long-lasting behavioral effects in rodents (Supplementary Table 9).

Specifically, transient microglia depletion after weaning prevented a number of behavioral alteration observed after early life inflammation such as: (i) the repetitive self-grooming behavior and the reduced social preference induced by maternal immune activation [MIA induced by poly(I:C); PLX5622; P21–42] (Ikezu et al., 2021), (ii) the increased impulsivity induced by gestational exposure to high-fat diet (PLX3397; P23–45) (Smith et al., 2020), and (iii) the ‘depressive-like’ symptoms induced by postnatal injection of LPS (PLX3397; P23–45) (Cao et al., 2021). These observations support the notion that the alterations in synaptic maturation and neuronal activity triggered by activated microglia mediate the lasting behavioral effects of early inflammation (Cao et al., 2021; Ikezu et al., 2021).

Moreover, microglia depletion improves the neurodevelopmental outcome and cognitive function in other conditions characterized by increased pro-inflammatory cytokine levels such as cytomegalovirus infection into the fetal brain (Cloarec et al., 2018) and a mouse model of Down Syndrome (Pinto et al., 2020).

These findings suggest that transient microglia depletion might be a strategy to prevent the repercussions of early life inflammation, contributing factors to the insurgence of neurodevelopmental and neuropsychiatric disorders. However, a main limitation is the potential detrimental effects of the absence of microglia for the developing brain (see section 4.2).

#### Acute and chronic stress

Acute and chronic stress is known to induce behavioral and cognitive alterations (Sandi, 2013). Stress promotes proinflammatory microglia which can become ‘primed’ or sensitized, thus producing an exaggerated inflammatory response to a secondary stimulus (Frank et al., 2007; Delpech et al., 2015). For this reason, ‘resetting’ microglia to a homeostatic phenotype, through depletion and/or repopulation, has been proposed as a tool to prevent or improve functional outcomes in conditions of chronic proinflammatory microglia (Barnett et al., 2021) (Supplementary Table 10).

In this regard, microglia depletion before or during stress normalized ‘anxiety-like’ behaviors and social deficits in mice exposed to inescapable electric foot-shocks (Li S. et al., 2021) and chronic social defeat (Lehmann et al., 2019; Weber et al., 2019). Notably, microglia depletion did not alleviate stress-induced ‘depressive-like’ behaviors (Gao et al., 2019; Cai et al., 2020; Gu et al., 2021).

A more complex scenario stems from studies investigating the interaction between stress and the immune status. Specifically, LPS treatment increased ‘depressive-like’ behaviors in non-stressed mice while decreased them in chronically stressed mice. In both cases, microglia depletion reversed these effects (Gao et al., 2019; Cai et al., 2020; Gu et al., 2021). These findings suggest that microglia have a double-edged effect on behavior depending on stress and immune statuses.

A similar double-edged modulation of the cognitive function by microglia has been observed depending on the resiliency of the animal to stress. Specifically, microglia depletion alleviated cognitive deficits, observed in mice expressing learned helplessness, after inescapable foot-shocks. To the contrary microglia depletion compromised working and recognition memory in stress-resilient mice (that did not express learned helplessness) that with intact microglia had preserved cognitive functions (Worthen et al., 2020). Notably, microglia depletion also increased the resilience to stress, reducing the number of mice expressing learned helplessness after inescapable foot-shocks.

Microglia repopulation might be ineffective in eliminating stress-induced sensitization. Lehmann et al. (2019) observed that stress-induced anxiety and social deficits were ameliorated by microglia depletion but re-emerged upon microglia repopulation. The authors suggested that the behavioral effects were mediated by reactive oxygen species (ROS) produced by microglia during stress and that either stress-induced epigenetic marking of the surviving pool of microglia or other factors in the CNS environment or periphery reprogrammed the repopulating microglia to affect behavioral homeostasis. Accordingly, Weber et al. (2019) found that microglia depletion and repopulation prevented an immune challenge sensitization but not stress-induced sensitization, which induced the recruitment of peripheral monocytes and reinforced the recurrence of anxiety.

Further research on chronic stress must focus on the mechanisms of the double-edge modulatory role of microglia on cognitive function and the potential interplay between microglia and peripheral immune cells. Such studies may provide innovative therapeutic targets for stress-related emotional and cognitive disorders.

#### Chronic pain

Chronic pain associated with nerve damage or neurodegeneration is accompanied by local inflammation and the release of pro-inflammatory cytokines (Sommer et al., 1998; Inoue and Tsuda, 2018; Vincenzi et al., 2022). It has been proposed that microglia might contribute to chronic pain by enhancing synaptic transmission in pain pathways (Zhou et al., 2019) and/or neuronal activation in the somatosensory cortex (Hiraga et al., 2020) (Supplementary Table 11).

In support of this, microglia depletion abolished high-frequency stimulation-induced LTP in spinal fibers, thereby reducing pain (Zhou et al., 2019). Microglia depletion also decreased mechanical allodynia and thermal hyperalgesia (see Box 1) in various models of chronic pain including sciatic nerve ligation (Lee et al., 2018; Wang et al., 2018), limb ischemia (Tang et al., 2018), thalamic hemorrhage (Hiraga et al., 2020), chronic social defeat (Sawicki et al., 2019) and high-fat diet (Liang et al., 2019). Furthermore, DREADDs-induced microglia inhibition reduced allodynia and synaptic transmission after nerve injury (Grace et al., 2018; Saika et al., 2020; Yi et al., 2021).

Several studies found that microglia depletion, while preventing the development of chronic pain, does not attenuate already existing pain (Wang et al., 2018; Zhou et al., 2019; Hiraga et al., 2020). A notable exception to this is reported by Lee et al. (2018). The authors found that PLX5622 reversed existing pain after nerve ligation, an effect that was likely related to the additional depletion of macrophages in this model, which typically contribute to the maintenance of chronic pain.

Altogether these results suggest that microglia participate directly in the regulation of synaptic plasticity in pain pathways that are necessary for the induction of chronic pain (Zhou et al., 2019).

#### Irradiation and chemotherapy

Cancer therapy, including chemotherapy and irradiation, induces persistent microglia reactivity and has long-term deleterious effects on neuroplasticity and cognition (Ahles et al., 2012; Gibson and Monje, 2021). Very few studies addressed whether microglia depletion could prevent these behavioral side effects (Supplementary Table 12).

Specifically, four studies reported that microglia depletion restored memory deficits associated with chemotherapy (Allen et al., 2019; Gibson et al., 2019) and cranial irradiation (Acharya et al., 2016; Feng et al., 2016). The authors also observed that microglia depletion normalized alterations in oligodendrocytes and astrocytes that were induced by microglia activation (Gibson et al., 2019) and inhibited brain monocyte accumulation induced by irradiation (Feng et al., 2016), which contributed to the cognitive dysfunction.

Cosmic radiation exposures in astronauts also elicit cognitive impairment, but the causes are largely unknown. Two studies explored whether microglia contributed to cognitive impairment induced by cosmic radiation. Indeed, the depletion of microglia improved contextual memory in mice exposed to helium-irradiation (Krukowski et al., 2018; Allen et al., 2020) (Supplementary Table 12).

#### Brain injury and stroke

Brain trauma induces microglia proliferation to the injured site and the release of factors that mediate healing and inflammatory responses. Some of the microglia responses to injury produce a neuroprotective effect, while others might contribute to the so-called ‘secondary injury’. It is plausible that the synaptic reorganization mediated by microglia after injury contributes to the behavioral deficits associated with damage, which sometimes persist long after the trauma (Bergold, 2016; Paladini et al., 2021). The studies reviewed below suggest that microglia depletion after injury improves the emotional and cognitive function but might delay the neuromotor recovery (Supplementary Table 13).

In support of this, the removal of microglia normalized ‘anxiety-like’ behavior and improved learning after neuronal loss (Rice et al., 2015), ameliorated ‘depressive-like’ symptoms after spinal cord injury (Li et al., 2020) and improved memory deficits after traumatic brain injury (TBI) (Witcher et al., 2021), spinal cord injury (Li et al., 2020) and brain hemorrhage (Shi E. et al., 2019). Notably, an improvement of cognitive function in two models of TBI was also observed upon microglia repopulation (Henry et al., 2020; Willis et al., 2020) where repopulating microglia induced a neuroprotective effect promoting neurogenesis *via* IL-6 signaling (Willis et al., 2020). The improved behavioral outcomes were associated with increased neuronal survival (Li et al., 2020), dendritic spine densities (Rice et al., 2015) and neuronal connectivity (Witcher et al., 2021), suggesting that circuit remodeling by microglia after damage may be detrimental for behavior and cognition. However, two studies reported that microglia depletion failed to restore memory deficits in a model of pilocarpine-induced epilepsy (Wyatt-Johnson et al., 2021) and TBI (Willis et al., 2020).

The effects of microglia depletion on the recovery of motor function after trauma are less encouraging and more controversial. Only a partial improvement of motor recovery with microglia depletion was reported after cerebellar hemorrhage (Xie et al., 2020) and spinal cord injury (Li et al., 2020). Furthermore, the absence of microglia exacerbated motor performance after brain ischemia and spinal cord injury. Motor deficits coexisted with reduced neuronal survival, axonal recovery, delayed astrocyte recovery and immune cell infiltration (Jin et al., 2017; Fu et al., 2020). On the other hand, microglia repopulation had a positive outcome in the recovery of motor performance in a model of TBI (Henry et al., 2020).

In summary, while depleting microglia might impair neuromotor recovery after injury, it might prevent the emotional and cognitive effects secondary to trauma. However, a note of caution should be introduced due to the large methodological differences found between injury models, extent of damage, brain region, age and microglia depletion method and duration.

#### Neurodegenerative and demyelinating disorders

Neurodegenerative disorders lead to the sustained microglia reactivity that may exacerbate protein accumulation, synapse loss and neuronal death. While it is widely accepted that microglia mediate inflammation and contribute to the initiation or progression of neurodegeneration, it is also established that microglia exert a neuroprotective role. For example, microglia promote the clearance of misfolded protein accumulation and phagocytosis of dead cells (Frautschy et al., 1998; Hickman et al., 2018). Based on the above, it is not surprising that depleting microglia in rodent models of neurodegenerative disorders generates heterogeneous effects on motor and cognitive dysfunction (Supplementary Table 14).

##### Neuromotor and demyelinating neurodegenerative disorders

Microglia depletion has been reported to ameliorate motor impairment in the lysosomal storage disease (Berve et al., 2020) and spinocerebellar ataxia (Qu et al., 2017) when administered before the appearance of the symptoms. Microglia depletion also ameliorated motor impairment after the induction of cuprizone demyelinating model of multiple sclerosis (Tahmasebi et al., 2019, 2021). The improved behavioral outcomes were associated with the preservation of nerve fibers (Tahmasebi et al., 2019; Berve et al., 2020), increased post-synaptic densities (Qu et al., 2017), increased re-myelination (Tahmasebi et al., 2019) and reduced neuroinflammation (Qu et al., 2017; Tahmasebi et al., 2019; Berve et al., 2020). Notably, repopulating microglia in a model of white matter progressive inflammation induced a transient motor improvement, although amelioration of catatonia was not evident (Garcia-Agudo et al., 2019). Similarly, pharmacological microglia depletion after EAE induction improved motor outcome in female mice, but symptoms re-emerged upon microglia repopulation (Nissen et al., 2018).

On the other hand, motor outcomes were aggravated by microglia depletion during the early recovery phase in a model of amyotrophic lateral sclerosis (Spiller et al., 2018) and autoimmune encephalitis (EAE) (Rubino et al., 2018).

##### Parkinson’s disease

Microglia depletion after induction of 6-OHDA-PD improved motor performance and reduced ‘depressive-like’ symptoms. This improvement was paralleled by the recovery of glutamatergic and dopaminergic neurotransmission (Oh et al., 2020). Continuous microglia depletion before and after rotenone-induced PD also improved cognitive deficits together with reduced apoptosis, α-synuclein accumulation and astrogliosis (Zhang et al., 2021). On the other hand, the induction of MPTP-PD in microglia depleted mice aggravated motor deficits and increased inflammation, leukocyte infiltration and the loss of dopaminergic neurons (Yang et al., 2018). Notably, the repopulation of microglia in the MPTP-PD model reduced neuronal loss and improved motor function (Li Q. et al., 2021).

##### Huntington’s disease

In a model of Huntington’s disease, long-term microglia depletion before the appearance of the symptomatology improved recognition memory and motor deficits but did not ameliorate stereotypical behavior. The behavioral improvement was associated with reduced astrogliosis and huntingtin accumulation, but increased perineuronal nets (Crapser et al., 2020).

##### Alzheimer’s disease (AD)

In animal models of AD microglia depletion failed to rescue cognitive functions. In aged 3xTg-AD mice microglia depletion modestly improved spatial learning and memory but failed to improve recognition memory and ‘anxiety-like’ symptoms (Dagher et al., 2015). No improvement in spatial memory was observed in aged APP Swedish PS1 dE9 mice (Unger et al., 2018). Additionally, long-term microglia depletion in 5XFAD mice starting before the onset of amyloid plaque pathology also failed to improve working memory and spatial memory and exacerbated ‘anxiolytic-like’ symptoms (Spangenberg et al., 2019). In AD models, microglia proliferate and accumulate surrounding beta amyloid plaques, possibly regulating their formation and/or growth. Sustained microglia depletion was able to reduce Aβ-plaque formation in 5XFAD mice, however some plaques accumulated in blood vessels leading to cerebral amyloid angiopathy (Spangenberg et al., 2019). On the other hand, microglia depletion did not alter Aβ levels and plaque loads in neither aged 3xTg-AD nor in aged APP-PS1 mice (Dagher et al., 2015; Unger et al., 2018; Spangenberg et al., 2019).

Overall, the evidence reviewed suggest that microglia contribute to some extent to behavioral and cognitive dysfunction during the progression of neurodegenerative conditions. However, it is not possible to disregard their neuroprotective role.

## Concluding remarks

Over the last years increasing evidence revealed a role of microglia in regulating synaptic structure and function (also reviewed in Tremblay and Majewska, 2011; Miyamoto et al., 2013; Morris et al., 2013; Tay et al., 2017; Andoh and Koyama, 2021). Recent evidence also suggest that microglia modulate experience-induced plasticity influencing learning and memory (Parkhurst et al., 2013; Wang C. et al., 2020). These evidence provided support to the postulation that microglia, as well as other glial cells, must be included in a comprehensive framework to explore the neurobiological basis of learning and memory, and behavior (Morris et al., 2013; Kol and Goshen, 2021; Wang et al., 2021; Cornell et al., 2022; Ortinski et al., 2022). In this regard, microglia depletion techniques have been critical to study the functions of microglia in the adult brain. In this review, we summarized and discussed their contribution to the current understanding of the role of microglia on synaptic function, learning and memory, and behavior both in physiological and pathological conditions.

The first main finding emerging from these studies is that microglia modulate excitatory neurotransmission in a brain region-dependent fashion. Indeed, in absence of microglia, glutamatergic neurotransmission is reduced in the motor cortex, the hippocampus and the olfactory bulbs but enhanced in the visual cortex and the striatum. Notably, evidence regarding the effect of microglia depletion on inhibitory neurotransmission are still limited. The mechanisms by which microglia modulate synaptic function include the regulation of neurotransmitter receptors trafficking, spine dynamics, perineuronal nets and neurogenesis (see section 3). A recent study also suggests that microglia directly suppress neuronal activity (Badimon et al., 2020).

A second main finding emerging from these studies is that microglia contribute to specific learning and memory processes such as the formation of strong object recognition memories (see section 4.1.5) and the elimination [i.e., forgetting of fear memories; also reviewed in Wang et al. (2021)] or the updating/reorganization of existing associative memories (i.e., extinction of fear memories; see section 4.1.6). Notably, the effects of microglia depletion in learning are more pervasive in juvenile and aged mice, suggesting age-dependent contributions of microglia to learning and memory. On the contrary, microglia do not have a major impact on other behavioral domains such as general locomotor activity, anxiety, sensorimotor function, sociability and working memory. However, these conclusions should be taken with a note of caution due to the many conflicting findings existing in the literature. Indeed, if activity-dependent synaptic plasticity represents the subcellular substrate of learning and memory and microglia regulates synaptic plasticity, it is puzzling that microglia depletion does not always translate into behavioral/learning impairments. Why so? First of all, it should be noted that the fundamental question of how synaptic plasticity translates into learning and behavior, despite years of technical and conceptual advances, is not completely understood (Sweatt, 2016; Humeau and Choquet, 2019). Second, it is important to consider that not all experiences or behaviors require the same amount of plasticity in the brain. For example, running does not induce changes in cerebellar Purkinje synapses, but learning motor skills does (Black et al., 1990; Kolb and Gibb, 2014). This might explain why microglia-induced plasticity does not contribute to general aspects of behavior, such as locomotor activity or anxiety, while instead might contribute to learning or the maintenance of memories. Alternatively, it is possible that microglia participate in cell-wide homeostatic plasticity putatively necessary for the regulation or stabilization of the behavioral change (Sweatt, 2016; Badimon et al., 2020). Studies using comparable methodologies coupled to a rigorous examination of the learning and memory processes are needed to corroborate previous studies. Toward this aim some outstanding issues need to be addressed. The first one is to determine whether the contribution of microglia relates to specific processes of learning acquisition, memory consolidation and maintenance and whether this contribution is similar for different types of learning (e.g., recognition memory, associative memory, and spatial memory). A second question is to determine how age and sex modulate the role of microglia in learning processes. Other pressing questions pertain the limitations of microglia depletion techniques. These include establishing similarities and differences between genetic and pharmacological depletion strategies, understanding the effects of microglia depletion on other cells regulating plasticity such as astrocytes and understanding potential compensatory mechanisms that might take place in absence of microglia (Thiel et al., 2022).

A third main finding, is that microglia depletion, proposed as a strategy to re-establish homeostasis into the diseased brain, produces distinct effects ranging from protective to ineffective or even detrimental. Specifically, microglia depletion restores behavioral and cognitive performance in models of local inflammation, chemotherapy and radiation. It also prevents the emotional and cognitive effects secondary to brain injury or stroke, while in parallel impairs neuromotor recovery. The behavioral effects of microglia depletion on neurodegenerative diseases are rather highly heterogeneous (see section 4.3.7).

To conclude, despite the diversity of microglia functions regulating brain plasticity described in the last decades, microglia depletion in adulthood rarely translates into behavior impairment and many discrepancies are reported. The pleiotropic effects of microglia on behavior reported above can be recapitulated by the sensitivity of these cells to environmental changes, its phenotypic heterogeneity and its multiple effects on different plasticity mechanisms or even the apparently opposite effects on the regulation of synaptic functions (e.g., regulation of both excitatory and inhibitory neurotransmission, regulation of both dendritic spine formation and elimination). In this regard, it has been suggested that low and high levels of cytokines, the immune status and levels of stress modulate behavior and cognition in opposite directions (Yirmiya and Goshen, 2011; Bourgognon and Cavanagh, 2020). Moreover, microglia states (Butovsky and Weiner, 2018) or subtypes (e.g., gray matter vs. white matter) (Badimon et al., 2020)—and possibly others to be described—might play different roles in regulating plasticity and behavior. Potential off-target effects of the available microglia depletion strategies might also have contributed to the observed variability.

For the future, new tools to manipulate microglia with more specificity and with a higher spatial and temporal resolution, such as DREADDs or newly developed adeno-associated viral vectors (Lin et al., 2022) combined with transgenic lines, will become essential. These innovative strategies, coupled with a rigorous theoretical and experimental analysis of the behavioral processes, will be required to comprehend the exact contribution of microglia in learning and memory processes.

## Author contributions

BB, LF, AK, SD, DR, and IR wrote the manuscript. All authors contributed to the article and approved the submitted version.

## Abbreviations

abGC: adult-born granule cells
BBB: blood-brain barrier
BM: Barnes maze
COD: Context-Object Discrimination
CSF1: Colony Stimulating Factor 1
CSF1R: colony stimulating factor 1 receptor
CSF1-i: Colony Stimulating Factor 1 inhibitors
DCX: doublecortin
DREADD: Designer Receptor Exclusively Activated by a Designer Drug
DT: diphtheria toxin
DTR: diphtheria toxin receptor
EAE: autoimmune encephalitis
EPM: Elevated Plus Maze
EPSC: excitatory postsynaptic current
GPCR: G protein-coupled receptors
HSVTK: herpes simplex virus thymidine kinase
IPSC: inhibitory postsynaptic current
LPS: lipopolysaccharide
LTP: long-term potentiation
MWM: Morris Water Maze
NOR: novel object recognition
OF: open field
P: postnatal day
PD: Parkinson’s disease
PV: parvalbumin
PNN: perineuronal nets
TBI: traumatic brain injury.

## References

[B1] Acharya M. M. Green K. N. Allen B. D. Najafi A. R. Syage A. Minasyan H. (2016). Elimination of microglia improves cognitive function following cranial irradiation. *Sci. Rep.* 6:31545. 10.1038/srep31545 27516055 PMC4981848

[B2] Adeluyi A. Guerin L. Fisher M. L. Galloway A. Cole R. D. Chan S. S. L. (2019). Microglia morphology and proinflammatory signaling in the nucleus accumbens during nicotine withdrawal. *Sci. Adv.* 5 1–11. 10.1126/sciadv.aax7031 31633029 PMC6785260

[B3] Ahles T. A. Root J. C. Ryan E. L. (2012). Cancer- and cancer treatment-associated cognitive change: An update on the state of the science. *J. Clin. Oncol.* 30 3675–3686. 10.1200/JCO.2012.43.0116 23008308 PMC3675678

[B4] Ahmari S. E. (2016). Using mice to model obsessive compulsive disorder: From genes to circuits. *Neuroscience* 321 121–137. 10.1016/j.neuroscience.2015.11.009 26562431 PMC4843997

[B5] Akiyoshi R. Wake H. Kato D. Horiuchi H. Ono R. Ikegami A. (2018). Microglia enhance synapse activity to promote local network synchronization. *eNeuro* 5 1–13. 10.1523/ENEURO.0088-18.2018 30406198 PMC6220592

[B6] Ali S. Mansour A. G. Huang W. Queen N. J. Mo X. Anderson J. M. (2020). CSF1R inhibitor PLX5622 and environmental enrichment additively improve metabolic outcomes in middle-aged female mice. *Aging (Albany. NY).* 12 2101–2122. 10.18632/aging.102724 32007953 PMC7041757

[B7] Allen B. D. Apodaca L. A. Syage A. R. Markarian M. Baddour A. A. D. Minasyan H. (2019). Attenuation of neuroinflammation reverses Adriamycin-induced cognitive impairments. *Acta Neuropathol. Commun.* 7 1–15. 10.1186/s40478-019-0838-8 31753024 PMC6868786

[B8] Allen B. D. Syage A. R. Maroso M. Baddour A. A. D. Luong V. Minasyan H. (2020). Mitigation of helium irradiation-induced brain injury by microglia depletion. *J. Neuroinflammation* 17 1–18. 10.1186/s12974-020-01790-9 32429943 PMC7236926

[B9] Andoh M. Koyama R. (2021). Microglia regulate synaptic development and plasticity. *Dev. Neurobiol.* 81 568–590. 10.1002/dneu.22814 33583110 PMC8451802

[B10] Andreou K. E. Soto M. S. Allen D. Economopoulos V. de Bernardi A. Larkin J. R. (2017). Anti-inflammatory microglia/macrophages as a potential therapeutic target in brain metastasis. *Front. Oncol* 7:251. 10.3389/fonc.2017.00251 29164051 PMC5670100

[B11] Armbruster B. N. Li X. Pausch M. H. Herlitze S. Roth B. L. (2007). Evolving the lock to fit the key to create a family of G protein-coupled receptors potently activated by an inert ligand. *Proc. Natl. Acad. Sci. U.S.A.* 104 5163–5168. 10.1073/pnas.0700293104 17360345 PMC1829280

[B12] Ayash S. Schmitt U. Müller M. B. (2020). Chronic social defeat-induced social avoidance as a proxy of stress resilience in mice involves conditioned learning. *J. Psychiatr. Res.* 120 64–71. 10.1016/j.jpsychires.2019.10.001 31634751

[B13] Ayata P. Badimon A. Strasburger H. J. Duff M. K. Montgomery S. E. Loh Y.-H. H. E. H. E. (2018). Epigenetic regulation of brain region-specific microglia clearance activity. *Nat. Neurosci.* 21 1049–1060. 10.1038/s41593-018-0192-3 30038282 PMC6090564

[B14] Badimon A. Strasburger H. J. Ayata P. Chen X. Nair A. Ikegami A. (2020). Negative feedback control of neuronal activity by microglia. *Nature* 586 417–423. 10.1038/s41586-020-2777-8 32999463 PMC7577179

[B15] Balkaya M. Kröber J. M. Rex A. Endres M. (2013). Assessing post-stroke behavior in mouse models of focal ischemia. *J. Cereb. Blood Flow Metab.* 33 330–338. 10.1038/jcbfm.2012.185 23232947 PMC3587814

[B16] Barker G. R. I. Warburton E. C. (2015). Object-in-place associative recognition memory depends on glutamate receptor neurotransmission within two defined hippocampal-cortical circuits: A critical role for AMPA and NMDA receptors in the hippocampus, perirhinal, and prefrontal cortices. *Cereb. Cortex* 25 472–481. 10.1093/cercor/bht245 24035904 PMC4380082

[B17] Barnett A. M. Crews F. T. Coleman L. G. (2021). Microglial depletion and repopulation: A new era of regenerative medicine? *Neural Regen. Res.* 16 1204–1205. 10.4103/1673-5374.300439 33269777 PMC8224128

[B18] Barrot M. (2012). Tests and models of nociception and pain in rodents. *Neuroscience* 211 39–50. 10.1016/j.neuroscience.2011.12.041 22244975

[B19] Basilico B. Ferrucci L. Ratano P. Golia M. T. Grimaldi A. Rosito M. (2021). Microglia control glutamatergic synapses in the adult mouse hippocampus. *Glia* 70 1–23. 10.1002/glia.24101 34661306 PMC9297980

[B20] Basilico B. Pagani F. Grimaldi A. Cortese B. Di Angelantonio S. Weinhard L. (2019). Microglia shape presynaptic properties at developing glutamatergic synapses. *Glia* 67 53–67. 10.1002/glia.23508 30417584

[B21] Bedolla A. Taranov A. Luo F. Wang J. Turcato F. Fugate E. M. (2022). Diphtheria toxin induced but not CSF1R inhibitor mediated microglia ablation model leads to the loss of CSF/ventricular spaces in vivo that is independent of cytokine upregulation. *J. Neuroinflammation* 19:3. 10.1186/s12974-021-02367-w 34983562 PMC8728932

[B22] Belzung C. Griebel G. (2001). Measuring normal and pathological anxiety-like behaviour in mice: A review. *Behav. Brain Res.* 125 141–149. 10.1016/S0166-4328(01)00291-1 11682105

[B23] Bennett R. E. Brody D. L. (2014). Acute reduction of microglia does not alter axonal injury in a mouse model of repetitive concussive traumatic brain injury. *J. Neurotrauma* 31:1647. 10.1089/NEU.2013.3320 24797413 PMC4170981

[B24] Bergold P. J. (2016). Treatment of traumatic brain injury with anti-inflammatory drugs. *Exp. Neurol.* 275 367–380. 10.1016/J.EXPNEUROL.2015.05.024 26112314 PMC6007860

[B25] Berry K. P. Nedivi E. (2017). Spine dynamics: Are they all the same? *Neuron* 96 43–55. 10.1016/j.neuron.2017.08.008 28957675 PMC5661952

[B26] Berve K. West B. L. Martini R. Groh J. (2020). Sex- and region-biased depletion of microglia/macrophages attenuates CLN1 disease in mice. *J. Neuroinflammation* 17 1–17. 10.1186/s12974-020-01996-x 33115477 PMC7594417

[B27] Black J. E. Isaacs K. R. Anderson B. J. Alcantara A. A. Greenough W. T. (1990). Learning causes synaptogenesis, whereas motor activity causes angiogenesis, in cerebellar cortex of adult rats. *Proc. Natl. Acad. Sci. U.S.A.* 87 5568–5572. 10.1073/PNAS.87.14.5568 1695380 PMC54366

[B28] Bliss T. V. P. Lømo T. (1973). Long-lasting potentiation of synaptic transmission in the dentate area of the anaesthetized rabbit following stimulation of the perforant path. *J. Physiol.* 232 331–356. 10.1113/JPHYSIOL.1973.SP010273 4727084 PMC1350458

[B29] Bolton J. L. Short A. K. Othy S. Kooiker C. L. Shao M. Gunn B. G. (2022). Early stress-induced impaired microglial pruning of excitatory synapses on immature CRH-expressing neurons provokes aberrant adult stress responses. *Cell Rep.* 38:110600. 10.1016/J.CELREP.2022.110600 35354026 PMC9014810

[B30] Bourgognon J.-M. Cavanagh J. (2020). The role of cytokines in modulating learning and memory andbrain plasticity. *Brain Neurosci. Adv.* 4:239821282097980. 10.1177/2398212820979802 33415308 PMC7750764

[B31] Bourin M. Hascoët M. (2003). The mouse light/dark box test. *Eur. J. Pharmacol.* 463 55–65. 10.1016/S0014-2999(03)01274-3 12600702

[B32] Broadbent N. J. Gaskin S. Squire L. R. Clark R. E. (2009a). Object recognition memory and the rodent hippocampus. *Learn. Mem.* 17 5–11. 10.1101/lm.1650110 20028732 PMC2807177

[B33] Broadbent N. J. Gaskin S. Squire L. R. Clark R. E. (2009b). Object recognition memory and the rodent hippocampus. *Learn. Mem.* 17 794–800. 10.1101/LM.1650110 20028732 PMC2807177

[B34] Bruttger J. Karram K. Wörtge S. Regen T. Marini F. Hoppmann N. (2015). Genetic cell ablation reveals clusters of local self-renewing microglia in the mammalian central nervous system. *Immunity* 43 92–106. 10.1016/j.immuni.2015.06.012 26163371

[B35] Burn C. C. (2008). What is it like to be a rat? Rat sensory perception and its implications for experimental design and rat welfare. *Appl. Anim. Behav. Sci*. 112, 1–32. 10.1016/j.applanim.2008.02.007

[B36] Butovsky O. Weiner H. L. (2018). Microglial signatures and their role in health and disease. *Nat. Rev. Neurosci.* 19 622–635. 10.1038/s41583-018-0057-5 30206328 PMC7255106

[B37] Butovsky O. Jedrychowski M. P. Moore C. S. Cialic R. Lanser A. J. Gabriely G. (2013). Identification of a unique TGF-β–dependent molecular and functional signature in microglia. *Nat. Neurosci.* 171 131–143. 10.1038/nn.3599 24316888 PMC4066672

[B38] Butowski N. Colman H. De Groot J. F. Omuro A. M. Nayak L. Wen P. Y. (2016). Orally administered colony stimulating factor 1 receptor inhibitor PLX3397 in recurrent glioblastoma: An Ivy foundation early phase clinical trials consortium phase II study. *Neuro. Oncol.* 18 557–564. 10.1093/neuonc/nov245 26449250 PMC4799682

[B39] Cai Z. Ye T. Xu X. Gao M. Zhang Y. Wang D. (2020). Antidepressive properties of microglial stimulation in a mouse model of depression induced by chronic unpredictable stress. *Prog. Neuropsychopharmacol. Biol. Psychiatry* 101:109931. 10.1016/j.pnpbp.2020.109931 32201112

[B40] Cansell C. Stobbe K. Sanchez C. Le Thuc O. Mosser C. A. Ben-Fradj S. (2021). Dietary fat exacerbates postprandial hypothalamic inflammation involving glial fibrillary acidic protein-positive cells and microglia in male mice. *Glia* 69 42–60. 10.1002/glia.23882 32659044

[B41] Cao P. Chen C. Liu A. Shan Q. Zhu X. Jia C. (2021). Early-life inflammation promotes depressive symptoms in adolescence via microglial engulfment of dendritic spines. *Neuron* 109 2573.e–2589.e. 10.1016/j.neuron.2021.06.012 34233151

[B42] Caroni P. Donato F. Muller D. (2012). Structural plasticity upon learning: Regulation and functions. *Nat. Rev. Neurosci.* 13 478–490. 10.1038/nrn3258 22714019

[B43] Cartier N. Lewis C. A. Zhang R. Rossi F. M. V. (2014). The role of microglia in human disease: Therapeutic tool or target? *Acta Neuropathol.* 128 363–380. 10.1007/s00401-014-1330-y 25107477 PMC4131134

[B44] Carulli D. Verhaagen J. (2021). An extracellular perspective on CNS maturation: Perineuronal nets and the control of plasticity. *Int. J. Mol. Sci.* 22:2434. 10.3390/IJMS22052434 33670945 PMC7957817

[B45] Casali B. T. MacPherson K. P. Reed-Geaghan E. G. Landreth G. E. (2020). Microglia depletion rapidly and reversibly alters amyloid pathology by modification of plaque compaction and morphologies. *Neurobiol. Dis.* 142:104956. 10.1016/j.nbd.2020.104956 32479996 PMC7526856

[B46] Chappell-Maor L. Kolesnikov M. Kim J. S. Shemer A. Haimon Z. Grozovski J. (2020). Comparative analysis of CreER transgenic mice for the study of brain macrophages: A case study. *Eur. J. Immunol.* 50 353–362. 10.1002/EJI.201948342 31762013

[B47] Chung W. S. Welsh C. A. Barres B. A. Stevens B. (2015). Do glia drive synaptic and cognitive impairment in disease? *Nat. Neurosci.* 18 1539–1545. 10.1038/nn.4142 26505565 PMC4739631

[B48] Cinalli D. A. Cohen S. J. Guthrie K. Stackman R. W. (2020). Object recognition memory: Distinct yet complementary roles of the mouse CA1 and perirhinal cortex. *Front. Mol. Neurosci.* 13:527543. 10.3389/FNMOL.2020.527543 33192287 PMC7642692

[B49] Cloarec R. Bauer S. Teissier N. Schaller F. Luche H. Courtens S. (2018). In utero administration of drugs targeting microglia improves the neurodevelopmental outcome following cytomegalovirus infection of the rat fetal brain. *Front. Cell. Neurosci.* 12:55. 10.3389/fncel.2018.00055 29559892 PMC5845535

[B50] Cornell J. Salinas S. Huang H. Y. Zhou M. (2022). Microglia regulation of synaptic plasticity and learning and memory. *Neural Regen. Res.* 17 705–716. 10.4103/1673-5374.322423 34472455 PMC8530121

[B51] Corsi G. Picard K. di Castro M. A. Garofalo S. Tucci F. Chece G. (2022). Microglia modulate hippocampal synaptic transmission and sleep duration along the light/dark cycle. *Glia* 70 89–105. 10.1002/glia.24090 34487590 PMC9291950

[B52] Crapser J. D. Ochaba J. Soni N. Reidling J. C. Thompson L. M. Green K. N. (2020). Microglial depletion prevents extracellular matrix changes and striatal volume reduction in a model of Huntington’s disease. *Brain* 143 266–288. 10.1093/brain/awz363 31848580 PMC6935750

[B53] Crawley J. N. (2007). Mouse behavioral assays relevant to the symptoms of autism. *Brain Pathol.* 17 448–459. 10.1111/j.1750-3639.2007.00096.x 17919130 PMC8095652

[B54] Cserép C. Pósfai B. Dénes Á (2021). Shaping neuronal fate: Functional heterogeneity of direct microglia-neuron interactions. *Neuron* 109 222–240. 10.1016/j.neuron.2020.11.007 33271068

[B55] Cunningham C. Campion S. Lunnon K. Murray C. L. Woods J. F. C. Deacon R. M. J. (2009). Systemic inflammation induces acute behavioral and cognitive changes and accelerates neurodegenerative disease. *Biol. Psychiatry* 65:304. 10.1016/J.BIOPSYCH.2008.07.024 18801476 PMC2633437

[B56] Dagher N. N. Najafi A. R. Kayala K. M. N. Elmore M. R. P. White T. E. Medeiros R. (2015). Colony-stimulating factor 1 receptor inhibition prevents microglial plaque association and improves cognition in 3xTg-AD mice. *J. Neuroinflammation* 12 1–14. 10.1186/s12974-015-0366-9 26232154 PMC4522109

[B57] De Biase L. M. Schuebel K. E. Fusfeld Z. H. Jair K. Hawes I. A. Cimbro R. (2017). Local cues establish and maintain region-specific phenotypes of basal ganglia microglia. *Neuron* 95 341.e–356.e. 10.1016/j.neuron.2017.06.020 28689984 PMC5754189

[B58] De Luca S. N. Soch A. Sominsky L. Nguyen T. X. Bosakhar A. Spencer S. J. (2020). Glial remodeling enhances short-term memory performance in Wistar rats. *J. Neuroinflammation* 17 1–18. 10.1186/s12974-020-1729-4 32028971 PMC7006153

[B59] De Luca S. N. Sominsky L. Soch A. Wang H. Ziko I. Rank M. M. (2019). Conditional microglial depletion in rats leads to reversible anorexia and weight loss by disrupting gustatory circuitry. *Brain. Behav. Immun.* 77 77–91. 10.1016/j.bbi.2018.12.008 30578932

[B60] Delpech J. C. Madore C. Nadjar A. Joffre C. Wohleb E. S. Layé S. (2015). Microglia in neuronal plasticity: Influence of stress. *Neuropharmacology* 96 19–28. 10.1016/j.neuropharm.2014.12.034 25582288

[B61] Diaz-Aparicio I. Paris I. Sierra-Torre V. Plaza-Zabala A. Rodríguez-Iglesias N. Márquez-Ropero M. (2020). Microglia actively remodel adult hippocampal neurogenesis through the phagocytosis secretome. *J. Neurosci.* 40 1453–1482. 10.1523/JNEUROSCI.0993-19.2019 31896673 PMC7044727

[B62] Dommergues M. A. Plaisant F. Verney C. Gressens P. (2003). Early microglial activation following neonatal excitotoxic brain damage in mice: A potential target for neuroprotection. *Neuroscience* 121 619–628. 10.1016/S0306-4522(03)00558-X 14568022

[B63] Donocoff R. S. Teteloshvili N. Chung H. Shoulson R. Creusot R. J. (2020). Optimization of tamoxifen-induced Cre activity and its effect on immune cell populations. *Sci. Rep.* 10 1–12. 10.1038/s41598-020-72179-0 32943672 PMC7499195

[B64] Du Y. Brennan F. H. Popovich P. G. Zhou M. (2022). Microglia maintain the normal structure and function of the hippocampal astrocyte network. *Glia* 70 1359–1379. 10.1002/GLIA.24179 35394085 PMC9324808

[B65] Dwyer Z. Rudyk C. Situt D. Beauchamp S. Abdali J. Dinesh A. (2020). Microglia depletion prior to lipopolysaccharide and paraquat treatment differentially modulates behavioral and neuronal outcomes in wild type and G2019S LRRK2 knock-in mice. *Brain, Behav. Immun. Heal.* 5:100079. 10.1016/j.bbih.2020.100079 34589856 PMC8474533

[B66] Elmore M. R. P. Hohsfield L. A. Kramár E. A. Soreq L. Lee R. J. Pham S. T. (2018). Replacement of microglia in the aged brain reverses cognitive, synaptic, and neuronal deficits in mice. *Aging Cell* 17:e12832. 10.1111/acel.12832 30276955 PMC6260908

[B67] Elmore M. R. P. Lee R. J. West B. L. Green K. N. (2015). Characterizing newly repopulated microglia in the adult mouse?: Impacts on animal behavior. Cell morphology, and neuroinflammation. *PLoS One* 10:e0122912. 10.1371/journal.pone.0122912 25849463 PMC4388515

[B68] Elmore M. R. P. Najafi A. R. Koike M. A. Dagher N. N. Spangenberg E. E. Rice R. A. (2014). Colony-stimulating factor 1 receptor signaling is necessary for microglia viability, unmasking a microglia progenitor cell in the adult brain. *Neuron* 82 380–397. 10.1016/j.neuron.2014.02.040 24742461 PMC4161285

[B69] Feng X. Jopson T. D. Paladini M. S. Liu S. West B. L. Gupta N. (2016). Colony-stimulating factor 1 receptor blockade prevents fractionated whole-brain irradiation-induced memory deficits. *J. Neuroinflammation* 13 1–13. 10.1186/s12974-016-0671-y 27576527 PMC5006433

[B70] Feng X. Valdearcos M. Uchida Y. Lutrin D. Maze M. Koliwad S. K. (2017). Microglia mediate postoperative hippocampal inflammation and cognitive decline in mice. *JCI Insight* 2 1–12. 10.1172/jci.insight.91229 28405620 PMC5374063

[B71] FrankM. G.BarattaM. V.SprungerD. B.WatkinsL. R.MaierS. F. (2007). Microglia serve as a neuroimmune substrate for stress-induced potentiation of CNS pro-inflammatory cytokine responses. *Brain. Behav. Immun.* 21 47–59. 10.1016/J.BBI.2006.03.005 16647243

[B72] Frautschy S. A. Yang F. Irrizarry M. Hyman B. Saido T. C. Hsiao K. (1998). Microglial response to amyloid plaques in APPsw transgenic mice. *Am. J. Pathol* 152:307. 9422548 PMC1858113

[B73] Frost J. L. Schafer D. P. (2016). Microglia: Architects of the developing nervous system. *Trends Cell Biol.* 26 587–597. 10.1016/j.tcb.2016.02.006 27004698 PMC4961529

[B74] Fu H. Zhao Y. Hu D. Wang S. Yu T. Zhang L. (2020). Depletion of microglia exacerbates injury and impairs function recovery after spinal cord injury in mice. *Cell Death Dis.* 11:528. 10.1038/s41419-020-2733-4 32661227 PMC7359318

[B75] Gao M. Hu P. Cai Z. Wu Y. Wang D. Hu W. (2019). Identification of a microglial activation-dependent antidepressant effect of amphotericin B liposome. *Neuropharmacology* 151 33–44. 10.1016/j.neuropharm.2019.04.005 30954529

[B76] Garcia-Agudo L. F. Janova H. Sendler L. E. Arinrad S. Steixner A. A. Hassouna I. (2019). Genetically induced brain inflammation by Cnp deletion transiently benefits from microglia depletion. *FASEB J.* 33 8634–8647. 10.1096/fj.201900337R 31090455

[B77] Gawel K. Gibula E. Marszalek-Grabska M. Filarowska J. Kotlinska J. H. (2019). Assessment of spatial learning and memory in the Barnes maze task in rodents—methodological consideration. *Naunyn Schmiedebergs Arch. Pharmacol.* 392 1–18. 10.1007/s00210-018-1589-y 30470917 PMC6311199

[B78] Gibson E. M. Monje M. (2021). Microglia in cancer therapy-related cognitive impairment. *Trends Neurosci.* 44 441–451. 10.1016/j.tins.2021.02.003 33674135 PMC8593823

[B79] Gibson E. M. Nagaraja S. Ocampo A. Tam L. T. Wood L. S. Pallegar P. N. (2019). Methotrexate chemotherapy induces persistent tri-glial dysregulation that underlies chemotherapy-related cognitive impairment. *Cell* 176 43.e–55.e. 10.1016/j.cell.2018.10.049 30528430 PMC6329664

[B80] Ginhoux F. Prinz M. (2015). Origin of microglia: Current concepts and past controversies. *Cold Spring Harb. Perspect. Biol.* 7:a020537. 10.1101/cshperspect.a020537 26134003 PMC4526747

[B81] Ginhoux F. Greter M. Leboeuf M. Nandi S. See P. Gokhan S. (2010). Fate mapping analysis reveals that adult microglia derive from primitive macrophages. *Science* 330 841–845. 10.1126/science.1194637 20966214 PMC3719181

[B82] Gogolla N. Caroni P. Lüthi A. Herry C. (2009). Perineuronal nets protect fear memories from erasure. *Science* 325 1258–1261. 10.1126/SCIENCE.1174146 19729657

[B83] Goldmann T. Wieghofer P. Jordão M. J. C. Prutek F. Hagemeyer N. Frenzel K. (2016). Origin, fate and dynamics of macrophages at CNS interfaces. *Nat. Immunol.* 17:797. 10.1038/NI.3423 27135602 PMC4968048

[B84] Grabert K. Michoel T. Karavolos M. H. Clohisey S. Kenneth Baillie J. Stevens M. P. (2016). Microglial brain regionâ ’dependent diversity and selective regional sensitivities to aging. *Nat. Neurosci.* 19 504–516. 10.1038/nn.4222 26780511 PMC4768346

[B85] Grace P. M. Strand K. A. Galer E. L. Urban D. J. Wang X. Baratta M. V. (2016). Morphine paradoxically prolongs neuropathic pain in rats by amplifying spinal NLRP3 inflammasome activation. *Proc. Natl. Acad. Sci. U.S.A.* 113 E3441–E3450. 10.1073/pnas.1602070113 27247388 PMC4914184

[B86] Grace P. M. Wang X. Strand K. A. Baratta M. V. Zhang Y. Galer E. L. (2018). DREADDed microglia in pain: Implications for spinal inflammatory signaling in male rats. *Exp. Neurol.* 304 125–131. 10.1016/j.expneurol.2018.03.005 29530713 PMC5916033

[B87] Gratuze M. Chen Y. Parhizkar S. Jain N. Strickland M. R. Serrano J. R. (2021). Activated microglia mitigate aβ-associated tau seeding and spreading. *J. Exp. Med.* 218:e20210542. 10.1084/jem.20210542 34100905 PMC8190588

[B88] Green K. N. Hume D. A. (2021). On the utility of CSF1R inhibitors. *Natl. Acad. Sci.* 118:e2019695118. 10.1073/pnas.2019695118 33446486 PMC7848745

[B89] Green K. N. Crapser J. D. Hohsfield L. A. (2020). To kill a microglia: A Case for CSF1R inhibitors. *Trends Immunol.* 41 771–784. 10.1016/j.it.2020.07.001 32792173 PMC7484341

[B90] Green K. N. Najafi A. R. Crapser J. Jiang S. Ng W. Mortazavi A. (2018). A limited capacity for microglial repopulation in the adult brain. *Glia* 66 2385–2396. 10.1002/glia.23477 30370589 PMC6269202

[B91] Gu Y. Ye T. Tan P. Tong L. Ji J. Gu Y. (2021). Tolerance-inducing effect and properties of innate immune stimulation on chronic stress-induced behavioral abnormalities in mice. *Brain. Behav. Immun.* 91 451–471. 10.1016/j.bbi.2020.11.002 33157258

[B92] Hagemeyer N. Hanft K. M. Akriditou M. A. Unger N. Park E. S. Stanley E. R. (2017). Microglia contribute to normal myelinogenesis and to oligodendrocyte progenitor maintenance during adulthood. *Acta Neuropathol.* 134 441–458. 10.1007/s00401-017-1747-1 28685323 PMC5951721

[B93] Han J. Fan Y. Zhou K. Blomgren K. Harris R. A. (2021). Uncovering sex differences of rodent microglia. *J. Neuroinflammation* 18 1–11. 10.1186/s12974-021-02124-z 33731174 PMC7972194

[B94] Han J. Fan Y. Zhou K. Zhu K. Blomgren K. Lund H. (2020). Underestimated peripheral effects following pharmacological and conditional genetic microglial depletion. *Int. J. Mol. Sci.* 21 1–16. 10.3390/ijms21228603 33203068 PMC7696443

[B95] Han X. Li Q. Lan X. El-Mufti L. Ren H. Wang J. (2019). Microglial depletion with clodronate liposomes increases proinflammatory cytokine levels, induces astrocyte activation, and damages blood vessel integrity. *Mol. Neurobiol.* 56 6184–6196. 10.1007/s12035-019-1502-9 30734229 PMC6684378

[B96] Hayashi Y. Koyanagi S. Kusunose N. Okada R. Wu Z. Tozaki-Saitoh H. (2013). The intrinsic microglial molecular clock controls synaptic strength via the circadian expression of cathepsin S. *Sci. Rep.* 3:2744. 10.1038/srep02744 24067868 PMC3783043

[B97] Heldmann U. Mine Y. Kokaia Z. Ekdahl C. T. Lindvall O. (2011). Selective depletion of Mac-1-expressing microglia in rat subventricular zone does not alter neurogenic response early after stroke. *Exp. Neurol.* 229 391–398. 10.1016/j.expneurol.2011.03.005 21419118

[B98] Henry R. J. Ritzel R. M. Barrett J. P. Doran S. J. Jiao Y. Leach J. B. (2020). Microglial depletion with CSF1R inhibitor during chronic phase of experimental traumatic brain injury reduces neurodegeneration and neurological deficits. *J. Neurosci.* 40 2960–2974. 10.1523/JNEUROSCI.2402-19.2020 32094203 PMC7117897

[B99] Heppner F. L. Greter M. Marino D. Falsig J. Raivich G. Hövelmeyer N. (2005). Experimental autoimmune encephalomyelitis repressed by microglial paralysis. *Nat. Med.* 11 146–152. 10.1038/nm1177 15665833

[B100] Herz J. Filiano A. J. Smith A. Yogev N. Kipnis J. (2017). Myeloid cells in the central nervous system. *Immunity* 46 943–956. 10.1016/j.immuni.2017.06.007 28636961 PMC5657250

[B101] Hickman S. Izzy S. Sen P. Morsett L. El Khoury J. (2018). Microglia in neurodegeneration. *Nat. Neurosci.* 21 1359–1369. 10.1038/s41593-018-0242-x 30258234 PMC6817969

[B102] Hiraga S. I. Itokazu T. Hoshiko M. Takaya H. Nishibe M. Yamashita T. (2020). Microglial depletion under thalamic hemorrhage ameliorates mechanical allodynia and suppresses aberrant axonal sprouting. *JCI Insight* 5:e131801. 10.1172/jci.insight.131801 32051342 PMC7098795

[B103] Huang Y. Xu Z. Xiong S. Sun F. Qin G. Hu G. (2018). Repopulated microglia are solely derived from the proliferation of residual microglia after acute depletion. *Nat. Neurosci.* 21 530–540. 10.1038/s41593-018-0090-8 29472620

[B104] Humeau Y. Choquet D. (2019). The next generation of approaches to investigate the link between synaptic plasticity and learning. *Nat. Neurosci.* 22 1536–1543. 10.1038/s41593-019-0480-6 31477899

[B105] Ikezu S. Yeh H. Delpech J. C. Woodbury M. E. Van Enoo A. A. Ruan Z. (2021). Inhibition of colony stimulating factor 1 receptor corrects maternal inflammation-induced microglial and synaptic dysfunction and behavioral abnormalities. *Mol. Psychiatry* 26 1808–1831. 10.1038/s41380-020-0671-2 32071385 PMC7431382

[B106] Inoue K. Tsuda M. (2018). Microglia in neuropathic pain: Cellular and molecular mechanisms and therapeutic potential. *Nat. Rev. Neurosci.* 19 138–152. 10.1038/nrn.2018.2 29416128

[B107] Janova H. Arinrad S. Balmuth E. Mitjans M. Hertel J. Habes M. (2018). Microglia ablation alleviates myelin-associated catatonic signs in mice. *J. Clin. Invest.* 128 734–745. 10.1172/JCI97032 29252214 PMC5785265

[B108] Jiang C. Wang Z. N. Kang Y. C. Chen Y. Lu W. X. Ren H. J. (2022). Ki20227 aggravates apoptosis, inflammatory response, and oxidative stress after focal cerebral ischemia injury. *Neural Regen. Res.* 17 137–143. 10.4103/1673-5374.314318 34100449 PMC8451550

[B109] Jin W. N. Shi S. X. Y. Li Z. Li M. Wood K. Gonzales R. J. (2017). Depletion of microglia exacerbates postischemic inflammation and brain injury. *J. Cereb. Blood Flow Metab.* 37 2224–2236. 10.1177/0271678X17694185 28273719 PMC5444553

[B110] Kakae M. Tobori S. Morishima M. Nagayasu K. Shirakawa H. Kaneko S. (2019). Depletion of microglia ameliorates white matter injury and cognitive impairment in a mouse chronic cerebral hypoperfusion model. *Biochem. Biophys. Res. Commun.* 514 1040–1044. 10.1016/j.bbrc.2019.05.055 31097227

[B111] Kana V. Desland F. A. Casanova-Acebes M. Ayata P. Badimon A. Nabel E. (2019). CSF-1 controls cerebellar microglia and is required for motor function and social interaction. *J. Exp. Med.* 216 2265–2281. 10.1084/jem.20182037 31350310 PMC6781012

[B112] Kierdorf K. Erny D. Goldmann T. Sander V. Schulz C. Perdiguero E. G. (2013). Microglia emerge from erythromyeloid precursors via Pu.1-and Irf8-dependent pathways. *Nat. Neurosci.* 16 273–280. 10.1038/nn.3318 23334579

[B113] Klawonn A. M. Fritz M. Castany S. Pignatelli M. Canal C. Similä F. (2021). Microglial activation elicits a negative affective state through prostaglandin-mediated modulation of striatal neurons. *Immunity* 54 225.e–234.e. 10.1016/j.immuni.2020.12.016 33476547

[B114] Kol A. Goshen I. (2021). The memory orchestra: The role of astrocytes and oligodendrocytes in parallel to neurons. *Curr. Opin. Neurobiol.* 67 131–137. 10.1016/j.conb.2020.10.022 33260057 PMC7611987

[B115] Kolb B. Gibb R. (2014). Searching for the principles of brain plasticity and behavior. *Cortex* 58 251–260. 10.1016/J.CORTEX.2013.11.012 24457097

[B116] Kraeuter A. K. Guest P. C. Sarnyai Z. (2019). The Y-Maze for assessment of spatial working and reference memory in mice. *Methods Mol. Biol.* 1916 105–111. 10.1007/978-1-4939-8994-2_10 30535688

[B117] Kreutzberg G. W. (1996). Microglia: A sensor for pathological events in the CNS. *Trends Neurosci.* 19 312–318. 10.1016/0166-2236(96)10049-7 8843599

[B118] Krishnan V. Nestler E. J. (2011). Animal models of depression: Molecular perspectives. *Curr. Top. Behav. Neurosci.* 7 121–147. 10.1007/7854_2010_108 21225412 PMC3270071

[B119] Krukowski K. Feng X. Paladini M. S. Chou A. Sacramento K. Grue K. (2018). Temporary microglia-depletion after cosmic radiation modifies phagocytic activity and prevents cognitive deficits. *Sci. Rep.* 8 1–13. 10.1038/s41598-018-26039-7 29777152 PMC5959907

[B120] LeDoux J. (2003). The emotional brain, fear, and the amygdala. *Cell. Mol. Neurobiol.* 23 727–738. 10.1023/A:1025048802629 14514027 PMC11530156

[B121] Lee S. H. Shi X. Q. Fan A. West B. Zhang J. (2018). Targeting macrophage and microglia activation with colony stimulating factor 1 receptor inhibitor is an effective strategy to treat injury-triggered neuropathic pain. *Mol. Pain* 14:1744806918764979. 10.1177/1744806918764979 29546785 PMC5858622

[B122] Lehmann M. L. Weigel T. K. Poffenberger C. N. Herkenham M. (2019). The behavioral sequelae of social defeat require microglia and are driven by oxidative stress in mice. *J. Neurosci.* 39 5594–5605. 10.1523/JNEUROSCI.0184-19.2019 31085604 PMC6616288

[B123] Lei F. Cui N. Zhou C. Chodosh J. Vavvas D. G. Paschalis E. I. (2021). Reply to green and hume: Nonmicroglia peripheral immune effects of short-term CSF1R inhibition with PLX5622. *Proc. Natl. Acad. Sci. U.S.A.* 118 10–12. 10.1073/pnas.2020660118 33446487 PMC7848750

[B124] Lei F. Cui N. Zhou C. Chodosh J. Vavvas D. G. Paschalis E. I. (2020). CSF1R inhibition by a small-molecule inhibitor is not microglia specific; Affecting hematopoiesis and the function of macrophages. *Proc. Natl. Acad. Sci. U.S.A.* 117 23336–23338. 10.1073/pnas.1922788117 32900927 PMC7519218

[B125] Li Q. Shen C. Liu Z. Ma Y. Wang J. Dong H. (2021). Partial depletion and repopulation of microglia have different effects in the acute MPTP mouse model of Parkinson’s disease. *Cell Prolif.* 54 1–16. 10.1111/cpr.13094 34312932 PMC8349650

[B126] Li S. Liao Y. Dong Y. Li X. Li J. Cheng Y. (2021). Microglial deletion and inhibition alleviate behavior of post-traumatic stress disorder in mice. *J. Neuroinflammation* 18 1–14. 10.1186/s12974-020-02069-9 33402212 PMC7786489

[B127] Li Y. Ritzel R. M. Khan N. Cao T. He J. Lei Z. (2020). Delayed microglial depletion after spinal cord injury reduces chronic inflammation and neurodegeneration in the brain and improves neurological recovery in male mice. *Theranostics* 10 11376–11403. 10.7150/thno.49199 33052221 PMC7545988

[B128] Liang Y. J. Feng S. Y. Qi Y. P. Li K. Jin Z. R. Jing H. B. (2019). Contribution of microglial reaction to increased nociceptive responses in high-fat-diet (HFD)-induced obesity in male mice. *Brain. Behav. Immun.* 80 777–792. 10.1016/j.bbi.2019.05.026 31108168

[B129] Lim S. H. Park E. You B. Jung Y. Park A. R. Park S. G. (2013). Neuronal synapse formation induced by microglia and interleukin 10. *PLoS One* 8:e81218. 10.1371/journal.pone.0081218 24278397 PMC3838367

[B130] Lin R. Zhou Y. Yan T. Wang R. Li H. Wu Z. (2022). Directed evolution of adeno-associated virus for efficient gene delivery to microglia. *Nat. Methods* 19 976–985. 10.1038/s41592-022-01547-7 35879607

[B131] Liu Y. J. Spangenberg E. E. Tang B. Holmes T. C. Green K. N. Xu X. (2021). Microglia elimination increases neural circuit connectivity and activity in adult mouse cortex. *J. Neurosci.* 41 1274–1287. 10.1523/JNEUROSCI.2140-20.2020 33380470 PMC7888230

[B132] Liu Y. Given K. S. Dickson E. L. Owens G. P. Macklin W. B. Bennett J. L. (2019). Concentration-dependent effects of CSF1R inhibitors on oligodendrocyte progenitor cells ex vivo and in vivo. *Exp. Neurol.* 318 32–41. 10.1016/j.expneurol.2019.04.011 31029597 PMC6615458

[B133] Lledo P. M. Alonso M. Grubb M. S. (2006). Adult neurogenesis and functional plasticity in neuronal circuits. *Nat. Rev. Neurosci.* 7 179–193. 10.1038/nrn1867 16495940

[B134] Lund H. Pieber M. Parsa R. Han J. Grommisch D. Ewing E. (2018). Competitive repopulation of an empty microglial niche yields functionally distinct subsets of microglia-like cells. *Nat. Commun.* 9:4845. 10.1038/s41467-018-07295-7 30451869 PMC6242869

[B135] Ma X. Chen K. Cui Y. Huang G. Nehme A. Zhang L. (2020). Depletion of microglia in developing cortical circuits reveals its critical role in glutamatergic synapse development, functional connectivity, and critical period plasticity. *J. Neurosci. Res.* 98 1968–1986. 10.1002/jnr.24641 32594561

[B136] Marinelli S. Basilico B. Marrone M. C. Ragozzino D. (2019). Microglia-neuron crosstalk: Signaling mechanism and control of synaptic transmission. *Semin. Cell Dev. Biol.* 94 138–151. 10.1016/j.semcdb.2019.05.017 31112798

[B137] Marín-Teva J. L. Dusart I. Colin C. Gervais A. Van Rooijen N. Mallat M. (2004). Microglia promote the death of developing purkinje cells. *Neuron* 41 535–547. 10.1016/S0896-6273(04)00069-8 14980203

[B138] Merlo E. Milton A. L. Goozée Z. Y. Theobald D. E. Everitt B. J. (2014). Reconsolidation and extinction are dissociable and mutually exclusive processes: Behavioral and molecular evidence. *J. Neurosci.* 34 2422–2431. 10.1523/JNEUROSCI.4001-13.2014 24523532 PMC3921417

[B139] Meyer U. Nyffeler M. Engler A. Urwyler A. Schedlowski M. Knuesel I. (2006). The time of prenatal immune challenge determines the specificity of inflammation-mediated brain and behavioral pathology. *J. Neurosci.* 26 4752–4762. 10.1523/JNEUROSCI.0099-06.2006 16672647 PMC6674174

[B140] Miyamoto A. Wake H. Ishikawa A. W. Eto K. Shibata K. Murakoshi H. (2016). Microglia contact induces synapse formation in developing somatosensory cortex. *Nat. Commun.* 7 1–12. 10.1038/ncomms12540 27558646 PMC5007295

[B141] Miyamoto A. Wake H. Moorhouse A. J. Nabekura J. (2013). Microglia and synapse interactions: Fine tuning neural circuits and candidate molecules. *Front. Cell. Neurosci.* 7:70. 10.3389/fncel.2013.00070 23720611 PMC3654203

[B142] Moieni M. Eisenberger N. I. (2018). Effects of inflammation on social processes and implications for health. *Ann. N.Y. Acad. Sci.* 1428 5-13. 10.1111/nyas.13864 29806109 PMC6158086

[B143] Montero M. González B. Zimmer J. (2009). Immunotoxic depletion of microglia in mouse hippocampal slice cultures enhances ischemia-like neurodegeneration. *Brain Res.* 1291 140–152. 10.1016/j.brainres.2009.06.097 19595678

[B144] Morris G. P. Clark I. A. Zinn R. Vissel B. (2013). Microglia: A new frontier for synaptic plasticity, learning and memory, and neurodegenerative disease research. *Neurobiol. Learn. Mem.* 105 40–53. 10.1016/j.nlm.2013.07.002 23850597

[B145] Mottahedin A. Ardalan M. Chumak T. Riebe I. Ek J. Mallard C. (2017). Effect of neuroinflammation on synaptic organization and function in the developing brain: Implications for neurodevelopmental and neurodegenerative disorders. *Front. Cell. Neurosci* 11:190. 10.3389/fncel.2017.00190 28744200 PMC5504097

[B146] Nagy A. (2000). Cre recombinase: The universal reagent for genome tailoring. *Genesis* 26 99–109. 10.1002/(SICI)1526-968X(200002)26:2 10686599

[B147] Nelson L. H. Lenz K. M. (2017). Microglia depletion in early life programs persistent changes in social, mood-related, and locomotor behavior in male and female rats. *Behav. Brain Res.* 316 279–293. 10.1016/j.bbr.2016.09.006 27613230 PMC6103217

[B148] Neniskyte U. Gross C. T. (2017). Errant gardeners: Glial-cell-dependent synaptic pruning and neurodevelopmental disorders. *Nat. Rev. Neurosci.* 18 658–670. 10.1038/nrn.2017.110 28931944

[B149] Nguyen P. T. Dorman L. C. Pan S. Vainchtein I. D. Han R. T. Nakao-Inoue H. (2020). Microglial remodeling of the extracellular matrix promotes synapse plasticity. *Cell* 182 388.e–403.e. 10.1016/j.cell.2020.05.050 32615087 PMC7497728

[B150] Nissen J. C. Thompson K. K. West B. L. Tsirka S. E. (2018). Csf1R inhibition attenuates experimental autoimmune encephalomyelitis and promotes recovery. *Exp. Neurol.* 307 24–36. 10.1016/j.expneurol.2018.05.021 29803827 PMC6380683

[B151] Oh S. J. Ahn H. Jung K. H. Han S. J. Nam K. R. Kang K. J. (2020). Evaluation of the neuroprotective effect of microglial depletion by CSF-1R inhibition in a Parkinson’s animal model. *Mol. Imaging Biol.* 22 1031–1042. 10.1007/s11307-020-01485-w 32086763

[B152] Ortinski P. I. Reissner K. J. Turner J. Anderson T. A. Scimemi A. (2022). Control of complex behavior by astrocytes and microglia. *Neurosci. Biobehav. Rev.* 137:104651. 10.1016/J.NEUBIOREV.2022.104651 35367512 PMC9119927

[B153] Otxoa-de-Amezaga A. Miró-Mur F. Pedragosa J. Gallizioli M. Justicia C. Gaja-Capdevila N. (2019). Microglial cell loss after ischemic stroke favors brain neutrophil accumulation. *Acta Neuropathol.* 137 321–341. 10.1007/S00401-018-1954-4 30580383 PMC6513908

[B154] Paladini M. S. Feng X. Krukowski K. Rosi S. (2021). Microglia depletion and cognitive functions after brain injury: From trauma to galactic cosmic ray. *Neurosci. Lett.* 741:135462. 10.1016/j.neulet.2020.135462 33259927

[B155] Paolicelli R. C. Bolasco G. Pagani F. Maggi L. Scianni M. Panzanelli P. (2011). Synaptic pruning by microglia is necessary for normal brain development. *Science* 333 1456–1458. 10.1126/science.1202529 21778362

[B156] Parkhurst C. N. Yang G. Ninan I. Savas J. N. Yates J. R. Lafaille J. J. (2013). Microglia promote learning-dependent synapse formation through brain-derived neurotrophic factor. *Cell* 155 1596–1609. 10.1016/J.CELL.2013.11.030 24360280 PMC4033691

[B157] Parkhurst C. N. Yang G. Ninan I. Savas J. N. Yates J. R. Lafaille J. J. (2014). NIH Public Access. *Cell* 155 1596–1609. 10.1016/j.cell.2013.11.030.Microglia PMC4033691 24360280

[B158] Pfeiffer T. Avignone E. Nägerl U. V. (2016). Induction of hippocampal long-term potentiation increases the morphological dynamics of microglial processes and prolongs their contacts with dendritic spines. *Sci. Rep.* 6 1–9. 10.1038/srep32422 27604518 PMC5015055

[B159] Pinto B. Morelli G. Rastogi M. Savardi A. Fumagalli A. Petretto A. (2020). Rescuing over-activated microglia restores cognitive performance in juvenile animals of the Dp(16) mouse model of down syndrome. *Neuron* 108 887.e–904.e. 10.1016/j.neuron.2020.09.010 33027640 PMC7736620

[B160] Prinz M. Masuda T. Wheeler M. A. Quintana F. J. (2021). Microglia and central nervous system-associated macrophages-from origin to disease modulation. *Annu. Rev. Immunol.* 39 251–277. 10.1146/ANNUREV-IMMUNOL-093019-110159 33556248 PMC8085109

[B161] Qu W. Johnson A. Kim J. H. Lukowicz A. Svedberg D. Cvetanovic M. (2017). Inhibition of colony-stimulating factor 1 receptor early in disease ameliorates motor deficits in SCA1 mice. *J. Neuroinflammation* 14 1–11. 10.1186/s12974-017-0880-z 28545543 PMC5445366

[B162] Reshef R. Kreisel T. Beroukhim Kay D. Yirmiya R. (2014). Microglia and their CX3CR1 signaling are involved in hippocampal- but not olfactory bulb-related memory and neurogenesis. *Brain. Behav. Immun.* 41 239–250. 10.1016/j.bbi.2014.04.009 24933434

[B163] Reshef R. Kudryavitskaya E. Shani-Narkiss H. Isaacson B. Rimmerman N. Mizrahi A. (2017). The role of microglia and their CX3CR1 signaling in adult neurogenesis in the olfactory bulb. *Elife* 6 1–30. 10.7554/eLife.30809 29251592 PMC5734876

[B164] Rice R. A. Pham J. Lee R. J. Najafi A. R. West B. L. Green K. N. (2017). Microglial repopulation resolves inflammation and promotes brain recovery after injury. *Glia* 65 931–944. 10.1002/glia.23135 28251674 PMC5395311

[B165] Rice R. A. Spangenberg E. E. Yamate-Morgan H. Lee R. J. Arora R. P. S. Hernandez M. X. (2015). Elimination of microglia improves functional outcomes following extensive neuronal loss in the hippocampus. *J. Neurosci.* 35 9977–9989. 10.1523/JNEUROSCI.0336-15.2015 26156998 PMC4495246

[B166] Rock R. B. Gekker G. Hu S. Sheng W. S. Cheeran M. Lokensgard J. R. (2004). Role of microglia in central nervous system infections. *Clin. Microbiol. Rev*. 17, 942–964. 10.1128/CMR.17.4.942-964.2004 15489356 PMC523558

[B167] Rodríguez-Iglesias N. Sierra A. Valero J. (2019). Rewiring of memory circuits: Connecting adult newborn neurons with the help of microglia. *Front. Cell Dev. Biol.* 7:24. 10.3389/fcell.2019.00024 30891446 PMC6411767

[B168] Rosin J. M. Vora S. R. Kurrasch D. M. (2018). Depletion of embryonic microglia using the CSF1R inhibitor PLX5622 has adverse sex-specific effects on mice, including accelerated weight gain, hyperactivity and anxiolytic-like behaviour. *Brain. Behav. Immun.* 73 682–697. 10.1016/j.bbi.2018.07.023 30056204

[B169] Roth B. L. (2016). DREADDs for Neuroscientists. *Neuron* 89 683–694. 10.1016/j.neuron.2016.01.040 26889809 PMC4759656

[B170] Rubino S. J. Mayo L. Wimmer I. Siedler V. Brunner F. Hametner S. (2018). Acute microglia ablation induces neurodegeneration in the somatosensory system. *Nat. Commun* 9:4578. 10.1038/s41467-018-05929-4 30385785 PMC6212411

[B171] Saika F. Matsuzaki S. Kobayashi D. Ideguchi Y. Nakamura T. Y. Kishioka S. (2020). Chemogenetic regulation of CX3CR1-expressing microglia using Gi-DREADD exerts sex-dependent anti-allodynic effects in mouse models of neuropathic pain. *Front. Pharmacol.* 11:925. 10.3389/fphar.2020.00925 32636748 PMC7318895

[B172] Sandi C. (2013). Stress and cognition. *Wiley Interdiscip. Rev. Cogn. Sci.* 4 245–261. 10.1002/wcs.1222 26304203

[B173] Sauer B. Henderson N. (1988). Site-specific DNA recombination in mammalian cells by the Cre recombinase of bacteriophage P1. *Proc. Natl. Acad. Sci. U.S.A.* 85 5166–5170. 10.1073/PNAS.85.14.5166 2839833 PMC281709

[B174] Sawicki C. M. Kim J. K. Weber M. D. Faw T. D. McKim D. B. Madalena K. M. (2019). Microglia promote increased pain behavior through enhanced inflammation in the spinal cord during repeated social defeat stress. *J. Neurosci.* 39 1139–1149. 10.1523/JNEUROSCI.2785-18.2018 30559153 PMC6381245

[B175] Schafer D. P. Lehrman E. K. Stevens B. (2013). The “Quad-partite” Synapse: Microglia-synapse interactions in the developing and mature CNS. *Glia* 61:24. 10.1002/GLIA.22389 22829357 PMC4082974

[B176] Schafer D. P. Lehrman E. K. Kautzman A. G. Koyama R. Mardinly A. R. Yamasaki R. (2012). Microglia sculpt postnatal neural circuits in an activity and complement-dependent manner. *Neuron* 74 691–705. 10.1016/J.NEURON.2012.03.026 22632727 PMC3528177

[B177] Schmidt E. E. Taylor D. S. Prigge J. R. Barnett S. Capecchi M. R. (2000). Illegitimate Cre-dependent chromosome rearrangements in transgenic mouse spermatids. *Proc. Natl. Acad. Sci. U.S.A.* 97 13702–13707. 10.1073/PNAS.240471297 11087830 PMC17639

[B178] Schmidt-Supprian M. Rajewsky K. (2007). Vagaries of conditional gene targeting. *Nat. Immunol.* 87 665–668. 10.1038/ni0707-665 17579640

[B179] Seibenhener M. L. Wooten M. C. (2015). Use of the open field maze to measure locomotor and anxiety-like behavior in mice. *J. Vis. Exp.* 96:e52434. 10.3791/52434 25742564 PMC4354627

[B180] Shi E. Shi K. Qiu S. Sheth K. N. Lawton M. T. Ducruet A. F. (2019). Chronic inflammation, cognitive impairment, and distal brain region alteration following intracerebral hemorrhage. *FASEB J.* 33 9616–9626. 10.1096/fj.201900257R 31145859

[B181] Shi Y. Manis M. Long J. Wang K. Sullivan P. M. Serrano J. R. (2019). Microglia drive APOE-dependent neurodegeneration in a tauopathy mouse model. *J. Exp. Med.* 216 2546–2561. 10.1084/JEM.20190980 31601677 PMC6829593

[B182] Shiotsuki H. Yoshimi K. Shimo Y. Funayama M. Takamatsu Y. Ikeda K. (2010). A rotarod test for evaluation of motor skill learning. *J. Neurosci. Methods* 189 180–185. 10.1016/j.jneumeth.2010.03.026 20359499

[B183] Shull M. M. Ormsby I. Kier A. B. Pawlowski S. Diebold R. J. Yin M. (1992). Targeted disruption of the mouse transforming growth factor-β1 gene results in multifocal inflammatory disease. *Nature* 359 693–699. 10.1038/359693a0 1436033 PMC3889166

[B184] Sierra A. Encinas J. M. Deudero J. J. P. Chancey J. H. Enikolopov G. Overstreet-Wadiche L. S. (2010). Microglia shape adult hippocampal neurogenesis through apoptosis-coupled phagocytosis. *Cell Stem Cell* 7 483–495. 10.1016/J.STEM.2010.08.014 20887954 PMC4008496

[B185] Sierra A. Paolicelli R. C. Kettenmann H. (2019). Cien Años de Microglía: Milestones in a century of microglial research. *Trends Neurosci.* 42 778–792. 10.1016/j.tins.2019.09.004 31635851

[B186] Sipe G. O. Lowery R. L. Tremblay M. Kelly E. A. Lamantia C. E. Majewska A. K. (2016). Microglial P2Y12 is necessary for synaptic plasticity in mouse visual cortex. *Nat. Commun.* 7 1–15. 10.1038/ncomms10905 26948129 PMC4786684

[B187] Smith B. L. Laaker C. J. Lloyd K. R. Hiltz A. R. Reyes T. M. (2020). Adolescent microglia play a role in executive function in male mice exposed to perinatal high fat diet. *Brain. Behav. Immun.* 84 80–89. 10.1016/j.bbi.2019.11.010 31765789 PMC8634520

[B188] Soch A. Sominsky L. Younesi S. De Luca S. N. Gunasekara M. Bozinovski S. (2020). The role of microglia in the second and third postnatal weeks of life in rat hippocampal development and memory. *Brain. Behav. Immun.* 88 675–687. 10.1016/j.bbi.2020.04.082 32360602

[B189] Sommer C. Schmidt C. George A. (1998). Hyperalgesia in experimental neuropathy is dependent on the TNF receptor 1. *Exp. Neurol.* 151 138–142. 10.1006/EXNR.1998.6797 9582261

[B190] Spangenberg E. Severson P. L. Hohsfield L. A. Crapser J. Zhang J. Burton E. A. (2019). Sustained microglial depletion with CSF1R inhibitor impairs parenchymal plaque development in an Alzheimer’s disease model. *Nat. Commun.* 10 1–21. 10.1038/s41467-019-11674-z 31434879 PMC6704256

[B191] Spiller K. J. Restrepo C. R. Khan T. Dominique M. A. Fang T. C. Canter R. G. (2018). Microglia-mediated recovery from ALS-relevant motor neuron degeneration in a mouse model of TDP-43 proteinopathy. *Nat. Neurosci.* 21 329–340. 10.1038/s41593-018-0083-7 29463850 PMC5857237

[B192] Spiteri A. G. Ni D. Ling Z. L. Macia L. Campbell I. L. Hofer M. J. (2022). PLX5622 reduces disease severity in lethal CNS infection by off-target inhibition of peripheral inflammatory monocyte production. *Front. Immunol.* 13:1274. 10.3389/FIMMU.2022.851556/BIBTEX PMC8990748 35401512

[B193] Squire L. R. Wixted J. T. Clark R. E. (2007). Recognition memory and the medial temporal lobe: A new perspective. *Nat. Rev. Neurosci.* 8 872–883. 10.1038/NRN2154 17948032 PMC2323975

[B194] Stanley E. R. Berg K. L. Einstein D. B. Lee P. S. W. Pixley F. J. Wang Y. (1997). Biology and action of colony-stimulating factor-1. *Mol. Reprod. Dev.* 46 4-10. 8981357 10.1002/(SICI)1098-2795(199701)46:1<4::AID-MRD2>3.0.CO;2-V

[B195] Stifter S. A. Greter M. (2020). STOP floxing around: Specificity and leakiness of inducible Cre/loxP systems. *Eur. J. Immunol.* 50 338–341. 10.1002/EJI.202048546 32125704

[B196] Strackeljan L. Baczynska E. Cangalaya C. Baidoe-Ansah D. Wlodarczyk J. Kaushik R. (2021). Microglia depletion-induced remodeling of extracellular matrix and excitatory synapses in the hippocampus of adult mice. *Cells* 10:1862. 10.3390/CELLS10081862 34440631 PMC8393852

[B197] Sweatt J. D. (2016). Neural plasticity and behavior - sixty years of conceptual advances. *J. Neurochem.* 139(Suppl. 2) 179–199. 10.1111/JNC.13580 26875778

[B198] Tahmasebi F. Pasbakhsh P. Barati S. Madadi S. Kashani I. R. (2021). The effect of microglial ablation and mesenchymal stem cell transplantation on a cuprizone-induced demyelination model. *J. Cell. Physiol.* 236 3552–3564. 10.1002/jcp.30090 32996165

[B199] Tahmasebi F. Pasbakhsh P. Mortezaee K. Madadi S. Barati S. Kashani I. R. (2019). Effect of the CSF1R inhibitor PLX3397 on remyelination of corpus callosum in a cuprizone-induced demyelination mouse model. *J. Cell. Biochem.* 120 10576–10586. 10.1002/jcb.28344 30628737

[B200] Takeuchi T. Duszkiewicz A. J. Morris R. G. M. (2014). The synaptic plasticity and memory hypothesis: Encoding, storage and persistence. *Philos. Trans. R. Soc. B Biol. Sci.* 369:20130288. 10.1098/RSTB.2013.0288 24298167 PMC3843897

[B201] Tang Y. Liu L. Xu D. Zhang W. Zhang Y. Zhou J. (2018). Interaction between astrocytic colony stimulating factor and its receptor on microglia mediates central sensitization and behavioral hypersensitivity in chronic post ischemic pain model. *Brain. Behav. Immun.* 68 248–260. 10.1016/j.bbi.2017.10.023 29080683

[B202] Tansley S. Gu N. Guzmán A. U. Cai W. Wong C. Lister K. C. (2022). Microglia-mediated degradation of perineuronal nets promotes pain. *Science.* 377 80–86. 10.1126/SCIENCE.ABL6773 35617374

[B203] Tap W. D. Wainberg Z. A. Anthony S. P. Ibrahim P. N. Zhang C. Healey J. H. (2015). Structure-guided blockade of CSF1R kinase in tenosynovial giant-cell tumor. *N. Engl. J. Med.* 373 428–437. 10.1056/NEJMOA1411366 26222558

[B204] Tay T. L. Béchade C. D’Andrea I. St-Pierre M. K. Henry M. S. Roumier A. (2018). Microglia gone rogue: Impacts on psychiatric disorders across the lifespan. *Front. Mol. Neurosci.* 10:421. 10.3389/fnmol.2017.00421 29354029 PMC5758507

[B205] Tay T. L. Savage J. C. Hui C. W. Bisht K. Tremblay M. È (2017). Microglia across the lifespan: From origin to function in brain development, plasticity and cognition. *J. Physiol.* 595 1929–1945. 10.1113/JP272134 27104646 PMC5350449

[B206] Taylor M. F. Marquis R. Coall D. Wilkinson C. (2017). Substance misuse-related parental child maltreatment: Intergenerational implications for grandparents, parents, and grandchildren relationships. *J. Drug Issues* 47 241–260. 10.1177/0022042616683670

[B207] Thiel W. A. Blume Z. I. Mitchell D. M. (2022). Compensatory engulfment and Müller glia reactivity in the absence of microglia. *Glia* 70 1402–1425. 10.1002/GLIA.24182 35451181 PMC9081278

[B208] Thomas A. Burant A. Bui N. Graham D. Yuva-Paylor L. A. Paylor R. (2009). Marble burying reflects a repetitive and perseverative behavior more than novelty-induced anxiety. *Psychopharmacology (Berl)* 204 361–373. 10.1007/s00213-009-1466-y 19189082 PMC2899706

[B209] Torres L. Danver J. Ji K. Miyauchi J. T. Chen D. Anderson M. E. (2016). Dynamic microglial modulation of spatial learning and social behavior. *Brain. Behav. Immun.* 55 6–16. 10.1016/j.bbi.2015.09.001 26348580 PMC4779430

[B210] Tremblay M. È Majewska A. K. (2011). A role for microglia in synaptic plasticity? *Commun. Integr. Biol.* 4 220–222. 10.4161/cib.4.2.14506 21655446 PMC3104585

[B211] Tremblay M. Ì Lowery R. L. Majewska A. K. (2010). Microglial interactions with synapses are modulated by visual experience. *PLoS Biol.* 8:e1000527. 10.1371/journal.pbio.1000527 21072242 PMC2970556

[B212] Tremblay M. È Stevens B. Sierra A. Wake H. Bessis A. Nimmerjahn A. (2011). The role of microglia in the healthy brain. *J. Neurosci.* 31 16064–16069. 10.1523/JNEUROSCI.4158-11.2011 22072657 PMC6633221

[B213] Unger M. S. Schernthaner P. Marschallinger J. Mrowetz H. Aigner L. (2018). Microglia prevent peripheral immune cell invasion and promote an anti-inflammatory environment in the brain of APP-PS1 transgenic mice. *J. Neuroinflammation* 15 1–23. 10.1186/s12974-018-1304-4 30241479 PMC6151006

[B214] Valdearcos M. Douglass J. D. Robblee M. M. Dorfman M. D. Stifler D. R. Bennett M. L. (2017). Microglial inflammatory signaling orchestrates the hypothalamic immune response to dietary excess and mediates obesity susceptibility. *Cell Metab.* 26 185.e–197.e. 10.1016/j.cmet.2017.05.015 28683286 PMC5569901

[B215] van der Kooij M. A. Sandi C. (2012). Social memories in rodents: Methods, mechanisms and modulation by stress. *Neurosci. Biobehav. Rev.* 36 1763–1772. 10.1016/j.neubiorev.2011.10.006 22079398

[B216] Van Hove H. Martens L. Scheyltjens I. De Vlaminck K. Pombo Antunes A. R. De Prijck S. (2019). A single-cell atlas of mouse brain macrophages reveals unique transcriptional identities shaped by ontogeny and tissue environment. *Nat. Neurosci.* 22 1021–1035. 10.1038/S41593-019-0393-4 31061494

[B217] Van Praag H. Schinder A. F. Christle B. R. Toni N. Palmer T. D. Gage F. H. (2002). Functional neurogenesis in the adult hippocampus. *Nature* 415 1030–1034. 10.1038/4151030A 11875571 PMC9284568

[B218] Van Rooijen N. Sanders A. (1996). Kupffer cell depletion by liposome-delivered drugs: Comparative activity of intracellular clodronate, propamidine, and ethylenediaminetetraacetic acid. *Hepatology* 23 1239–1243. 10.1002/hep.510230544 8621159

[B219] Van Rooijen N. Bakker J. Sanders A. (1997). Transient suppression of macrophage functions by liposome-encapsulated drugs. *Trends Biotechnol.* 15 178–185. 10.1016/S0167-7799(97)01019-6 9161052

[B220] Vanryzin J. W. Yu S. J. Perez-Pouchoulen M. McCarthy M. M. (2016). Temporary depletion of microglia during the early postnatal period induces lasting sex-dependent and sex-independent effects on behavior in rats. *eNeuro* 3 1–19. 10.1523/ENEURO.0297-16.2016 27957532 PMC5144556

[B221] Vichaya E. G. Malik S. Sominsky L. Ford B. G. Spencer S. J. Dantzer R. (2020). Microglia depletion fails to abrogate inflammation-induced sickness in mice and rats. *J. Neuroinflammation* 17 1–14. 10.1186/s12974-020-01832-2 32475344 PMC7262755

[B222] Villa A. Gelosa P. Castiglioni L. Cimino M. Rizzi N. Pepe G. (2018). Sex-specific features of microglia from adult mice. *Cell Rep.* 23 3501–3511. 10.1016/j.celrep.2018.05.048 29924994 PMC6024879

[B223] Vincenzi M. Milella M. S. D’Ottavio G. Caprioli D. Reverte I. Maftei D. (2022). Targeting chemokines and chemokine GPCRs to enhance strong opioid efficacy in neuropathic pain. *Life* 12:398. 10.3390/life12030398 35330149 PMC8955776

[B224] Vorhees C. V. Williams M. T. (2006). Morris water maze: Procedures for assessing spatial and related forms of learning and memory. *Nat. Protoc.* 1 848–858. 10.1038/nprot.2006.116 17406317 PMC2895266

[B225] Wake H. Moorhouse A. J. Jinno S. Kohsaka S. Nabekura J. (2009). Resting microglia directly monitor the functional state of synapses in vivo and determine the fate of ischemic terminals. *J. Neurosci.* 29 3974–3980. 10.1523/JNEUROSCI.4363-08.2009 19339593 PMC6665392

[B226] Walf A. A. Frye C. A. (2007). The use of the elevated plus maze as an assay of anxiety-related behavior in rodents. *Nat. Protoc.* 2 322–328. 10.1038/nprot.2007.44 17406592 PMC3623971

[B227] Wallace J. Lord J. Dissing-Olesen L. Stevens B. Murthy V. N. (2020). Microglial depletion disrupts normal functional development of adult-born neurons in the olfactory bulb. *Elife* 9 1–30. 10.7554/eLife.50531 32150529 PMC7062469

[B228] Wang C. Wang L. Gu Y. (2021). Microglia, synaptic dynamics and forgetting. *Brain Res. Bull.* 174 173–183. 10.1016/J.BRAINRESBULL.2021.06.005 34129917

[B229] Wang C. Yue H. Hu Z. Shen Y. Ma J. Li J. (2020). Microglia mediate forgetting via complement-dependent synaptic elimination. *Science* 367 688–694. 10.1126/science.aaz2288 32029629

[B230] Wang J. Lai S. Li G. Zhou T. Wang B. Cao F. (2020). Microglial activation contributes to depressive-like behavior in dopamine D3 receptor knockout mice. *Brain. Behav. Immun.* 83 226–238. 10.1016/j.bbi.2019.10.016 31626970

[B231] Wang X. Zhao L. Zhang J. Fariss R. N. Ma W. Kretschmer F. (2016). Requirement for microglia for the maintenance of synaptic function and integrity in the mature retina. *J. Neurosci.* 36 2827–2842. 10.1523/JNEUROSCI.3575-15.2016 26937019 PMC4879218

[B232] Wang Y. R. Y. X. Mao X. F. Wu H. Y. Wang Y. R. Y. X. (2018). Liposome-encapsulated clodronate specifically depletes spinal microglia and reduces initial neuropathic pain. *Biochem. Biophys. Res. Commun.* 499 499–505. 10.1016/j.bbrc.2018.03.177 29596830

[B233] Wang Y. Huang Z. (2020). Microglia interact with neurons by forming somatic junctions. *Neurosci. Bull.* 36 1085–1088. 10.1007/s12264-020-00517-3 32449123 PMC7475154

[B234] Warden A. S. Triplett T. A. Lyu A. Grantham E. K. Azzam M. M. DaCosta A. (2021). Microglia depletion and alcohol: Transcriptome and behavioral profiles. *Addict. Biol.* 26:e12889. 10.1111/adb.12889 32176824 PMC8510547

[B235] Weber M. D. McKim D. B. Niraula A. Witcher K. G. Yin W. Sobol C. G. (2019). The influence of microglial elimination and repopulation on stress sensitization induced by repeated social defeat. *Biol. Psychiatry* 85 667–678. 10.1016/j.biopsych.2018.10.009 30527629 PMC6440809

[B236] Weinhard L. Di Bartolomei G. Bolasco G. Machado P. Schieber N. L. Neniskyte U. (2018). Microglia remodel synapses by presynaptic trogocytosis and spine head filopodia induction. *Nat. Commun.* 9:1228. 10.1038/s41467-018-03566-5 29581545 PMC5964317

[B237] Werneburg S. Feinberg P. A. Johnson K. M. Schafer D. P. (2017). A microglia-cytokine axis to modulate synaptic connectivity and function. *Curr. Opin. Neurobiol.* 47 138–145. 10.1016/j.conb.2017.10.002 29096242 PMC5797987

[B238] Willis E. F. MacDonald K. P. A. Nguyen Q. H. Garrido A. L. Gillespie E. R. Harley S. B. R. (2020). Repopulating microglia promote brain repair in an IL-6-dependent manner. *Cell* 180 833.e–846.e. 10.1016/j.cell.2020.02.013 32142677

[B239] Witcher K. G. Bray C. E. Chunchai T. Zhao F. O’Neil S. M. Gordillo A. J. (2021). Traumatic brain injury causes chronic cortical inflammation and neuronal dysfunction mediated by Microglia. *J. Neurosci.* 41 1597–1616. 10.1523/JNEUROSCI.2469-20.2020 33452227 PMC7896020

[B240] Worthen R. J. Garzon Zighelboim S. S. Torres Jaramillo C. S. Beurel E. (2020). Anti-inflammatory IL-10 administration rescues depression-associated learning and memory deficits in mice. *J. Neuroinflammation* 17 1–16. 10.1186/s12974-020-01922-1 32828124 PMC7443292

[B241] Wu W. Li Y. Wei Y. Bosco D. B. Xie M. Zhao M. G. (2020). Microglial depletion aggravates the severity of acute and chronic seizures in mice. *Brain. Behav. Immun.* 89 245–255. 10.1016/j.bbi.2020.06.028 32621847 PMC7572576

[B242] Wyatt-Johnson S. K. Sommer A. L. Shim K. Y. Brewster A. L. (2021). Suppression of microgliosis with the colony-stimulating factor 1 receptor inhibitor PLX3397 does not attenuate memory defects during epileptogenesis in the rat. *Front. Neurol.* 12:651096. 10.3389/fneur.2021.651096 34149593 PMC8209304

[B243] Xie S. T. Chen A. X. Song B. Fan J. Li W. Xing Z. (2020). Suppression of microglial activation and monocyte infiltration ameliorates cerebellar hemorrhage induced-brain injury and ataxia. *Brain. Behav. Immun.* 89 400–413. 10.1016/j.bbi.2020.07.027 32717406

[B244] Xu T. Yu X. Perlik A. J. Tobin W. F. Zweig J. A. Tennant K. (2009). Rapid formation and selective stabilization of synapses for enduring motor memories. *Nature* 462 915–919. 10.1038/nature08389 19946267 PMC2844762

[B245] Yamamoto M. Kim M. Imai H. Itakura Y. Ohtsuki G. (2019). Microglia-triggered plasticity of intrinsic excitability modulates psychomotor behaviors in acute cerebellar inflammation. *Cell Rep.* 28 2923.e–2938.e. 10.1016/j.celrep.2019.07.078 31509752

[B246] Yang X. Ren H. Wood K. Li M. Qiu S. Shi F. D. (2018). Depletion of microglia augments the dopaminergic neurotoxicity of MPTP. *FASEB J.* 32 3336–3345. 10.1096/fj.201700833RR 29401614 PMC5956250

[B247] Yang Y. Ishima T. Wan X. Wei Y. Chang L. Zhang J. (2021). Microglial depletion and abnormalities in gut microbiota composition and short-chain fatty acids in mice after repeated administration of colony stimulating factor 1 receptor inhibitor PLX5622. *Eur. Arch. Psychiatry Clin. Neurosci.* 272 483–495. 10.1007/s00406-021-01325-0 34480631

[B248] Yao Y. Echeverry S. Shi X. Q. Yang M. Yang Q. Z. Wang G. Y. F. (2016). Dynamics of spinal microglia repopulation following an acute depletion. *Nat. Publ. Gr.* 6 1–12. 10.1038/srep22839 26961247 PMC4785356

[B249] Yegla B. Boles J. Kumar A. Foster T. C. (2021). Partial microglial depletion is associated with impaired hippocampal synaptic and cognitive function in young and aged rats. *Glia* 69 1494–1514. 10.1002/glia.23975 33586813 PMC8278544

[B250] Yi M. H. Liu Y. U. Liu K. Chen T. Bosco D. B. Zheng J. (2021). Chemogenetic manipulation of microglia inhibits neuroinflammation and neuropathic pain in mice. *Brain. Behav. Immun.* 92 78–89. 10.1016/j.bbi.2020.11.030 33221486 PMC7897256

[B251] Yirmiya R. Goshen I. (2011). Immune modulation of learning, memory, neural plasticity and neurogenesis. *Brain. Behav. Immun.* 25 181–213. 10.1016/j.bbi.2010.10.015 20970492

[B252] Zhan L. Krabbe G. Du F. Jones I. Reichert M. C. Telpoukhovskaia M. (2019). Proximal recolonization by self-renewing microglia re-establishes microglial homeostasis in the adult mouse brain. *PLoS Bio.* 17:e3000134. 10.1371/journal.pbio.3000134 30735499 PMC6383943

[B253] Zhan Y. Paolicelli R. C. Sforazzini F. Weinhard L. Bolasco G. Pagani F. (2014). Deficient neuron-microglia signaling results in impaired functional brain connectivity and social behavior. *Nat. Neurosci.* 17 400–406. 10.1038/nn.3641 24487234

[B254] Zhang D. Li S. Hou L. Jing L. Ruan Z. Peng B. (2021). Microglial activation contributes to cognitive impairments in rotenone-induced mouse Parkinson’s disease model. *J. Neuroinflammation* 18 1–16. 10.1186/s12974-020-02065-z 33402167 PMC7786472

[B255] Zhao X. F. Alam M. M. Liao Y. Huang T. Mathur R. Zhu X. (2019). Targeting microglia using Cx3cr1-Cre Lines: Revisiting the specificity. *eNeuro* 6 1–11. 10.1523/ENEURO.0114-19.2019 31201215 PMC6620394

[B256] Zhou K. Han J. Lund H. Boggavarapu N. R. Lauschke V. M. Goto S. (2022). An overlooked subset of Cx3cr1wt/wt microglia in the Cx3cr1CreER-Eyfp/wt mouse has a repopulation advantage over Cx3cr1CreER-Eyfp/wt microglia following microglial depletion. *J. Neuroinflammation* 19 1–18. 10.1186/s12974-022-02381-6 35062962 PMC8783445

[B257] Zhou L. J. Peng J. Xu Y. N. Zeng W. J. Zhang J. Wei X. (2019). Microglia are indispensable for synaptic plasticity in the spinal dorsal horn and chronic pain. *Cell Rep.* 27 3844.e–3859.e. 10.1016/j.celrep.2019.05.087 31242418 PMC7060767

[B258] Zhu H. Aryal D. K. Olsen R. H. J. Urban D. J. Swearingen A. Forbes S. (2016). Cre-dependent DREADD (Designer Receptors Exclusively Activated by Designer Drugs) mice. *Genesis* 54 439–446. 10.1002/dvg.22949 27194399 PMC4990490

