# Supplementary material for: What microglia depletion approaches tell us about the role of microglia on synaptic function and behavior
Source: Front Cell Neurosci. 2022 Nov 4;16:1022431. doi: 10.3389/fncel.2022.1022431 (PMC9673171; doi:10.3389/fncel.2022.1022431)
Supplement: Supplementary file 1 [file Data_Sheet_1.pdf]

SUPPLEMENTARY ONLINE MATERIALS

What microglia depletion approaches tell us about the role of microglia on synaptic function and behavior

Bernadette Basilico[1], Laura Ferrucci[2], Azka Khan[2], Silvia di Angelantonio[2,3], Davide Ragozzino[4,5*], Ingrid Reverte[2,5*]

[1]Institute of Science and Technology (ISTA) Austria, Klosterneuburg, Austria
[2]Department of Physiology and Pharmacology, Sapienza University of Rome, Rome, Italy
[3]Center for Life Nano- & Neuro- science, Istituto Italiano di Tecnologia, Rome, Italy
[4]Laboratory affiliated to Institute Pasteur Italia – Fondazione Cenci Bolognetti – Department of Physiology and Pharmacology, Sapienza University of Rome, Rome, Italy
[5]Santa Lucia Foundation (IRCCS Fondazione Santa Lucia), Rome, Italy

*Corresponding authors: Davide Ragozzino (davide.ragozzino@uniroma1.it); Ingrid Reverte (ingrid.reverte@uniroma1.it)

Supplementary Table 1. Effects of microglia depletion during adulthood on Spontaneous Locomotor Activity and Anxiety in healthy rodents.

Behavioral task	Animal model	Animals age	Depletion model	Depletion duration	Microglia depletion effects	Reference
Open Field	C57BL/6 male mice	2 months old	Bilateral injection of clodronate (10 mg/ml) in hippocampal CA1	5 days recovery	No effect	(Torres et al., 2016)
Open Field	C57BL/6 male mice	2 months old	PLX3397 290 mg/kg chow	3 weeks	No effect	(Torres et al., 2016)
Open Field	C57BL/6 male mice	2 months old	PLX3397 290 mg/kg chow	1 week + 2 weeks withdrawal	No effect	(Torres et al., 2016)
Open Field	C57BL/6 male and female mice	2 months old	PLX3397 290 mg/kg chow	3 weeks + 3 weeks withdrawal	No effect	(Elmore et al., 2015)
Open Field	$Cx3cr1^{GFP/+}$ male mice	Post-natal day 45	PLX3397 290 mg/kg chow	3 weeks (P21-P45)	No effect*	(Cao et al., 2021)
Open Field and Elevated Plus Maze	CaM/Tet-DTA male and female mice	5-8 months old	PLX3397 290 mg/kg chow	4 weeks	No effect*	(Rice et al., 2015)
Elevated Plus Maze	C57BL/6 male mice	2 months old	PLX3397 290 mg/kg chow	3 or 8 weeks	Increased time spent in closed arms (3 weeks)<br><br>No effect (8 weeks)	(Elmore et al., 2014)
Baseline locomotor activity	C57BL/6 male mice	2 months old	PLX3397 290 mg/kg chow	2 weeks	No effect*	(Wu et al., 2020)
Open Field	C57BL6/J male	2 months old	PLX5622 1200 mg/kg chow	1 week	No effect	(Basilico et al., 2021)
Open Field	C57BL/6 male mice	3 and 22 months old	PLX5622 1200 mg/kg chow	2 weeks + 4 week withdrawal	Increased exploration in microglia repopulated mice	(Elmore et al., 2018)
Open Field	C57BL6/J male mice	3 months old	PLX5622 1200 mg/kg chow	4 weeks	No effect*	(Vichaya et al., 2020)
Open Field	WT mice	2 months old	PLX5622 300 or 1200 mg/kg chow	2 weeks	No effect*	(Dagher et al., 2015)
Open Field	C57BL/6 male mice	2 months old	PLX5622 1200 mg/kg chow	2 weeks	No effect*	(Weber et al., 2019)
Open Field and Elevated Plus Maze	WT male and female	7 months old	PLX5622 1200 mg/kg chow	24 weeks	No effect*	(Spangenberg et al., 2019)
Open Field and Elevated Plus Maze	C57BL/6 male and female mice	3 months old	PLX3397 290 mg/kg chow	5 weeks	No effect	(Wang et al., 2020)
Elevated Plus Maze	$Cx3cr1^{GFP/+}$ male and female mice	2 months old	PLX5622 1200 mg/kg chow	8 weeks	No effect	(Ayata et al., 2018)
Elevated Plus Maze	C57BL/6 male and female mice	2-4 months old	PLX5622 1200 mg/kg chow	3 weeks	No effect	(Badimon et al., 2020a)
Elevated Plus Maze	C57BL6/J male mice	6 months old	PLX5622 1200 mg/kg chow	3-4 weeks	No effect*	(Allen et al., 2020)
Open Field and Marble burying test	B6/129 F1 male mice	2 months old	PLX5622 1200 mg/kg chow	1 week	No effect*	(Adeluyi et al., 2019)
Light-dark test	C57BL/6N male mice	2 months old	PLX5622 1200 mg/kg chow	4 weeks	No effect*	(Lehmann et al., 2019)

Home-cage voluntary running wheel	C57BL6/J male mice	3 months old	PLX5622 1200 mg/kg chow	4 weeks	Decreased voluntary activity	(Vichaya et al., 2020)
Open Field	C57BL/6 male mice 2 mo	2 months old	Ki20227 20 mg/kg (i.g.)	2 weeks	No effect*	(Xie et al., 2020)
Open Field	$Cx3cr1^{DTR}$ male rats	3 months old	DT 25 ng/g (s.c.) twice/day	2 or 24h recovery	No effect*	(Vichaya et al., 2020)
Elevated Plus Maze and Light-dark test	$Cx3cr1^{DTR}$ male rats	3 months old	DT 25 ng/g (s.c.) twice/day	2 or 24h recovery	No effect	(De Luca et al., 2019)

Diphtheria toxin (DT); intragastrical administration (i.g.); postnatal-day (P); subcutaneous injection (s.c.); week wild-type (WT). *The data refers to control mice with microglia depletion, extracted from studies using animal models of brain pathologies.

Supplementary Table 2. Effects of microglia depletion during adulthood on Sensory Perception in healthy rodents.

Sensory test	Animal model	Animal age	Depletion model	Depletion duration	Microglia depletion effects	Reference
Visual acuity	$CX3CR1^{CRER}$: DTA male mice	2-3 months old	Tamoxifen 500 mg/kg (i.g.)	4 weeks	No effect	(Wang et al., 2016)
Taste perception	$Cx3cr1^{DTR}$ male rats	3 months old	DT 25 ng/g (s.c.) twice/day	48h recovery	Distaste for palatable foods	(De Luca et al., 2019)
Mechanical nociception	C57BL/6 male mice	2 months old	PLX5622 1200 mg/kg chow	2 weeks	No effect*	(Sawicki et al., 2019)
Mechanical and thermal nociception	C57BL/6CR male mice	3 months old	PLX3397 290 mg/kg chow	7 weeks	No effect*	(Liang et al., 2019)
Olfaction test	C57BL/6 male and female mice	2-4 months old	PLX5622 1200 mg/kg chow	3 weeks	No effect	(Badimon et al., 2020a)

Diphtheria toxin (DT); intragastrical administration (i.g.); subcutaneous injection (s.c.). *The data refers to control mice with microglia depletion, extracted from studies using animal models of brain pathologies.

Supplementary Table 3. Effects of microglia depletion during adulthood on Motor Performance and Learning in healthy rodents.

Behavioral task	Animal model	Animal age	Depletion model	Depletion duration	Microglia depletion effects	Reference
Pole test	C57BL/6L male mice	2 months old	PLX3397 40 mg/kg/day (i.p.)	3 weeks	No effect*	(Yang et al., 2018)
Grip strength test	C57BL/6 male and female mice	6-11 months old	PLX3397 275 mg/kg chow	5 weeks	No effect*	(Crapser et al., 2020a)
Balance beam	C57BL6/J-129/SvEv male and female mice	3 months old	PLX3397 200 mg/kg chow	8 weeks	No effect*	(Qu et al., 2017)
Gait test	C57BL/6 male mice	3 months old	PLX5622 1200 mg/kg chow	9 weeks	No effect*	(Li et al., 2020)
Bar test/ Hurdle test	C57BL6/N male and female mice	2 months old	PLX5622 1200 mg/kg chow	10 weeks + 5 weeks withdrawal	No effect*	(Garcia-Agudo et al., 2019)
Beam walk	C57BL/6J male mice	3 months old	PLX5622 1200 mg/kg chow	1 week + 0-12 weeks withdrawal	No effect*	(Henry et al., 2020)
Gait test	C57BL/6 male mice	2 months old	Ki20227 20 mg/kg (i.g.)	2 weeks	No effect*	(Xie et al., 2020)
Ataxia scoring	CX3CR1-CreER^{T2}:iDTR	adult mice	Tamoxifen 10 µg/mouse (i.p.) 5 days + 1 µg/mouse DT (i.p.) three days	3 days + 5-25 days recovery	Signs of ataxia	(Rubino et al., 2018)
Rotarod 8-40 r.p.m.; 5 min; 5 trials x 1 day	C57BL/6 male mice	2 months old	PLX3397 290 mg/kg chow	3 or 8 weeks	No effect	(Elmore et al., 2014)
Rotarod 0-40 r.p.m.; 15 min; 3 trials x 1 day	C57BL/6L male mice	2 months old	PLX3397 40 mg/kg/day (i.p.)	3 weeks	No effect*	(Yang et al., 2018)
Rotarod 0-40 r.p.m.; 5 min; 3 trials x 1 day	C57BL/6 male and female mice	6-11 months old	PLX3397 275 mg/kg chow	5 weeks	No effect*	(Crapser et al., 2020a)
Rotarod 4-40 r.p.m.; 5 min; 1 trial x 4 days	C57BL/6CR male mice 3 mo	3 months old	PLX3397 290 mg/kg chow	7 weeks	No effect*	(Liang et al., 2019)
Rotarod 4-40 r.p.m.; 5 min; 4 trials x 4 days	C57BL6/J-129/SvEv male and female mice	3 months old	PLX3397 200 mg/kg chow	8 weeks	No effect*	(Qu et al., 2017)

	Rotarod 8-40 r.p.m.; 5 min; 5 trials x 1 day	C57BL/6 male and female mice \	2 months old	PLX3397 290 mg/kg chow	3 weeks + 2 week withdrawal	No effect	(Elmore et al., 2015)
	Rotarod 4-40 r.p.m.; x 5 min; 3 trials x 3 day	C57BL/6 male and female mice	2-4 months old	PLX5622 1200 mg/kg chow	3 weeks	No effect	(Badimon et al., 2020b)
	Rotarod 8-40 r.p.m.; 5 min; 5 trials x 1 day	C57BL/6 male mice	3-22 months old	PLX5622 1200 mg/kg chow	2 weeks + 4 week withdrawal	No effect	(Elmore et al., 2018)
	Rotarod 5:50 r.p.m.; 5 min; 6 trials x 1 day	C57BL/6 male mice	2 months old	Ki20227 20 mg/kg (i.g.)	2 weeks	No effect*	(Xie et al., 2020)
	Rotarod 0-100 r.p.m.; 3 min; 20-trial x 3 sessions x 2 days	Cx3cr1^{CreER}:R2 6 ^{iDTR} mice	1-2 months old	Tamoxifen 50 µg or 10mg/mouse (i.g.) twice + 1 µg/mouse DT (i.p) 3 days (30d later)	3 days + 48h recovery	Lesser performance improvement	(Parkhurst et al., 2013)
	Rotarod 4-40 r.p.m.; 5 min; 1 trial x 1 session	Cx3cr1-CreER^{T2}:iDTR	adult mice	Tamoxifen 10 ug/mouse i.p. 5 days + 1 µg/mouse DT (i.p.) 3 days	3 days + 10 days recovery	Loss of motor coordination	(Rubino et al., 2018)

Diphtheria toxin (DT); intragastrical administration (i.g.); intraperitoneal injection (i.p.); revolutions per minute (r.p.m.); subcutaneous injection (s.c.). *The data refers to control mice with microglia depletion, extracted from studies using animal models of brain pathologies.

Supplementary Table 4. Effects of microglia depletion during adulthood on Sociability and Social Recognition Memory in healthy rodents.

Behavioral task	Animal model	Animal age	Depletion model	Depletion duration	Microglia depletion effects	Reference
Three-chamber social interaction test	C57BL/6 male and female mice	2-4 months old	PLX5622 1200 mg/kg chow	3 weeks	No effect	(Badimon et al., 2020a)
Three-chamber social interaction test	C57BL/6 male mice	2 months old	PLX3397 290 mg/kg chow	3 weeks	No effect	(Torres et al., 2016)
Three-chamber social interaction test	C57BL/6 male mice	2 months old	Bilateral injection of clodronate (10 mg/ml) in hippocampal CA1	2 days recovery	Impaired social preference and recognition.<br><br>The effect reversed upon microglia repopulation	(Torres et al., 2016)
Social interaction with an unfamiliar CD-1 mouse	C57BL/6N male mice	2 months old	PLX5622 1200 mg/kg chow	4 weeks	No effect*	(Lehmann et al., 2019)
Social interaction with an unfamiliar CD-1 mouse	C57BL/6J male mice	2 months old	PLX3397 290 mg/kg chow	2 weeks + 2 weeks withdrawal	No effect*	(Gu et al., 2021)

*The data refers to control mice with microglia depletion, extracted from studies using animal models of brain pathologies.

Supplementary Table 5. Effects of microglia depletion during adulthood on Recognition Learning and Memory in healthy rodents.

Behavioral task	Animal model	Animal age	Depletion model	Depletion duration	Microglia depletion effects	Reference
Novel Object Recognition<br>1 x 15 min Habituation<br>1 x 15 min Familiarization<br>1 x 15 min Test (24h later)	WT male and female mice	Post-natal day 22	PLX3397 445 mg/kg chow	1 week (P16-22)	Lower discrimination index (non-significant trend) *	(Pinto et al., 2020)
Novel Object Location<br>1 x 15 min Habituation<br>1 x 15 min Familiarization<br>1 x 15 min Test (24h later)	WT male and female mice	Post-natal day 22	PLX3397 445 mg/kg chow	1 week (P16-22)	No effect*	(Pinto et al., 2020)
Novel Object Recognition<br>1 x 20 min Habituation<br>1 x 5 min Familiarization<br>1x 5 min Test (2h later)	Spragwe-Dawley male rats	2 months old	PLX3397 455mg/kg chow	2 weeks	No effect*	(Wyatt-Johnson et al., 2021)
Novel Object Recognition<br>1 x 5 min Familiarization<br>1 x 5 min Test (24h later)	C57BL/6 male and female mice	6-11 months old	PLX3397 275 mg/kg chow	5 weeks	No effect*	(Crapser et al., 2020b)
Novel Object Recognition<br>3 x 10 min Habituation<br>1 x 10 min Familiarization<br>1 x 10 min Test (6h later)	C57BL6/J male mice	3 months old	PLX3397 290 mg/kg chow	7 weeks	No effect*	(Kakae et al., 2019)
Novel Object Recognition<br>1 x 5 min Familiarization<br>1 x 5 min Test (5 min later)	C57BL/6 male mice	2 months old	PLX5622 1200 mg/kg chow	1 week	No effect*	(Worthen et al., 2020)
Novel Object Recognition<br>2 x 10 min Habituation<br>3 x 10 min x 3 days Familiarization<br>1 x 5 min Test (1h later)	C57BL6/J male mice	2 months old	PLX5622 1200 mg/kg chow	1 week	Increased object exploration during familiarization<br><br>Lower discrimination index<br><br>Recovered upon microglia repopulation	(Basilico et al., 2019)
Novel Object Recognition / Novel Object Location<br>1x 10 min Habituation<br>1 x 10 min Familiarization<br>1 x 10 min Test Novel Location (24h later)<br>1 x 10 min Test Novel Object (24h later)	C57BL/6 male mice	2 months old	PLX5622 1200 mg/kg chow	2 weeks	No effect*	(Witcher et al., 2021)
Novel Object Recognition[1]<br>3 x 10 min Habituation<br>1 x 5 min Familiarization<br>1 x 5 min Test (5 min later)	C57BL6/J male mice	6 months old	PLX5622 1200 mg/kg chow	3-4 weeks	No effect*	(Allen et al., 2020)

Novel Object Location[1]<br>2 x 10 min Habituation<br>1 x 5 min Familiarization<br>1 x 5 min Test (5 min later)	C57BL6/J male mice	6 months old	PLX5622 1200 mg/kg chow	3-4 weeks	No effect*	(Allen et al., 2020)
Novel Object Recognition[1]<br>3 x 10 min Habituation<br>1 x 5 min Familiarization<br>1 x 5 min Test (5 min later)	C57BL6/J male mice	6 months old	PLX5622 1200 mg/kg chow	4 weeks	No effect*	(Acharya et al., 2016)
Novel Object Location[1]<br>2 x 10 min Habituation<br>1 x 5 min Familiarization<br>1 x 5 min Test (5 min later)	C57BL6/J male mice	6 months old	PLX5622 1200 mg/kg chow	4 weeks	No effect*	(Acharya et al., 2016)
Novel Object Recognition<br>2 x 20 min Habituation<br>1 x 5 min Familiarization<br>1 x 10 min Test (5 min later)	BL6/CD1 male and female mice	2 months old	PLX5622 1200 mg/kg chow	4 weeks	No effect*	(Gibson et al., 2019)
Novel Object Recognition<br>2 x 10 min Habituation<br>1 x 5 min Familiarization<br>1 x 5 min Test (24h later)	C57BL6/J male mice	2 months old	PLX5622 300 mg/kg chow	3 weeks + 4 week withdrawal	No effect*	(Feng et al., 2016)
Novel Object Recognition<br>1 x at least 20 sec exploring objects Familiarization<br>1 x Test (24h later)	C57BL/6J male mice	3 months old	PLX5622 1200 mg/kg chow	1 week + 0-12 week withdrawal	No effect*	(Henry et al., 2020)
Novel Object Recognition<br>1 x 5 min Familiarization<br>1 x 3 min Test (24h later)	C57BL/6 male and female mice	2 months old	PLX3397 290 mg/kg chow	3 weeks + 2 week withdrawal	No effect	(Elmore et al., 2015)
Novel Object Recognition<br>1 x 3 min Familiarization<br>1 x 3 min Test (1h later)	$Cx3cr1^{DTR}$ male rats	3 months old	25 ng/g DT (s.c.) twice/day	48h or 1 week recovery	No effect	(De Luca et al., 2020)
Novel Object Recognition<br>1 x 30 min Habituation<br>1 x 10 min Familiarization<br>1 x 5 min Test (1h later)	$CX3CR1^{CreER}$:$R26^{iDTR}$ mice	1-2 months old	Tamoxifen 50 µg or 10mg/mouse (i.g.) twice + 1 µg/mouse DT (i.p) x three days (30d later)	3 days + 48h recovery	Lower discrimination index	(Parkhurst et al., 2013)
Context-Object recognition task<br>2 x 10 min Familiarization Context A<br>2 x 10 min Familiarization Context B<br>1 x 5 min Test (24h later)	male Fischer-344 rats	5 or 24 months old	45 mg/kg of PLX3397 chow	3 weeks	Increased object exploration during familiarization.<br><br>Lower discrimination index.<br><br>Recovered upon microglia repopulation	(Yegla et al., 2021)

Diphtheria toxin (DT); intragastrical administration (i.g.); intraperitoneal injection (i.p.); post-natal day (P); subcutaneous injection (s.c.); wild-type (WT). Habituation sessions - mice are permitted to explore the empty apparatus; Familiarization sessions - mice are allowed to explore a set of objects (typically a pair); Test – one of the objects (Novel Object Recognition) or the object location (Novel Object Location) is changed – the test is performed at different retention intervals to assess short or long-term memory. *The data refers to control mice with microglia depletion, extracted from studies using animal models of brain pathologies. [1]Methods were retrieved from Parihar et al. (2015) since the authors refer to previous studies for the description of behavioral procedures.

Supplementary Table 6. Effects of microglia depletion during adulthood on Associative Memory in healthy rodents.

Behavioral task	Animal model	Animal age	Depletion model	Depletion duration	Microglia depletion effects	Reference
Contextual Fear Conditioning<br>Conditioning: 3d x 3 US trials<br>Test: 5 min (5d and 35d later)<br>*(US 2s-shock 0.5 mA; ITI 60)*	C57BL/6 male and female mice	3 months old	PLX3397 290 mg/kg chow	5 days	No effect	(Wang et al., 2020)
Contextual Fear Conditioning<br>Conditioning 1 US trial<br>Test: 2 min (24h later)<br>*(US 3s-shock 0.5 mA)*	C57BL/6 male mice	2 months old	PLX3397 290 mg/kg chow	3 or 8 weeks	No effect	(Elmore et al., 2014)
Contextual Fear Conditioning<br>Conditioning: 1 US trial<br>Test: 5 min (24h later)<br>*(US 3s shock 0.2 mA)*	WT male and female mice	7 months old	PLX5622 1200 mg/kg chow	24 weeks	No effect*	(Spangenberg et al., 2019)
Contextual Fear Conditioning<br>Conditioning: 3d x 3 US trials<br>Test: 5 min (5d and 35d later)<br>*(US 2s-shock 0.5 mA; ITI 60)*	$CD11b^{DTR}$ male and female	3 months old	DT 20 ng/kg/day (i.c.v.)	35 days (after conditioning)	More freezing in test on day 35	(Wang et al., 2020)
Contextual Fear Conditioning<br>Conditioning: 3d x 3 US trials<br>Test: 5 min (5d and 35d later)<br>*(US 2s-shock 0.5 mA; ITI 60)*	C57BL/6 male and female mice	3 months old	PLX3397 290 mg/kg chow	5 or 35 days (after conditioning)	More freezing in test on day 35	(Wang et al., 2020)
Contextual Fear Conditioning<br>/Auditory Fear Conditioning[1]<br>Conditioning (CxA): 5 CS-US trials<br>Test (CxA): 5min (24h after)<br>Test (CxB): 1 min pre-CS, 3-min CS (1h later)<br>*(CS 30s-tone; US 1s- shock 1 mA)*	C57BL6/J male mice	6 months old	PLX5622 1200 mg/kg chow	4 weeks	No effect*	(Acharya et al., 2016)
Contextual Fear Conditioning<br>/Auditory Fear Conditioning<br>Conditioning: 5 x CS-US trials<br>Test: 5 min (72h later)<br>*(CS 20s-tone; US 1s-shock 0.8 mA; ITI 120s)*	C57BL6/J male mice	2 months old	PLX5622 300 mg/kg chow	1 week	No effect*	(Feng et al., 2016)
Contextual Fear Conditioning<br>/Auditory Fear Conditioning<br>Conditioning (CxA): 2 CS-US trials<br>Test (CxA): 5 min (24h later)<br>Test (CxB): 3 min pre-CS, 3-min CS (2h later)<br>Extinction (CxB): 5d x 3 min pre-CS 3-min CS<br>*(CS 15 sec-tone; US 2s-shock 0.6-mA; ITI 180s)*	Fischer-344 male rats	5 or 24 months old	PLX3397 45 mg/kg chow	3 weeks + 2 weeks withdrawal	Increased freezing before and during the CS and delayed extinction in young depleted rats<br><br>Reduced context- and CS- associated freezing in aged microglia depleted and repopulated rats<br><br>Improved extinction in aged microglia depleted and repopulated rats	(Yegla et al., 2021)

Auditory Fear Conditioning<br>Conditioning: 3 CS-US trials<br>Extinction: 3d x 20 CS trials<br>*(CS 120s tone; US 1s-shock 1 mA; ITI 120s)*	C57BL6/J male mice	6 months old	PLX5622 1200 mg/kg chow	3-4 weeks	Delayed extinction	(Allen et al., 2020)
Auditory Fear Conditioning<br>Conditioning (CxA) 3 CS-US trials<br>Test (CxB): 2 min pre-CS, 2-min CS<br>*(CS 30s tone; US 2s-shock 0.5-mA; ITI 15s)*	$Cx3cr1^{CreER}$:$R26^{iDTR}$ mice	1-2 months old	Tamoxifen 50 µg or 10mg/mouse (i.g.) twice + DT 1 µg/mouse (i.p) 3 days	3 days + 48h recovery	Reduced freezing during the CS presentation	(Parkhurst et al., 2013)

Conditioned stimulus (CS); context (Cx); days (d); diphtheria toxin (DT); intragastrical administration (i.g.); intracerebroventricular injection (i.c.v.); intraperitoneal injection (i.p.); inter-trial interval (ITI); milliamperes (mA); post-natal day (P); unconditioned stimulus (US); wild-type (WT).*The data refers to control mice with microglia depletion, extracted from studies using animal models of brain pathologies. [1]Methods were retrieved from Christie et al. (2012) since the authors refer to previous studies for the exact behavioral procedures.

Supplementary Table 7. Effects of microglia depletion during adulthood on Spatial Learning and Memory in healthy rodents.

Behavioral task	Animal model	Animal age	Depletion model	Depletion duration	Microglia depletion effects	Reference
Barnes Maze	C57BL/6 male mice	2 months old	Bilateral injection of clodronate (10 mg/ml) in hippocampal CA1	20 days recovery	Worsened acquisition<br><br>No effect after microglia repopulation	(Torres et al., 2016)
Barnes Maze	C57BL/6 male mice	2 months old	PLX3397 290 mg/kg chow	1 or 3 weeks	Worsened acquisition in mice treated for 1 week, but not 3 weeks	(Torres et al., 2016)
Barnes Maze	Spragwe-Dawley male rats	2 months old	PLX3397 455mg/kg chow	2 weeks	No effect*	(Wyatt-Johnson et al., 2021)
Barnes Maze	C57BL/6 male mice	2 months old	PLX3397 290 mg/kg chow	3 weeks	No effect	(Elmore et al., 2014)
Barnes Maze	C57BL/6 male mice	2 months old	PLX3397 290 mg/kg chow	8 weeks	Improved acquisition<br><br>No effect in probe trial	(Elmore et al., 2014)
Barnes Maze	C57BL/6 male mice	2 months old	PLX3397 290 mg/kg chow	1 week + 2 week withdrawal	No effect	(Torres et al., 2016)
Barnes Maze	WT mice	2 months old	PLX5622 300 or 1200 mg/kg chow	2 weeks	No effect*	(Dagher et al., 2015)
Morris Water Maze	CaM/Tet-DTA male and female mice	5-8 months old	PLX3397 290 mg/kg chow	4 weeks	Improved performance in the probe trial*	(Rice et al., 2015)
Morris Water Maze	C57BL/6 male and female mice	2 months old	PLX3397 290 mg/kg chow	3 weeks + 3 week withdrawal	No effect	(Elmore et al., 2015)
Morris Water Maze	WT male and female mice	12 months old	PLX5622 1200 mg/kg chow	3 weeks	Worse performance in the probe trial (more pronounced in females)*	(Unger et al., 2018)
Morris Water Maze andActive avoidance	C57BL6/J female mice	3 months old	PLX5622 1200 mg/kg chow	5 weeks	No effect*	(Willis et al., 2020)
Morris Water Maze	WT male and female	7 months old	PLX5622 1200 mg/kg chow	24 weeks	Improved performance in the probe trial*	(Spangenberg et al., 2019)
Morris Water Maze	C57BL/6 male mice	3 months old	PLX5622 1200 mg/kg chow	2 weeks + 4 weeks withdrawal	No effect	(Elmore et al., 2018)
Morris Water Maze	C57BL/6 male mice	22 months old	PLX5622 1200 mg/kg chow	2 weeks + 4 weeks withdrawal	Improved performance in the probe trial	(Elmore et al., 2018)
Morris Water Maze	C57BL/6J male mice	3 months old	PLX5622 1200 mg/kg chow	1 week + 10 weeks withdrawal	No effect*	(Henry et al., 2020)

Wild-type (WT). *The data refers to control mice with microglia depletion, extracted from studies using animal models of brain pathologies.

Supplementary Table 8. Effects of microglia depletion during adulthood on Working Memory in healthy rodents.

Behavioral task	Animal model	Animal age	Depletion model	Depletion duration	Microglia depletion effects	Reference
Y-maze	C57BL/6 male mice	2 months old	PLX5622 1200 mg/kg chow	1 week	No effect*	(Worthen et al., 2020)
Y-maze	C57BL6/J female mice	3 months old	PLX5622 1200 mg/kg chow	5 weeks	No effect*	(Willis et al., 2020)
Y-maze	C57BL/6 male mice	3 months old	PLX5622 1200 mg/kg chow	9 weeks	No effect*	(Li et al., 2020)
Y-maze	WT male and female mice	7 months old	PLX5622 1200 mg/kg chow	24 weeks	No effect*	(Spangenberg et al., 2019)
Y-maze	C57BL/6J male mice	3 months old	PLX5622 1200 mg/kg chow	1 week + 10 weeks withdrawal	No effect*	(Henry et al., 2020)
Y-maze	$Cx3cr1^{DTR}$ male rats	3 months old	25 ng/g DT (s.c.) twice/day	48h or 1 week recovery	No effect	(De Luca et al., 2020)

Subcutaneous injection (s.c.); wild-type (WT). *The data refers to control mice with microglia depletion, extracted from studies using animal models of brain pathologies.

Supplementary Table 9. Effects of microglia depletion on behaviour in animal models of Adult and Early-life Inflammation

Inflammation model	Animal model	Animal age	Depletion model	Depletion duration	Inflammation effects	Microglia depletion effects	Reference
Local cerebellum inflammation (LPS)	Spragwe-Dawley male rats	Post-natal day 22-26	Ki20227 20 mg/ml, 0.2ml/day (i.g.)	1 week (P19-26)	Increased “anxiety- and depression-like” symptoms, reduced locomotor activity and sociability	Alleviated psychomotor, social, “anxiety-like” and “depression-like” symptoms	(Yamamoto et al., 2019)
Local inflammation (LPS) in substantia nigra and systemic paraquat	G2019S-KI and WT male mice	3 months old	PLX3397 290 mg/kg chow	2 weeks + 2 week withdrawal	Decreased home-cage locomotor activity in WT and especially in G2019S-KI mice	Prevented decrease in locomotor activity in WT and blunted it in G2019S-KI mice	(Dwyer et al., 2020)
Systemic LPS-induced conditioned place aversion	$Cx3cr1^{creER\text{-}hM4Di}$ male mice	2-5 months old	DREADD microglia inactivation; CNO 2 mg/kg (i.p.)	i.p.	LPS-induced conditioned place aversion	Prevented LPS-induced conditioned place aversion	(Klawonn et al., 2021)
Systemic LPS	Spragwe-Dawley male rats	3 months old	PLX5622 1200 mg/kg chow	4 weeks	Reduced voluntary locomotor activity levels	Delayed the recovery of voluntary activity levels	(Vichaya et al., 2020)
Systemic LPS	$Cx3cr1^{DTR}$ Wistar male rats	3 months old	DT 25 ng/g (s.c.) twice/day	2 or 24h recovery	Reduced locomotor activity	Exacerbated the reduction in locomotor activity	(Vichaya et al., 2020)
Maternal immune activation (by poly(I:C))	C57BL/6J male and female mice	Post-natal day 58-68	PLX5622 1200 mg/kg chow	4 weeks (P21-48) + 2 weeks witthdrawal	Reduced sociability and induced repetitive self-grooming behavior in offspring	Prevented repetitive behavior and social deficits	(Ikezu et al., 2021)
Maternal exposure to high-fat diet	F1 hybrid DBA/2J male mice	3 months old	PLX3397 290 mg/kg chow	18 days (P23-45)	Increased impulsive errors (premature responding) in the 5-choice serial reaction time task in males<br><br>Impaired reversal learning in operant task in females	Increased the dropout learning rate in an operant task in control male mice<br><br>Decreased impulsivity (premature responding) in maternal high-fat diet exposed males<br><br>No effect in females	(Smith et al., 2020)
Systemic LPS (P14)	$Cx3cr1^{GFP}$ male mice	Post-natal day 45	PLX3397 290 mg/kg chow	24 Days (P21-45)	Increased “depressive-like” symptoms	Prevented the appearance of “depressive-like” symptoms	(Cao et al., 2021)

Clozapine-N-oxide (CNO); Days (d); diphtheria toxin (DT); Designer Receptor Exclusively activated by a Designer Drug (DREADD); Gram-negative bacterial endotoxin lipopolysaccharide (LPS); intragastrical administration (i.g.); intraperitoneal injection (i.p.); subcutaneous injection (s.c.); wild-type (WT).

Supplementary Table 10. Effects of microglia depletion on behaviour in animal models of Acute and Chronic Stress

Stress model	Animal model	Animal age	Depletion model	Depletion duration	Microglia depletion effects	Reference
Inescapable foot-shocks	$Cx3cr1^{GFP}$ male mice	2-4 months old	PLX3397 290mg/kg chow	3 weeks	Decreased fear expression and "anxiety-like" behaviors	(Li et al., 2021b)
Inescapable foot-shock	$Cx3cr1^{creER}$:iDTR male mice	2-4 months old	Tamoxifen 10mg/mouse (i.g.) 3 times + DT 1µg/mouse (i.p.) 3 days	3 days	Decreased fear expression and "anxiety-like" behaviors	(Li et al., 2021b)
Chronic social defeat	C57BL/6N male mice	2 months old	PLX5622 1200 mg/kg chow	4 weeks + 2 week withdrawal	Decreased "anxiety-like" behaviors and normalized social interaction<br><br>Symptoms re-appeared upon microglia repopulation	(Lehmann et al., 2019)
Chronic social defeat + acute defeat	C57BL/6 male mice	2 months old	PLX5622 1200 mg/kg chow	2 weeks + 3 weeks withdrawal	Decreased "anxiety-like" behaviors<br><br>Symptoms re-appeared upon microglia repopulation	(Weber et al., 2019)
Chronic social defeat	C57BL/6J male mice	1.5 months old	PLX3397 290mg/kg chow	2 weeks	Ineffective to reduce "depressive-like" behaviors and social interaction in stressed mice	(Gu et al., 2021)
Chronic unpredictable stress	C57BL/6 male mice	6 months old	PLX3397 290 mg/kg chow	2 weeks	Ineffective to reduce "depressive-like" and "anxiety-like" behaviors in stressed mice	(Gao et al., 2019)
Chronic unpredictable stress	C57BL/6 male mice	6 months old	PLX3397 290 mg/kg chow	2 weeks	Ineffective to reduce "depressive-like" behaviors in stressed mice	(Cai et al., 2020)
Learned helplessness	C57BL/6 male mice	2 months old	PLX5622 1200 mg/kg chow	1 week	Reduced number of mice expressing learned helplessness<br><br>Alleviated cognitive deficits (Novel Object Recognition and Y-maze) in mice expressing learned helplessness<br><br>Worsened cognitive function (Novel Object Recognition and Y-maze) in stress-resilient mice	(Worthen et al., 2020)

Diphtheria toxin (DT); intragastrical administration (i.g.); intraperitoneal injection (i.p.).

Supplementary Table 11. Effects of microglia depletion on behaviour in animal models of Chronic Pain

Chronic pain model	Animal model	Animal age	Depletion model	Depletion duration	Microglia depletion effects	Reference
High-frequency stimulation of spinal fibers	Cx3cr1$^{CreER/+}$:R26$^{iDTR/+}$ male mice	2-3 months old	Tamoxifen 0.15 g/kg (i.p.) 4 times + DT 50 µg/kg (i.p.) twice/day	3 or 21 days recovery	Prevented, but did not reverse existing, HFS-induced mechanical allodynia	(Zhou et al., 2019)
Partial sciatic nerve ligation	C57BL/6 male mice	2-3 months old	PLX5622 65 mg/kg bw/day (i.g.)	2 weeks	Prevented and reversed existing mechanical and thermal allodynia	(Lee et al., 2018)
Sciatic nerve ligation	Wistar male rats	adult	Clodronate liposomes 30 µg intrathecal injection	2-48h recovery	Prevented, but did not reverse existing, mechanical allodynia	(Wang et al., 2018)
Limb ischemia	Sprague-Dawley male rats	adult	PLX3397 30 mg/kg (i.g.) or chow 50 mg/kg/day	1 week	Reduced mechanical allodynia and thermal hyperalgesia	(Tang et al., 2018)
Thalamic hemorrhage	C57BL/6J male and female mice	2-3 months old	PLX3397 290 mg/kg chow	4-6 weeks	Prevented, but did not reverse existing, mechanical allodynia	(Hiraga et al., 2020)
Chronic social defeat	C57BL/6 male mice	2 months old	PLX3397 1200 mg/kg chow	2 weeks	Prevented mechanical allodynia	(Sawicki et al., 2019)
High-fat diet	C57BL/6CR male mice	1-3 months old	PLX3397 46.25 mg/kg (i.p.)	1 week	Attenuated mechanical allodynia and thermal hyperalgesia	(Liang et al., 2019)
High-fat diet	C57BL/6CR male mice	1-3 months old	Mac-1-saporin 11.2 µg/mouse intrathecal injection	24 h recovery	Attenuated mechanical allodynia and thermal hyperalgesia	(Liang et al., 2019)
Sciatic nerve injury	Sprague-Dawley male adult rats	adult	CD68-hM4Di (inhibitory DREADD) intrathecal + CNO (60 µg)	24 h recovery	Attenuated mechanical allodynia	(Grace et al., 2018)
Partial sciatic nerve ligation	Cx3cr1^{hM4Di} male and female mice	2-3 months old	CNO 10mg/kg (i.p.)	4 days (7-11 after ligation)	Attenuated mechanical allodynia in male, but not female, mice	(Saika et al., 2020)
Spinal nerve transection	Cx3cr1$^{creER/+}$:R26$^{LSL\text{-}hM4Di/+}$ male and female mice	7-12 months old	Tamoxifen 150 mg/kg (i.p.) twice/day + CNO 5 mg/kg 3 days	3 days + 3-10 days recovery	Reduced mechanical hypersensitivity in both male and female	(Yi et al., 2021)

Clozapine-N-oxide (CNO); Designer Receptor Exclusively activated by a Designer Drug (DREADD); diphtheria toxin (DT); gigh-frequency stimulation (HFS); intragastrical administration (i.g.); intraperitoneal injection (i.p.).

Supplementary Table 12. Effects of microglia depletion on behaviour in animal models of Chemotherapy and Radiation

Chemotherapy/ Radiation model	Animal model	Animal age	Depletion model	Depletion duration	Microglia depletion effects	Reference
Methotrexate	BL6/CD1 male and female mice	2 months old	PLX5622 1200 mg/kg chow	4 weeks	Rescued deficits in novel object discrimination learning	(Gibson et al., 2019)
Adriamycin	C57BL6/J male mice	6 months old	PLX5622 1200 mg/kg chow	3-4 weeks	Rescued deficits in novel object discrimination learning and contextual fear learning	(Allen et al., 2019)
Cranial irradiation	C57BL6/J male mice	2 months old	PLX5622 300 mg/kg chow	3 weeks + 4 weeks withdrawal	Prevented deficits in novel object discrimination learning	(Feng et al., 2016)
Cranial irradiation	C57BL6/J male mice	6 months old	PLX5622 1200 mg/kg chow	4 weeks	Rescued deficits in novel object/location discrimination learning (and contextual fear learning	(Acharya et al., 2016)
Whole-body helium irradiation	C57B6/J male mice	5 months old	PLX5622 1200 mg/kg chow	2 weeks + 10 weeks withdrawal	Prevented deficits in novel object discrimination learning	(Krukowski et al., 2018)
Whole-body helium irradiation	C57BL6/J male mice	6 months old	PLX5622 1200 mg/kg chow	3-4 weeks	Rescued deficits in discrimination learning novel object/location and improved fear extinction	(Allen et al., 2020)

Supplementary Table 13. Effects of microglia depletion on behaviour in animal models of Brain Injury and Stroke

Brain injury/ Stroke model	Animal model	Animal age	Depletion model	Depletion duration	Microglia depletion effects	Reference
Neuronal loss	CaM/Tet-DTA male and female mice	5-8 months old	PLX3397 290 mg/kg chow	4 weeks	Normalized “anxiety-like” behaviors and improved spatial learning	(Rice et al., 2015)
Neuronal loss	CaM/Tet-DTA male and female mice	5-8 months old	PLX5622 1200 mg/kg chow	2 weeks + 3 weeks withdrawal	Normalized “anxiety-like” behavior and improved spatial learning	(Rice et al., 2017)
Spinal cord injury	C57BL/6 male mice	3 months old	PLX5622 1200 mg/kg chow	5 weeks	Ameliorated “depressive-like” symptoms<br>Prevented deficits in discrimination learning<br>Modest improvement neuromotor function	(Li et al., 2020)
Traumatic brain injury	C57BL/6 male mice	2 months old	PLX5622 1200 mg/kg chow	2 weeks	Prevented deficits in discrimination learning	(Witcher et al., 2021)
Brain hemorrhage	C57BL/6J male mice	3-4 months old	PLX3397 40 mg/kg (i.g.)	1 week	Rescued deficits in spatial learning and memory	(Shi et al., 2019)
Pilocarpine-induced epilepsy	Spragwe-Dawley male rats	2 months old	PLX3397 455 mg/kg chow	2 weeks	Failed to rescue deficits in discrimination and spatial learning	(Wyatt-Johnson et al., 2021)
Traumatic brain injury	C57BL6/J female mice	3 months old	PLX5622 1200 mg/kg chow	5 weeks	Failed to rescue spatial learning and working memory deficits	(Willis et al., 2020)
Traumatic brain injury	C57BL6/J female mice 3 mo	3 months old	PLX5622 1200 mg/kg chow	3 weeks + 3 weeks withdrawal	Rescued spatial learning and working memory deficits	(Willis et al., 2020)
Traumatic brain injury	Cx3cr1^{creERT2}:iDTR female mice	3 months old	Tamoxifen (12.5 mg/g) 5 days + DT 30 ng/g (i.p.) 3 days (6 weeks later)	3 days	Rescued spatial learning and working memory deficits	(Willis et al., 2020)
Traumatic brain injury	C57BL/6J male mice	3 months old	PLX5622 1200 mg/kg chow	1 week + 9-12 weeks withdrawal	Rescued deficits in spatial memory, discrimination learning and working memory<br>Modest improvement in motor function	(Henry et al., 2020)
Cerebellar hemorrhage	C57BL/6 male mice	2 months old	Ki20227 20 mg/kg (i.g.)	2 weeks	Modest improvement neuromotor function	(Xie et al., 2020)
Brain ischemia	C57BL/6 male mice	2-3 months old	PLX3397 40 mg/kg/day chow	4 weeks	Worsened neurological severity score	(Jin et al., 2017)
Spinal cord injury	C57BL/6 male mice	2 months old	PLX3397 290 mg/kg chow	5 weeks	Worsened neuromotor function	(Fu et al., 2020)

Diphtheria toxin (DT); intragastrical administration (i.g.); intraperitoneal injection (i.p.).

Supplementary Table 14. Effects of microglia depletion on behaviour in animal models of Neurodegenerative and Demyelinating Diseases

Neurodegenerative and Demyelinating disease models	Animal model	Animal age	Depletion model	Depletion duration	Microglia depletion effects	Reference
Lysosomal storage disease	*Ppt1*$^{-/-}$ male and female mice	4-6 months old	PLX3397 150 mg/kg chow	20 weeks	Ameliorated motor deficits (Rotarod) and improved visual acuity	(Berve et al., 2020)
Spinocerebellar ataxia	ATXN1 male and female mice	3 months old	PLX3397 200 mg/kg chow	8 weeks	Ameliorated motor deficits (Rotarod and Balance beam)	(Qu et al., 2017)
Cuprizone demyelinating model	C57BL/6 male	2 months old	PLX3397 290 mg/kg chow	3 weeks	Ameliorated motor deficits (Rotarod)	(Tahmasebi et al., 2021)
White matter progressive inflammation	*Cnp* KO male and female mice	3 months old	PLX5622 1200 mg/kg chow	10 weeks + 5 weeks withdrawal	Worsened motor function (Catatonia)	(Garcia-Agudo et al., 2019)
Amyotrophic lateral sclerosis	Inducible rNLS8 male and female mice	3 months old	PLX3397 1000 mg/kg Nutella	3 weeks	Worsened motor function (Hindlimb clasping)	(Spiller et al., 2018)
Autoimmune encephalitis	Cx3cr1-CreER^{T2}:iDTR	adult mice	Tamoxifen 10 µg/mouse (i.p.) 5 days + 1 µg/mouse DT (i.p.) 3 days	3 days + 5-25 days recovery	Worsened motor function (Ataxia)	(Rubino et al., 2018)
Autoimmune encephalitis	C57BL/6 female mice	2 months old	PLX5622 1200 mg/kg chow	4 weeks	Ameliorated neuromotor deficits	(Nissen et al., 2018)
6-OHDA-induced Parkinson Disease	Spragwe-Dawley rats male	8 months old	PLX3397 30 mg/kg (i.g.)	3 weeks	Ameliorated motor deficits and “depressive-like” symptoms	(Oh et al., 2020)
Rotenone-induced Parkinson Disease	C57BL6/J male mice	2 months old	PLX3397 40 mg/kg/day (i.g.)	3-4 weeks	Ameliorated deficits in spatial memory and discrimination learning	(Zhang et al., 2021)
MPTP-induced Parkinson Disease	C57BL/6L male mice	2 months old	PLX3397 40 mg/kg/day (i.p.)	3 weeks	Worsened motor function	(Yang et al., 2018)
MPTP-induced Parkinson Disease	C57BL6/J male mice	2 months old	PLX3397 290 mg/kg chow	3 weeks + 1 week withdrawal	Ameliorated motor deficits	(Li et al., 2021a)
Alzheimer’s Disease	3xTg-AD mice	15 months old	PLX5622 300 mg/kg chow	6-12 weeks	Improved spatial memory and discrimination learning	(Dagher et al., 2015)
Alzheimer’s Disease	5XFAD male and female mice	7 months old	PLX5622 1200 mg/kg chow	24 weeks	Worsened “anxiety-like” behavior	(Spangenberg et al., 2019)
Alzheimer’s Disease	APP Swedish PS1-dE9 male and female mice	12 months old	PLX5622 1200 mg/kg chow	4 weeks	Failed to improve spatial learning performance	(Unger et al., 2018)

1-methyl-4-phenyl-1,2,3,6-tetrahydropyridine (MPTP); 2’-39-cyclic nucleotide 39-phosphodiesterase (*Cnp*); 6-hydroxydopamine or 2,4,5-trihydroxyphenethylamine (6-OHDA); Amyloid Precursor Protein (APP); Ataxin-1 (ATXN1); diphtheria toxin (DT); intragastrical administration (i.g.); intraperitoneal injection (i.p.); mutant human presenilin 1 (PS1-dE9); palmitoyl-protein thioesterase-1 gene (*Ppt1*).

References

Acharya, M. M., Green, K. N., Allen, B. D., Najafi, A. R., Syage, A., Minasyan, H., et al. (2016). Elimination of microglia improves cognitive function following cranial irradiation. *Sci. Rep.* 6, 31545. doi:10.1038/srep31545.

Adeluyi, A., Guerin, L., Fisher, M. L., Galloway, A., Cole, R. D., Chan, S. S. L., et al. (2019). Microglia morphology and proinflammatory signaling in the nucleus accumbens during nicotine withdrawal. *Sci. Adv.* 5, 1–11. doi:10.1126/sciadv.aax7031.

Allen, B. D., Apodaca, L. A., Syage, A. R., Markarian, M., Baddour, A. A. D., Minasyan, H., et al. (2019). Attenuation of neuroinflammation reverses Adriamycin-induced cognitive impairments. *Acta Neuropathol. Commun.* 7, 1–15. doi:10.1186/s40478-019-0838-8.

Allen, B. D., Syage, A. R., Maroso, M., Baddour, A. A. D., Luong, V., Minasyan, H., et al. (2020). Mitigation of helium irradiation-induced brain injury by microglia depletion. *J. Neuroinflammation* 17, 1–18. doi:10.1186/s12974-020-01790-9.

Ayata, P., Badimon, A., Strasburger, H. J., Duff, M. K., Montgomery, S. E., Loh, Y.-H. H. E. H. E., et al. (2018). Epigenetic regulation of brain region-specific microglia clearance activity. *Nat. Neurosci.* 21, 1049–1060. doi:10.1038/s41593-018-0192-3.

Badimon, A., Strasburger, H. J., Ayata, P., Chen, X., Nair, A., Ikegami, A., et al. (2020a). Negative feedback control of neuronal activity by microglia. *Nature*. doi:10.1038/s41586-020-2777-8.

Badimon, A., Strasburger, H. J., Ayata, P., Chen, X., Nair, A., Ikegami, A., et al. (2020b). Negative feedback control of neuronal activity by microglia. *Nature* 586, 417–423. doi:10.1038/s41586-020-2777-8.

Basilico, B., Ferrucci, L., Ratano, P., Golia, M. T., Grimaldi, A., Rosito, M., et al. (2021). Microglia control glutamatergic synapses in the adult mouse hippocampus. *Glia*, 1–23. doi:10.1002/glia.24101.

Basilico, B., Pagani, F., Grimaldi, A., Cortese, B., Di Angelantonio, S., Weinhard, L., et al. (2019). Microglia shape presynaptic properties at developing glutamatergic synapses. *Glia* 67, 53–67. doi:10.1002/glia.23508.

Berve, K., West, B. L., Martini, R., and Groh, J. (2020). Sex- and region-biased depletion of microglia/macrophages attenuates CLN1 disease in mice. *J. Neuroinflammation* 17, 1–17. doi:10.1186/s12974-020-01996-x.

Cai, Z., Ye, T., Xu, X., Gao, M., Zhang, Y., Wang, D., et al. (2020). Antidepressive properties of microglial stimulation in a mouse model of depression induced by chronic unpredictable stress. *Prog. Neuro-Psychopharmacology Biol. Psychiatry* 101, 109931. doi:10.1016/j.pnpbp.2020.109931.

Cao, P., Chen, C., Liu, A., Shan, Q., Zhu, X., Jia, C., et al. (2021). Early-life inflammation promotes depressive symptoms in adolescence via microglial engulfment of dendritic spines. *Neuron* 109, 2573-2589.e9. doi:10.1016/j.neuron.2021.06.012.

Christie, L. A., Acharya, M. M., Parihar, V. K., Nguyen, A., Martirosian, V., and Limoli, C. L. (2012). Impaired cognitive function and hippocampal neurogenesis following cancer chemotherapy. *Clin. Cancer Res.* 18, 1954–1965. doi:10.1158/1078-0432.CCR-11-2000.

Crapser, J. D., Ochaba, J., Soni, N., Reidling, J. C., Thompson, L. M., and Green, K. N. (2020a). Microglial depletion prevents extracellular matrix changes and striatal volume reduction in a model of Huntington's disease. *Brain* 143, 266–288. doi:10.1093/brain/awz363.

Crapser, J. D., Spangenberg, E. E., Barahona, R. A., Arreola, M. A., Hohsfield, L. A., and Green, K. N. (2020b). Microglia facilitate loss of perineuronal nets in the Alzheimer's disease brain. *EBioMedicine* 58. doi:10.1016/j.ebiom.2020.102919.

Dagher, N. N., Najafi, A. R., Kayala, K. M. N., Elmore, M. R. P., White, T. E., Medeiros, R., et al. (2015). Colony-stimulating factor 1 receptor inhibition prevents microglial plaque association and improves cognition in 3xTg-AD mice. *J. Neuroinflammation* 12, 1–14. doi:10.1186/s12974-015-0366-9.

De Luca, S. N., Soch, A., Sominsky, L., Nguyen, T. X., Bosakhar, A., and Spencer, S. J. (2020). Glial remodeling enhances short-term memory performance in Wistar rats. *J. Neuroinflammation* 17, 1–18. doi:10.1186/s12974-020-1729-4.

De Luca, S. N., Sominsky, L., Soch, A., Wang, H., Ziko, I., Rank, M. M., et al. (2019). Conditional microglial depletion in rats leads to reversible anorexia and weight loss by disrupting gustatory circuitry. *Brain. Behav. Immun.* 77, 77–91. doi:10.1016/j.bbi.2018.12.008.

Dwyer, Z., Rudyk, C., Situt, D., Beauchamp, S., Abdali, J., Dinesh, A., et al. (2020). Microglia depletion prior to lipopolysaccharide and paraquat treatment differentially modulates behavioral and neuronal outcomes in wild type and G2019S LRRK2 knock-in mice. *Brain, Behav. Immun. - Heal.* 5, 100079. doi:10.1016/j.bbih.2020.100079.

Elmore, M. R. P., Hohsfield, L. A., Kramár, E. A., Soreq, L., Lee, R. J., Pham, S. T., et al. (2018). Replacement of microglia in the aged brain reverses cognitive, synaptic, and neuronal deficits in mice. *Aging Cell* 17. doi:10.1111/acel.12832.

Elmore, M. R. P., Lee, R. J., West, B. L., and Green, K. N. (2015). Characterizing Newly Repopulated Microglia in the Adult Mouse : Impacts on Animal Behavior , Cell Morphology , and Neuroinflammation. *PLoS One* 10(4), e0122912. doi:10.1371/journal.pone.0122912.

Elmore, M. R. P., Najafi, A. R., Koike, M. A., Dagher, N. N., Spangenberg, E. E., Rice, R. A., et al. (2014). Colony-stimulating factor 1 receptor signaling is necessary for microglia viability, unmasking a microglia progenitor cell in the adult brain. *Neuron* 82, 380–397. doi:10.1016/j.neuron.2014.02.040.

Feng, X., Jopson, T. D., Paladini, M. S., Liu, S., West, B. L., Gupta, N., et al. (2016). Colony-stimulating factor 1 receptor blockade prevents fractionated whole-brain irradiation-induced memory deficits. *J. Neuroinflammation* 13, 1–13. doi:10.1186/s12974-016-0671-y.

Fu, H., Zhao, Y., Hu, D., Wang, S., Yu, T., and Zhang, L. (2020). Depletion of microglia exacerbates injury and impairs function recovery after spinal cord injury in mice. *Cell Death Dis.* 11. doi:10.1038/s41419-020-2733-4.

Gao, M., Hu, P., Cai, Z., Wu, Y., Wang, D., Hu, W., et al. (2019). Identification of a microglial activation-dependent antidepressant effect of amphotericin B liposome. *Neuropharmacology* 151, 33–44. doi:10.1016/j.neuropharm.2019.04.005.

Garcia-Agudo, L. F., Janova, H., Sendler, L. E., Arinrad, S., Steixner, A. A., Hassouna, I., et al. (2019). Genetically induced brain inflammation by Cnp deletion transiently benefits from microglia depletion. *FASEB J.* 33, 8634–8647. doi:10.1096/fj.201900337R.

Gibson, E. M., Nagaraja, S., Ocampo, A., Tam, L. T., Wood, L. S., Pallegar, P. N., et al. (2019). Methotrexate Chemotherapy Induces Persistent Tri-glial Dysregulation that Underlies Chemotherapy-Related Cognitive Impairment. *Cell* 176, 43-55.e13. doi:10.1016/j.cell.2018.10.049.

Grace, P. M., Wang, X., Strand, K. A., Baratta, M. V., Zhang, Y., Galer, E. L., et al. (2018). DREADDed microglia in pain: Implications for spinal inflammatory signaling in male rats. *Exp. Neurol.* 304, 125–131. doi:10.1016/j.expneurol.2018.03.005.

Gu, Y., Ye, T., Tan, P., Tong, L., Ji, J., Gu, Y., et al. (2021). Tolerance-inducing effect and properties of innate immune stimulation on chronic stress-induced behavioral abnormalities in mice. *Brain. Behav. Immun.* 91, 451–471. doi:10.1016/j.bbi.2020.11.002.

Henry, R. J., Ritzel, R. M., Barrett, J. P., Doran, S. J., Jiao, Y., Leach, J. B., et al. (2020). Microglial depletion with CSF1R inhibitor during chronic phase of experimental traumatic brain injury reduces neurodegeneration and neurological deficits. *J. Neurosci.* 40, 2960–2974. doi:10.1523/JNEUROSCI.2402-19.2020.

Hiraga, S. I., Itokazu, T., Hoshiko, M., Takaya, H., Nishibe, M., and Yamashita, T. (2020). Microglial depletion under thalamic hemorrhage ameliorates mechanical allodynia and suppresses aberrant axonal sprouting. *JCI Insight* 5. doi:10.1172/jci.insight.131801.

Ikezu, S., Yeh, H., Delpech, J. C., Woodbury, M. E., Van Enoo, A. A., Ruan, Z., et al. (2021). Inhibition of colony stimulating factor 1 receptor corrects maternal inflammation-induced microglial and synaptic dysfunction and behavioral abnormalities. *Mol. Psychiatry* 26, 1808–1831. doi:10.1038/s41380-020-0671-2.

Jin, W. N., Shi, S. X. Y., Li, Z., Li, M., Wood, K., Gonzales, R. J., et al. (2017). Depletion of microglia exacerbates postischemic inflammation and brain injury. *J. Cereb. Blood Flow Metab.* 37, 2224–2236. doi:10.1177/0271678X17694185.

Kakae, M., Tobori, S., Morishima, M., Nagayasu, K., Shirakawa, H., and Kaneko, S. (2019). Depletion of microglia ameliorates white matter injury and cognitive impairment in a mouse chronic cerebral hypoperfusion model. *Biochem. Biophys. Res. Commun.* 514, 1040–1044. doi:10.1016/j.bbrc.2019.05.055.

Klawonn, A. M., Fritz, M., Castany, S., Pignatelli, M., Canal, C., Similä, F., et al. (2021). Microglial activation elicits a negative affective state through prostaglandin-mediated modulation of striatal neurons. *Immunity* 54, 225-234.e6. doi:10.1016/j.immuni.2020.12.016.

Krukowski, K., Feng, X., Paladini, M. S., Chou, A., Sacramento, K., Grue, K., et al. (2018). Temporary microglia-depletion after cosmic radiation modifies phagocytic activity and prevents cognitive deficits. *Sci. Rep.* 8, 1–13. doi:10.1038/s41598-018-26039-7.

Lee, S. H., Shi, X. Q., Fan, A., West, B., and Zhang, J. (2018). Targeting macrophage and microglia activation with colony stimulating factor 1 receptor inhibitor is an effective strategy to treat injury-triggered neuropathic pain. *Mol. Pain* 14. doi:10.1177/1744806918764979.

Lehmann, M. L., Weigel, T. K., Poffenberger, C. N., and Herkenham, M. (2019). The behavioral sequelae of social defeat require microglia and are driven by oxidative stress in mice. *J. Neurosci.* 39, 5594–5605. doi:10.1523/JNEUROSCI.0184-19.2019.

Li, Q., Shen, C., Liu, Z., Ma, Y., Wang, J., Dong, H., et al. (2021a). Partial depletion and repopulation of microglia have different effects in the acute MPTP mouse model of Parkinson's disease. *Cell Prolif.* 54, 1–16. doi:10.1111/cpr.13094.

Li, S., Liao, Y., Dong, Y., Li, X., Li, J., Cheng, Y., et al. (2021b). Microglial deletion and inhibition alleviate behavior of post-traumatic stress disorder in mice. *J. Neuroinflammation* 18, 1–14. doi:10.1186/s12974-020-02069-9.

Li, Y., Ritzel, R. M., Khan, N., Cao, T., He, J., Lei, Z., et al. (2020). Delayed microglial depletion after spinal cord injury reduces chronic inflammation and neurodegeneration in the brain and improves neurological recovery in male mice. *Theranostics* 10, 11376–11403. doi:10.7150/thno.49199.

Liang, Y. J., Feng, S. Y., Qi, Y. P., Li, K., Jin, Z. R., Jing, H. B., et al. (2019). Contribution of microglial reaction to increased nociceptive responses in high-fat-diet (HFD)-induced obesity in male mice. *Brain. Behav. Immun.* 80, 777–792. doi:10.1016/j.bbi.2019.05.026.

Nissen, J. C., Thompson, K. K., West, B. L., and Tsirka, S. E. (2018). Csf1R inhibition attenuates experimental autoimmune encephalomyelitis and promotes recovery. *Exp. Neurol.* 307, 24–36. doi:10.1016/j.expneurol.2018.05.021.

Oh, S. J., Ahn, H., Jung, K. H., Han, S. J., Nam, K. R., Kang, K. J., et al. (2020). Evaluation of the Neuroprotective Effect of Microglial Depletion by CSF-1R Inhibition in a Parkinson's Animal Model. *Mol. Imaging Biol.* 22, 1031–1042. doi:10.1007/s11307-020-01485-w.

Parihar, V. K., Allen, B. D., Tran, K. K., Chmielewski, N. N., Craver, B. M., Martirosian, V., et al. (2015). Targeted overexpression of mitochondrial catalase prevents radiation-induced cognitive dysfunction. *Antioxidants Redox Signal.* 22, 78–91. doi:10.1089/ars.2014.5929.

Parkhurst, C. N., Yang, G., Ninan, I., Savas, J. N., Yates, J. R., Lafaille, J. J., et al. (2013). Microglia promote learning-dependent synapse formation through brain-derived neurotrophic factor. *Cell* 155, 1596–1609. doi:10.1016/J.CELL.2013.11.030.

Pinto, B., Morelli, G., Rastogi, M., Savardi, A., Fumagalli, A., Petretto, A., et al. (2020). Rescuing Over-activated Microglia Restores Cognitive Performance in Juvenile Animals of the Dp(16) Mouse Model of Down Syndrome. *Neuron* 108, 887-904.e12. doi:10.1016/j.neuron.2020.09.010.

Qu, W., Johnson, A., Kim, J. H., Lukowicz, A., Svedberg, D., and Cvetanovic, M. (2017). Inhibition of colony-stimulating factor 1 receptor early in disease ameliorates motor deficits in SCA1 mice. *J. Neuroinflammation* 14, 1–11. doi:10.1186/s12974-017-0880-z.

Rice, R. A., Pham, J., Lee, R. J., Najafi, A. R., West, B. L., and Green, K. N. (2017). Microglial repopulation resolves inflammation and promotes brain recovery after injury. *Glia* 65, 931–944.

doi:10.1002/glia.23135.
Rice, R. A., Spangenberg, E. E., Yamate-Morgan, H., Lee, R. J., Arora, R. P. S., Hernandez, M. X., et al. (2015). Elimination of microglia improves functional outcomes following extensive neuronal loss in the hippocampus. *J. Neurosci.* 35, 9977–9989. doi:10.1523/JNEUROSCI.0336-15.2015.
Rubino, S. J., Mayo, L., Wimmer, I., Siedler, V., Brunner, F., Hametner, S., et al. (2018). Acute microglia ablation induces neurodegeneration in the somatosensory system. *Nat. Commun.* 9. doi:10.1038/s41467-018-05929-4.
Saika, F., Matsuzaki, S., Kobayashi, D., Ideguchi, Y., Nakamura, T. Y., Kishioka, S., et al. (2020). Chemogenetic Regulation of CX3CR1-Expressing Microglia Using Gi-DREADD Exerts Sex-Dependent Anti-Allodynic Effects in Mouse Models of Neuropathic Pain. *Front. Pharmacol.* 11, 925. doi:10.3389/fphar.2020.00925.
Sawicki, C. M., Kim, J. K., Weber, M. D., Faw, T. D., McKim, D. B., Madalena, K. M., et al. (2019). Microglia promote increased pain behavior through enhanced inflammation in the spinal cord during repeated social defeat stress. *J. Neurosci.* 39, 1139–1149. doi:10.1523/JNEUROSCI.2785-18.2018.
Shi, E., Shi, K., Qiu, S., Sheth, K. N., Lawton, M. T., and Ducruet, A. F. (2019). Chronic inflammation, cognitive impairment, and distal brain region alteration following intracerebral hemorrhage. *FASEB J.* 33, 9616–9626. doi:10.1096/fj.201900257R.
Smith, B. L., Laaker, C. J., Lloyd, K. R., Hiltz, A. R., and Reyes, T. M. (2020). Adolescent microglia play a role in executive function in male mice exposed to perinatal high fat diet. *Brain. Behav. Immun.* 84, 80–89. doi:10.1016/j.bbi.2019.11.010.
Spangenberg, E., Severson, P. L., Hohsfield, L. A., Crapser, J., Zhang, J., Burton, E. A., et al. (2019). Sustained microglial depletion with CSF1R inhibitor impairs parenchymal plaque development in an Alzheimer's disease model. *Nat. Commun.* 10, 1–21. doi:10.1038/s41467-019-11674-z.
Spiller, K. J., Restrepo, C. R., Khan, T., Dominique, M. A., Fang, T. C., Canter, R. G., et al. (2018). Microglia-mediated recovery from ALS-relevant motor neuron degeneration in a mouse model of TDP-43 proteinopathy. *Nat. Neurosci.* 21, 329–340. doi:10.1038/s41593-018-0083-7.
Tahmasebi, F., Pasbakhsh, P., Barati, S., Madadi, S., and Kashani, I. R. (2021). The effect of microglial ablation and mesenchymal stem cell transplantation on a cuprizone-induced demyelination model. *J. Cell. Physiol.* 236, 3552–3564. doi:10.1002/jcp.30090.
Tang, Y., Liu, L., Xu, D., Zhang, W., Zhang, Y., Zhou, J., et al. (2018). Interaction between astrocytic colony stimulating factor and its receptor on microglia mediates central sensitization and behavioral hypersensitivity in chronic post ischemic pain model. *Brain. Behav. Immun.* 68, 248–260. doi:10.1016/j.bbi.2017.10.023.
Torres, L., Danver, J., Ji, K., Miyauchi, J. T., Chen, D., Anderson, M. E., et al. (2016). Dynamic microglial modulation of spatial learning and social behavior. *Brain. Behav. Immun.* 55, 6–16. doi:10.1016/j.bbi.2015.09.001.
Unger, M. S., Schernthaner, P., Marschallinger, J., Mrowetz, H., and Aigner, L. (2018). Microglia prevent peripheral immune cell invasion and promote an anti-inflammatory environment in the brain of APP-PS1 transgenic mice. *J. Neuroinflammation* 15, 1–23. doi:10.1186/s12974-018-1304-4.
Vichaya, E. G., Malik, S., Sominsky, L., Ford, B. G., Spencer, S. J., and Dantzer, R. (2020). Microglia depletion fails to abrogate inflammation-induced sickness in mice and rats. *J. Neuroinflammation* 17, 1–14. doi:10.1186/s12974-020-01832-2.
Wang, C., Yue, H., Hu, Z., Shen, Y., Ma, J., Li, J., et al. (2020). Microglia mediate forgetting via complement-dependent synaptic elimination. *Science (80-.).* 367, 688–694. doi:10.1126/science.aaz2288.
Wang, X., Zhao, L., Zhang, J., Fariss, R. N., Ma, W., Kretschmer, F., et al. (2016). Requirement for microglia for the maintenance of synaptic function and integrity in the mature retina. *J. Neurosci.* 36, 2827–2842. doi:10.1523/JNEUROSCI.3575-15.2016.
Wang, Y. R., Mao, X. F., Wu, H. Y., and Wang, Y. X. (2018). Liposome-encapsulated clodronate specifically depletes spinal microglia and reduces initial neuropathic pain. *Biochem. Biophys. Res. Commun.* 499, 499–505. doi:10.1016/j.bbrc.2018.03.177.
Weber, M. D., McKim, D. B., Niraula, A., Witcher, K. G., Yin, W., Sobol, C. G., et al. (2019). The Influence of Microglial Elimination and Repopulation on Stress Sensitization Induced by Repeated Social Defeat. *Biol. Psychiatry* 85, 667–678. doi:10.1016/j.biopsych.2018.10.009.
Willis, E. F., MacDonald, K. P. A., Nguyen, Q. H., Garrido, A. L., Gillespie, E. R., Harley, S. B. R., et al. (2020). Repopulating Microglia Promote Brain Repair in an IL-6-Dependent Manner. *Cell* 180, 833-846.e16. doi:10.1016/j.cell.2020.02.013.
Witcher, K. G., Bray, C. E., Chunchai, T., Zhao, F., O'Neil, S. M., Gordillo, A. J., et al. (2021). Traumatic brain injury causes chronic cortical inflammation and neuronal dysfunction mediated by Microglia. *J. Neurosci.* 41, 1597–1616. doi:10.1523/JNEUROSCI.2469-20.2020.
Worthen, R. J., Garzon Zighelboim, S. S., Torres Jaramillo, C. S., and Beurel, E. (2020). Anti-inflammatory IL-10 administration rescues depression-associated learning and memory deficits in mice. *J. Neuroinflammation* 17, 1–16. doi:10.1186/s12974-020-01922-1.
Wu, W., Li, Y., Wei, Y., Bosco, D. B., Xie, M., Zhao, M. G., et al. (2020). Microglial depletion aggravates the severity of acute and chronic seizures in mice. *Brain. Behav. Immun.* 89, 245–255. doi:10.1016/j.bbi.2020.06.028.
Wyatt-Johnson, S. K., Sommer, A. L., Shim, K. Y., and Brewster, A. L. (2021). Suppression of Microgliosis With the Colony-Stimulating Factor 1 Receptor Inhibitor PLX3397 Does Not Attenuate Memory Defects During Epileptogenesis in the Rat. *Front. Neurol.* 12, 1–13. doi:10.3389/fneur.2021.651096.
Xie, S. T., Chen, A. X., Song, B., Fan, J., Li, W., Xing, Z., et al. (2020). Suppression of microglial activation and monocyte infiltration ameliorates cerebellar hemorrhage induced-brain injury and ataxia. *Brain. Behav. Immun.* 89, 400–413. doi:10.1016/j.bbi.2020.07.027.
Yamamoto, M., Kim, M., Imai, H., Itakura, Y., and Ohtsuki, G. (2019). Microglia-Triggered Plasticity of Intrinsic Excitability Modulates Psychomotor Behaviors in Acute Cerebellar Inflammation. *Cell Rep.* 28, 2923-2938.e8. doi:10.1016/j.celrep.2019.07.078.

Yang, X., Ren, H., Wood, K., Li, M., Qiu, S., Shi, F. D., et al. (2018). Depletion of microglia augments the dopaminergic neurotoxicity of MPTP. *FASEB J.* 32, 3336–3345. doi:10.1096/fj.201700833RR.
Yegla, B., Boles, J., Kumar, A., and Foster, T. C. (2021). Partial microglial depletion is associated with impaired hippocampal synaptic and cognitive function in young and aged rats. *Glia* 69, 1494–1514. doi:10.1002/GLIA.23975.
Yi, M. H., Liu, Y. U., Liu, K., Chen, T., Bosco, D. B., Zheng, J., et al. (2021). Chemogenetic manipulation of microglia inhibits neuroinflammation and neuropathic pain in mice. *Brain. Behav. Immun.* 92, 78–89. doi:10.1016/j.bbi.2020.11.030.
Zhang, D., Li, S., Hou, L., Jing, L., Ruan, Z., Peng, B., et al. (2021). Microglial activation contributes to cognitive impairments in rotenone-induced mouse Parkinson's disease model. *J. Neuroinflammation* 18, 1–16. doi:10.1186/s12974-020-02065-z.
Zhou, L. J., Peng, J., Xu, Y. N., Zeng, W. J., Zhang, J., Wei, X., et al. (2019). Microglia Are Indispensable for Synaptic Plasticity in the Spinal Dorsal Horn and Chronic Pain. *Cell Rep.* 27, 3844-3859.e6. doi:10.1016/j.celrep.2019.05.087.
